# Prefrontal–thalamic goal states organize spatially aligned hippocampal maps

**DOI:** 10.1038/s41467-026-77240-6

**Published:** 2026-08-29

**Authors:** Zahra Golipour, Marjan Mozaffarilegha, Shao-Fan Yen, Cansu Üstüner, Hiroshi T. Ito

**Affiliations:** 1 Max Planck Institute for Brain Research, Frankfurt am Main, Germany; 2 Department of Fundamental Neurosciences, University of Lausanne, Lausanne, Switzerland

**Keywords:** Hippocampus, Neural circuits

## Abstract

Animals repeatedly traverse the same environment to pursue different goals, yet the hippocampus must preserve a stable spatial map while keeping individual experiences distinct. Here we show that, when male rats navigate the same maze under different goal configurations, hippocampal CA1 segregates navigation experiences by encoding goal state along a population dimension largely orthogonal to the spatial coding subspace, allowing goal-configuration-specific maps to remain spatially aligned. This goal-state signal is also represented in the medial prefrontal cortex (mPFC) and nucleus reuniens (NR), where population activity forms persistent representations across locomotion and immobility and is reliably reinstated when previously experienced goal configurations recur. Silencing NR reduces CA1 goal-state coding, diminishing goal-axis separation and goal-biased pre-navigation spike sequences while sparing spatial coding. Together, these findings identify a circuit- and population-level mechanism that enables goal-defined hippocampal representations to coexist within spatially aligned maps independent of locomotor state, linking internal goal states to navigation and planning.

## Abstract (web summary)

The hippocampus must maintain stable spatial coding while separating distinct experiences at the same locations. Here, the authors show that a prefrontal–thalamic circuit sends goal-state signals to CA1 along an axis orthogonal to the spatial code.

## Introduction

A central challenge for memory systems is to distinguish experiences that occur within the same physical environment^1–6^. The hippocampus is well known for its spatial coding properties^2,7,8^, yet spatially similar or even identical trajectories can belong to different behavioral episodes. How the hippocampal network preserves a stable spatial map while distinguishing such episodes remains unclear. Classical accounts emphasize activity changes driven by sensory, motor, or contextual modulation^9–23^, but such mechanisms typically alter spatial representations themselves, making it unclear how multiple episodes can coexist within the same environment without mutual interference.

Recent work has shown that hippocampal population activity can incorporate non-spatial variables, including internal states, task demands, and latent cognitive dimensions^24,25^. These findings raise the possibility that the hippocampus may separate episodes by allocating them to distinct subspaces of its population activity. However, it remains unknown whether a dedicated population dimension exists that can partition episodes while preserving the integrity of spatial coding subspaces and, critically, which upstream circuits supply the internal contextual signals required to drive such partitioning. Identifying this mechanism is essential for understanding how the hippocampus maintains stable maps while flexibly supporting planning, decision-making, and episodic recall^26–31^.

A further challenge in linking hippocampal function to memory is the dependence of hippocampal activity on locomotion. Hippocampal neural dynamics differ profoundly between locomotion and immobility^32–35^, yet episodic states must be maintained irrespective of movement. The mechanisms that support such locomotion-invariant state representations in hippocampal population activity have remained elusive.

Prefrontal–thalamic circuits are strong candidates for generating these internally defined contextual states^36–40^. The medial prefrontal cortex (mPFC) and the thalamic nucleus reuniens (NR) form a pathway that regulates hippocampal states and is implicated in working memory, behavioral flexibility, and goal-directed planning^17,41–44^. However, whether this pathway contributes to a stable and reinstatable hippocampal goal-state representation—capable of partitioning episodes independently of ongoing behavior—remains unknown. Such a mechanism would offer a principled solution to the interference problem, allowing multiple goal-state-specific yet spatially consistent hippocampal maps to coexist within the same environment.

These considerations motivate the hypothesis that goal-state information represented in the mPFC–NR circuit is integrated into the hippocampal population code along a neural dimension orthogonal to the spatial coding subspace, enabling the segregation of goal-state-specific maps without altering spatial structure. Moreover, if this externally supplied state signal is independent of locomotor state, it could enable the hippocampus to maintain a locomotion-invariant neural state that is dissociable from spatial position coding.

To test this hypothesis, we developed a goal-directed navigation task in a linear maze in which rats traverse identical spatial trajectories while reward locations change across blocks. This design isolates the impact of goal-configuration changes on neural dynamics by ensuring that spatial and sensory inputs remain constant—an approach that is difficult to achieve in conventional two-dimensional maze environments. Using large-scale electrophysiological recordings from mPFC, NR, and hippocampal CA1, combined with optogenetic perturbations of NR neurons, we examined how goal-related information is incorporated into the population coding structure of each region and how it shapes goal-state-specific hippocampal maps. Crucially, we further assessed whether this goal-state code is expressed across both locomotion and immobility, thereby unifying goal-state-specific representations across locomotor states. This framework enabled us to identify a circuit-level mechanism that allows spatially aligned yet goal-state-specific hippocampal maps to coexist and to determine how such maps are selected during planning and navigation.

## Results

### CA1 spatial coding is stable across journeys to different goals

**Fig. 1:**
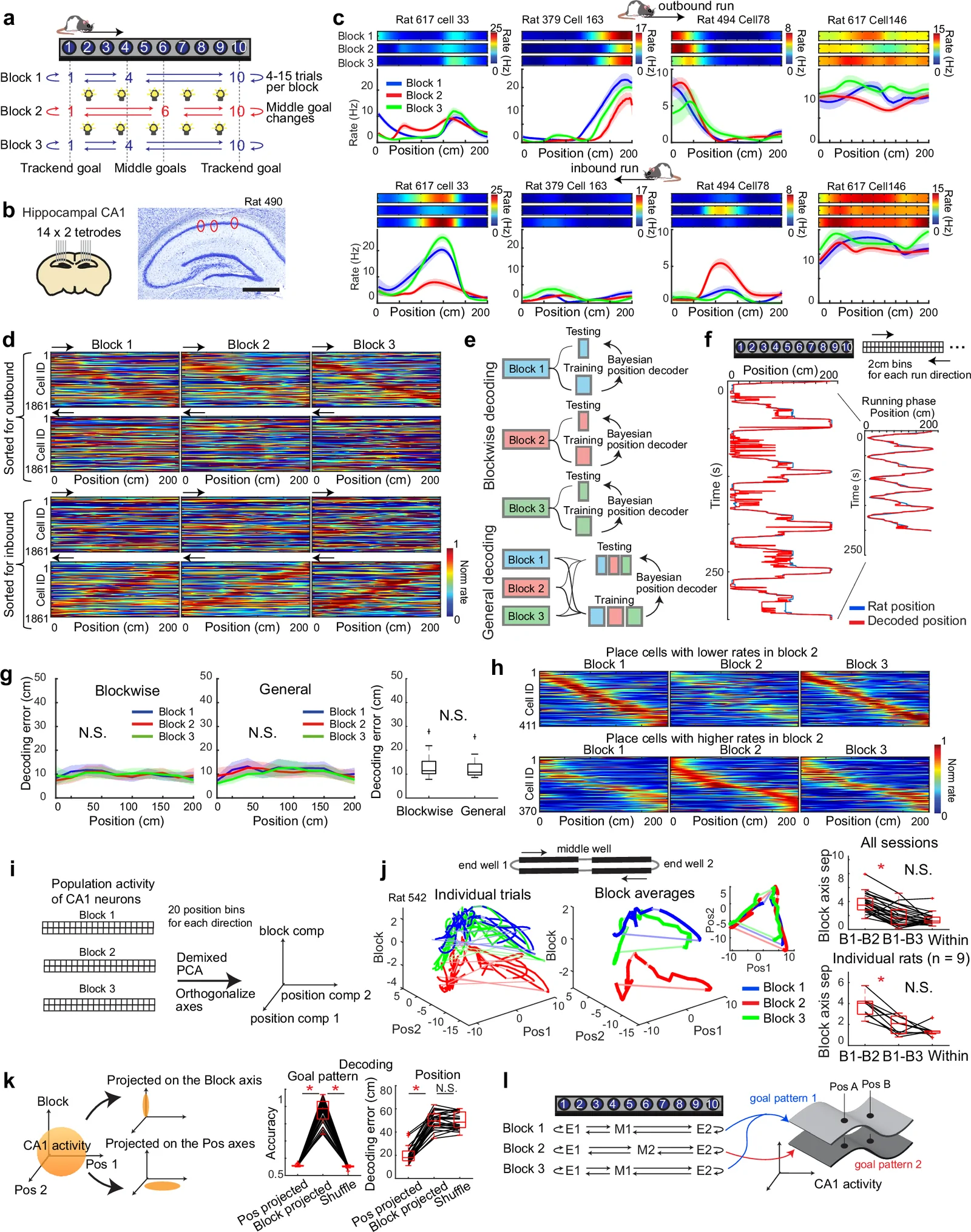
CA1 segregates distinct goal-directed navigation episodes into spatially aligned maps. **a** Schematic of the navigation task showing the maze design and rules. The animal sequentially navigates three goal locations; one complete cycle comprises four goal-directed journey segments. **b** A microdrive carrying 28 tetrodes was implanted bilaterally in hippocampal CA1. Right, Nissl-stained coronal section with red circles marking tetrode tracks. Scale bar = 1 mm. **c** Firing-rate maps of four representative CA1 neurons across three blocks, shown separately for each running direction. **d** Color-coded firing-rate maps of all CA1 neurons per block and running direction, sorted by firing-field location: top two panels for outbound journeys, bottom two for inbound. **e** Schematic of the Bayesian position-decoding strategy. Blockwise decoding trained and tested within individual blocks; general decoding pooled all blocks with randomly interleaved training and testing. **f** Example traces of actual versus decoded position. **g** Decoding errors by maze position across blocks. Box plot (right): no significant difference in mean errors between decoders (*p* > 0.05, *n* = 25, Wilcoxon signed-rank test). **h** Firing-rate maps of all CA1 place cells, split by neurons with lower or higher firing in block 2 relative to blocks 1 and 3. **i** Schematic of the demixed PCA approach extracting two position components and one block component, followed by axis orthogonalization. **j**, 3D projections of CA1 population activity onto block and position components. Left, individual trials; right, block-averaged (inset: position-component traces). Block 2 separates from blocks 1 and 3 along the block axis while overlapping along position axes. Box plots quantify axis separation (blocks 1–2 vs 1–3) and maximum within-block separation across 25 sessions (*p < 0.001, *p* = 0.145) or 9 rats (*p = 0.008, *p* = 0.461). Wilcoxon signed-rank test. **k** Information content per projection axis, assessed by decoding (right). Goal-related and spatial information occupy separate axes. *p < 0.05, *n* = 25, Wilcoxon signed-rank test; chance from class-label shuffling. **l** Schematic of the orthogonal coding scheme: CA1 forms two spatially aligned maps preserving spatial consistency while segregating navigation episodes toward different goals.

To examine how navigational goals shape hippocampal activity, we recorded CA1 neurons using a microdrive carrying 28 independently movable tetrodes bilaterally in dorsal CA1 while rats performed a goal-directed navigation task on a linear maze equipped with ten equidistant water-delivery wells (1861 neurons from 9 rats, 207 ± 13 neurons per animal per session; Fig. 1a, b and Extended Data Figs. 1, 2). Throughout the task, rats navigated a sequence of three goal wells—two fixed at the track ends and a middle goal that was held fixed within a block (4–15 trials) and changed across blocks. Each trial comprised four journeys between successive goal wells in the sequence, encompassing both outbound and inbound runs. A block with new goals began with an introductory trial in which water was pre-delivered at the upcoming goal well, and LEDs under all ten wells were illuminated to signal the goal change. After this initial pre-rewarded trial, rats were required to lick the designated goal well for a fixed duration (0.8–1.5 s) before receiving water, ensuring that correct performance depended on estimating the goal location.

Each recording session consisted of three consecutive blocks. Blocks 1 and 3 shared the same goal configuration, whereas block 2 featured a different middle goal. For each session, the middle goals were randomly selected from wells 4–7. All wells were identical in shape, and behavioral sessions were conducted under minimal lighting to ensure that navigation was not directly guided by specific visual cues. Overall, the animals performed the task with high accuracy (86.0 ± 1.8%) and exhibited smooth goal-targeting without slowing in the middle of journeys (Extended Data Fig. 3a).

We first assessed the spatial tuning of individual CA1 neurons as rats targeted different middle-goal wells in the maze. We found that the majority of place cells maintained consistent spatial tuning despite changes in goal location, although they exhibited pronounced differences between the two running directions (Figs. 1c and 1d). Consistent with this directional dependence, a Bayesian decoder of the animal’s position^26,45^ trained on one running direction performed significantly worse when tested on the opposite direction (Extended Data Fig. 3f), in line with previous studies demonstrating largely independent spatial codes between running directions^46,47^. Given this directional specificity, we quantified the proportions of spatially and non-spatially tuned neurons separately for each running direction (Extended Data Fig. 3g).

Among spatially tuned CA1 neurons (spatial information > 0.25 bits/spike), 31.8% did not maintain stable firing between the identical-goal blocks (blocks 1 and 3), and 9.3% exhibited global remapping in response to changes in goal location. However, the majority of spatial neurons (58.9%) maintained stable spatial fields across trial blocks despite changes in the middle-goal position.

To further validate this observation at the population level, we tested whether the animal’s position could be decoded consistently from CA1 ensemble activity across trial blocks. We applied a Bayesian decoder trained and tested on CA1 population activity (Fig. 1e, f and Extended Data Fig. 3b–e). To assess whether CA1 neurons preserve accurate position coding despite goal changes, we compared position estimation errors from decoders trained separately on each block (“blockwise decoder”) with those from a decoder trained across blocks with different goal configurations (“general decoder”). Because spatial coding differs between the two running directions, we constructed separate decoding templates for each direction and applied them to each time bin, yielding estimates of both the animal’s position and running direction (Extended Data Fig. 3d).

We found that the blockwise and general decoders yielded comparable decoding accuracy across individual blocks (Fig. 1g; decoding errors: blockwise, 12.7 ± 0.9 cm; general, 12.6 ± 0.8 cm; *p* = 0.677, Wilcoxon signed-rank test). Moreover, a decoder trained on any single block accurately estimated positions during the remaining blocks (Extended Data Fig. 3e). These results confirm that CA1 spatial coding remains stable across journeys targeting different goals within the same environment.

### CA1 separates goal states along a dimension orthogonal to spatial coding

The stability of position decoding does not imply that CA1 activity is insensitive to changes in goal location. A substantial subset of place cells with stable spatial tuning exhibited systematic changes in peak firing rate—either increases or decreases—during block 2 relative to blocks 1 and 3 (Fig. 1h), implying that these rate differences reflect changes in the middle-goal location. Comparable modulations of place-cell firing have been described as “rate remapping,” in which firing rates vary with the sensory or contextual features of an environment while spatial tuning is preserved^10^. More broadly, it has been proposed that information about behavioral context—including past and future trajectories and temporal sequences—can be embedded in place-cell activity primarily through changes in peak firing rate^17–20,48^. The resemblance between this goal-dependent rate modulation and previously described “splitter-cell” coding suggests that it could, in principle, arise from either a temporally graded context signal or a discrete latent-state representation^49^.

To distinguish these possibilities, we first quantified the prevalence of goal-dependent firing-rate modulation across CA1 neurons. Among neurons that exhibited consistent spatial tuning across blocks, 40.2% showed significant rate changes within their firing fields corresponding to differences in goal location (Extended Data Fig. 3g). Comparable goal-dependent rate changes were also observed in 32.0% of non-spatial cells (spatial information < 0.25 bits/spike; Extended Data Fig. 3g). To evaluate the impact of rate remapping on population-level activity, we compared population vector (PV) correlations of ensemble firing patterns for spatial and non-spatial cells. PV correlations were significantly lower between blocks with different goals (blocks 1 and 2) than between blocks with the same goals (blocks 1 and 3) for both spatial and non-spatial cells (spatial cells: blocks 1–2, 0.614 ± 0.028; blocks 1–3, 0.774 ± 0.018; non-spatial cells: blocks 1–2, 0.232 ± 0.020; blocks 1–3, 0.337 ± 0.033; *p* < 0.001 for both cell types; Friedman test; Extended Data Fig. 3h). Thus, changes in goal configuration modulate CA1 firing rates at both the single-neuron and ensemble levels while spatial tuning remains largely preserved.

We next asked whether these goal-related changes formed a temporally graded position-dependent code or occupied a separable state dimension. To test this directly, we applied demixed principal component analysis (dPCA)^50^, a linear dimensionality-reduction method that separates population activity into components associated with predefined task parameters (Fig. 1i). Using dPCA, we decomposed CA1 ensemble activity into two sets of task-relevant components capturing spatial position (position components) and goal-block identity (block components), retaining the largest block component and the two largest position components. Projecting population activity onto these components revealed a low-dimensional structure jointly organized by block and position. The block component accounted for 4.9 ± 0.4% of the variance, whereas the two position components together explained 40.1 ± 1.5%. Although dPCA does not enforce orthogonality among components, we verified that these components were nearly orthogonal (Extended Data Fig. 3i). We further applied a Gram–Schmidt procedure to ensure orthogonality of the three components for visualization (see Methods).

Within this low-dimensional space, positional information was captured by the two position axes, whereas differences between goal patterns were expressed along the block axis (Fig. 1j). The separation along the block axis between blocks 1 and 2, which involved different middle-goal locations, was significantly larger than that between blocks 1 and 3, which shared the same goal configuration (blocks 1–2: 3.73 ± 0.29; blocks 1–3: 1.91 ± 0.28 arbitrary units; p < 0.001 across sessions).

To further assess the segregation of spatial and goal information, we projected CA1 population activity onto either the block axis or the position axes and used linear discriminant analysis (LDA) to quantify how much information about each variable could be recovered (Fig. 1k). Goal information was selectively preserved along the block axis and dropped to near chance along the position axes. Conversely, position information was accurately decoded along the position axes but degraded along the block axis. These results confirm that spatial position and goal state are encoded in largely orthogonal subspaces of CA1 population activity. We reached the same conclusion when LDA was applied directly to the population activity without dPCA projection (Extended Data Fig. 3j).

Together, these findings indicate that CA1 employs orthogonal coding to separate goal blocks while maintaining a stable spatial representation, consistent with the formation of spatially aligned parallel maps (Fig. 1l).

### Pre-navigation CA1 spike sequences distinguish upcoming destinations

If goal-state representations function as episode selectors rather than transient task signals, they should be expressed not only during active navigation but also in the internally generated hippocampal dynamics that precede movement. Although our analyses above establish such representations during active navigation, whether they also emerge during the awake quiescent period preceding navigation onset remains unclear. Hippocampal neurons are known to generate brief spike sequences that recapitulate future or past trajectories^26–29^, but previous studies have largely examined fixed-goal tasks or tasks in which different goals are associated with distinct navigation trajectories. Whether and how goal differences directly shape pre-navigation hippocampal dynamics has therefore remained an open question.

**Fig. 2:**
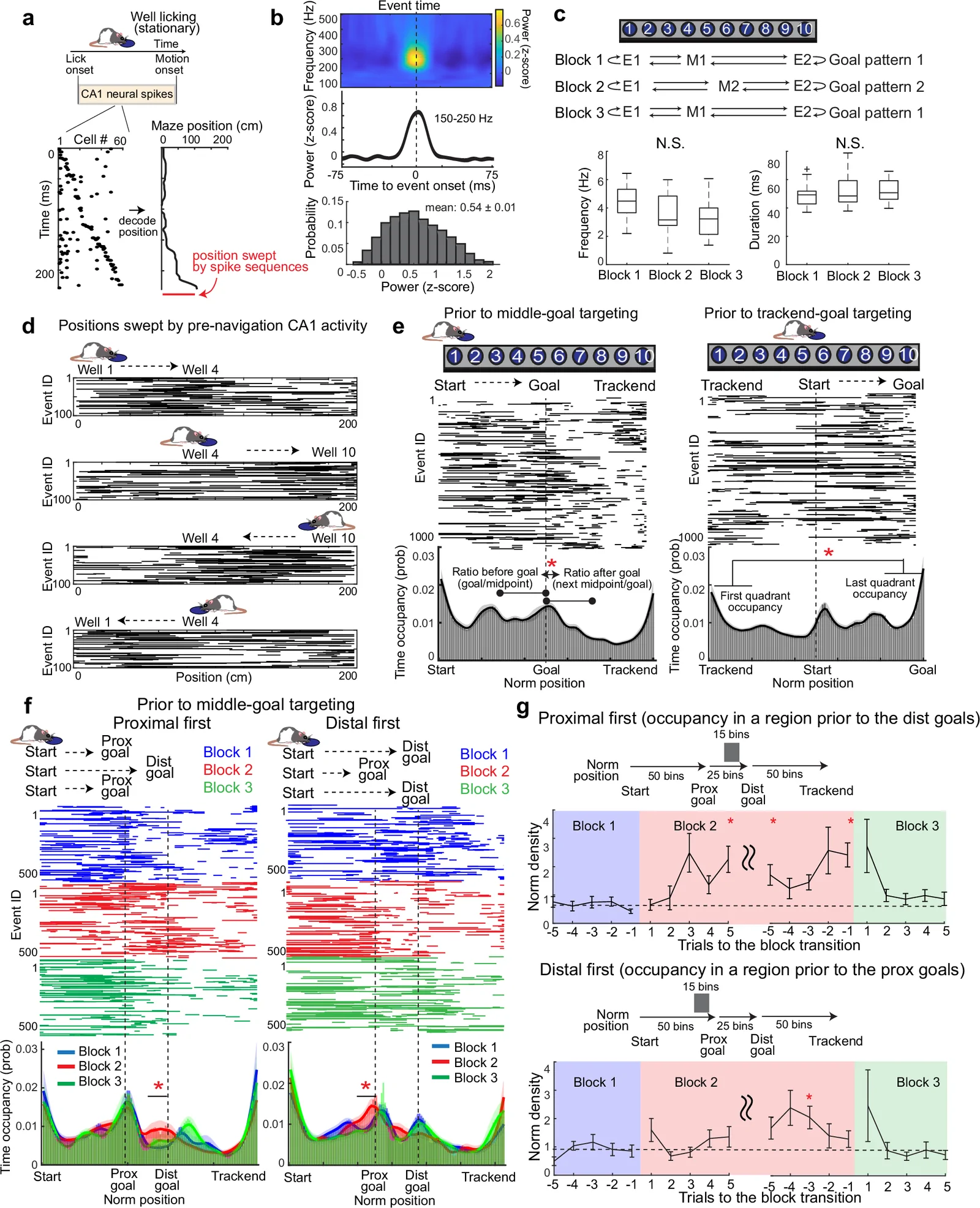
Pre-navigation CA1 spike sequences flexibly adapt to upcoming goal locations. **a** Schematic of pre-navigation spike-sequence detection and definition of the corresponding swept positions. **b** Spectral power in CA1 LFPs averaged around detected spike-sequence events. Top, color-coded spectral power; middle, average power in the 150–250 Hz range; bottom, histogram of z-scored power across events. **c** Frequency and duration of detected spike-sequence events across blocks (*p* > 0.05, *n* = 25, Kruskal–Wallis test). **d** Spatial distributions of individual spike-sequence events along maze positions at different trial phases during quiescence before movement onset. **e** Spatial distributions (top) and time-occupancy probabilities (bottom) across maze positions, shown separately for periods preceding navigation toward middle goals (left) and trackend goals (right). The position axis is normalized per segment: start-to-goal and goal-to-trackend (left) or start-to-trackend (right). Left, the occupancy ratio of the middle goal to the preceding journey midpoint significantly exceeds that of the following midpoint to the middle goal (*p < 0.001, Wilcoxon signed-rank test). Right, time-occupancy is significantly higher in the final quadrant (near the upcoming goal) than the first quadrant (near the previous goal) (*p = 0.007, *n* = 25, Wilcoxon signed-rank test). **f** Spatial distributions (top) and time-occupancy probabilities (bottom) of pre-navigation spike sequences. Left, trials targeting a proximal goal in blocks 1 and 3 and a distal goal in block 2. Right, trials targeting a distal goal in blocks 1 and 3 and a proximal goal in block 2. Black lines mark regions with significantly higher occupancy in block 2 versus blocks 1 and 3 (*p < 0.05, *n* = 25, Wilcoxon signed-rank test). **g** Trial-by-trial time-occupancy probability within the region of interest near the distal goal (top) or proximal goal (bottom). ROIs encompassed the significant points from **f**; these trial-wise statistics descriptively characterize the effect’s temporal evolution. *p < 0.05, n = 25, Wilcoxon signed-rank test relative to block 1.

To examine the spatial content of CA1 pre-navigation activity, we applied our Bayesian position decoder trained on population activity during active locomotion to neural activity recorded during the 3-s period immediately preceding navigation onset, when animals were stationary at the start wells (Fig. 2a). Following previous work^26,45^, we used short decoding windows to capture time-compressed dynamics (20-ms bins shifted every 5 ms; see Methods). A segment of population activity was classified as a pre-navigation sequence event if it met predefined criteria for duration (>20 ms), trajectory smoothness (position jumps between bins <20 cm), and displacement from the animal’s actual position (>25 cm). As previously reported^26,28,29^, these events were predominantly accompanied by sharp-wave ripples in the CA1 local field potential (Fig. 2b).

We first examined the spatial coverage of pre-navigation sequence events. The distribution of represented positions varied markedly with the animal’s current location and upcoming destination (Fig. 2d), indicating that pre-navigation CA1 activity is dynamically modulated by navigational context. Before journeys targeting the middle goals, sequences preferentially represented the segment between the start well and the middle-goal location, with a sharp decline beyond. In contrast, before journeys targeting the trackend goals, sequences showed a modest but significant bias toward future goal locations relative to recently traversed paths (Fig. 2e and Extended Data Fig. 3k).

We next asked how pre-navigation sequences adapted to changes in middle-goal location across trial blocks. Sequence density increased near the newly assigned goal in block 2 relative to block 1 and decreased again when the original goal configuration was reinstated in block 3 (Fig. 2c, f, g). These effects were consistent across sessions and animals (Extended Data Fig. 3k). This goal-dependent modulation emerged even when the animal occupied identical positions, indicating that pre-navigation CA1 activity reflects goal-specific internal dynamics rather than merely spatial or sensory differences.

### Activity in mPFC and NR segregates goal states

Our results thus far show that CA1 population activity encodes both spatial position and goal states along distinct coding dimensions. This organization suggests that goal-related information may be provided to the hippocampus by upstream regions. While the medial entorhinal cortex is a major source of spatial information to the hippocampus^8,51^, how CA1 receives information about different goal configurations during navigation remains unclear. Based on prior evidence that trajectory-related signals are transmitted through the medial prefrontal cortex (mPFC)–nucleus reuniens (NR)–CA1 pathway^17^, we hypothesized that goal-related information in CA1 may depend on this circuit.

**Fig. 3:**
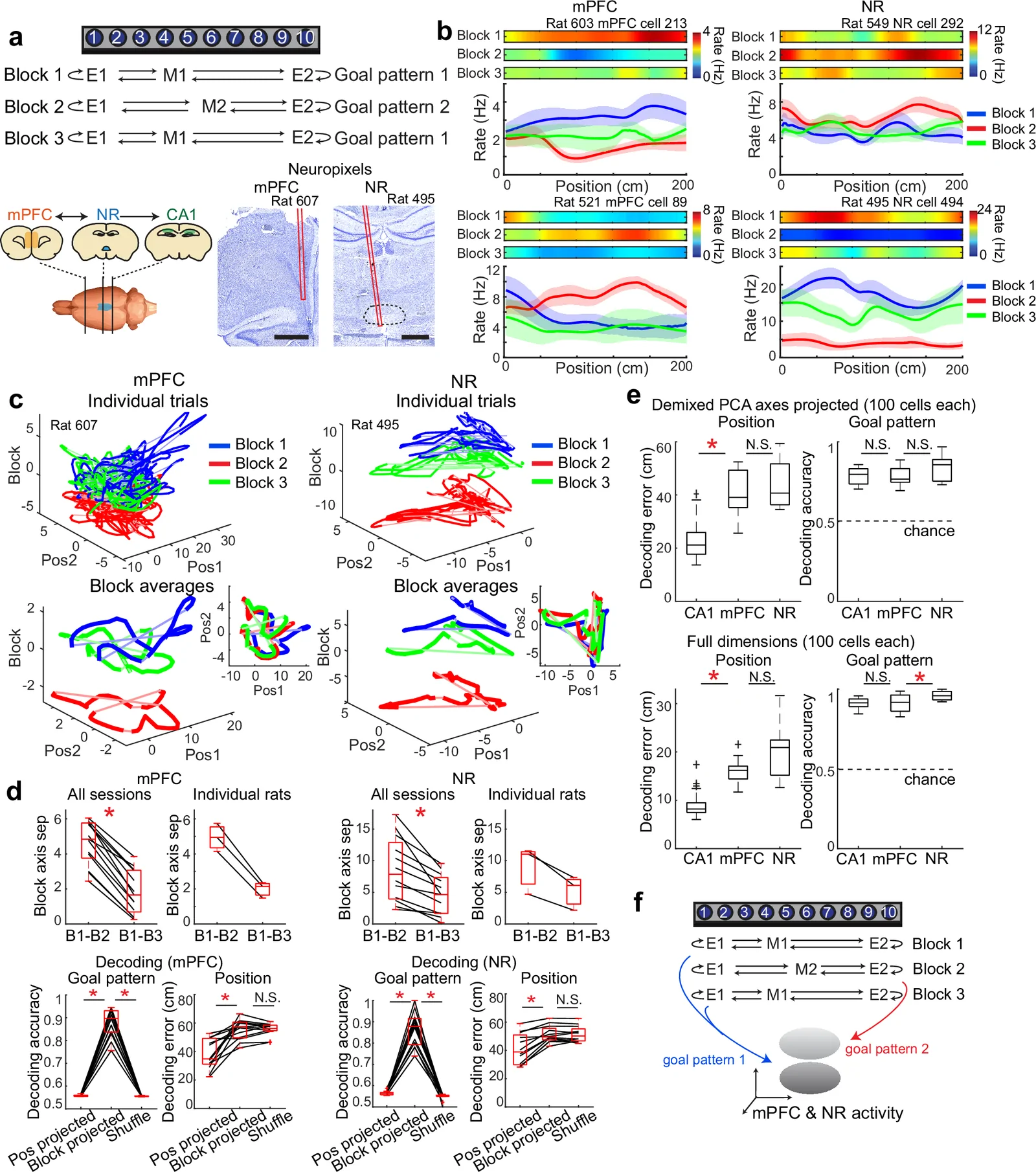
Activity in mPFC and NR segregates task blocks associated with different goals. **a** Top, task structure of three blocks with changes in the middle-goal location. Bottom, schematic of the mPFC–NR–CA1 circuit and Nissl-stained coronal sections showing Neuropixels 1.0 probe tracks in mPFC and NR. Scale bar = 1 mm. **b** Example firing patterns of two representative mPFC and two representative NR neurons. **c** 3D projections of mPFC and NR population activity onto the block component and two position components, as in Fig. 1j. Top, individual trials; bottom, block-averaged activity (upper-right inset: position-component projections). **d** Top, box plots quantifying block-axis separation between blocks 1 and 2 versus blocks 1 and 3, across 13 sessions (mPFC), 12 sessions (NR), and 3 rats; separation is significant in both regions (*p < 0.05, Wilcoxon signed-rank test). Bottom, information content along each projection axis: population activity was projected onto either the block or position components, and goal- or position-decoding performance evaluated accordingly. Shuffle shows performance after label shuffling. *p < 0.05, Wilcoxon signed-rank test. **e** Information content across CA1, mPFC, and NR using matched neuron numbers (100 cells). Decoding was assessed on dPCA-derived orthogonalized axes (top) or full dimensions (bottom). *p < 0.05, Wilcoxon rank-sum test. **f** Schematic of the proposed neural representational structure in mPFC and NR. These regions robustly segregate goal states but show comparatively weak spatial-position coding, consistent with a role in state coding rather than in forming spatial maps.

To test this hypothesis, we recorded neuronal population activity from mPFC and NR using Neuropixels probes while rats performed the same navigation task (mPFC: 708 neurons from 3 animals; NR: 503 neurons from 3 animals; Fig. 3a, b and Extended Data Fig. 4). We first assessed whether the upcoming goal could be decoded from population activity at navigation onset. Decoding accuracy remained at chance levels in both regions (Extended Data Fig. 5d, e), indicating that neither mPFC nor NR robustly encodes the next goal of individual journeys, in contrast to the prospective goal coding previously reported in the orbitofrontal cortex^40^.

We next examined whether population activity in mPFC and NR segregates goal blocks, analogously to CA1. Using dPCA, we decomposed neural responses into block-related and position-related components (explained variance: goal, mPFC 7.2 ± 0.9%, NR 19.6 ± 2.5%; position, mPFC 56.5 ± 5.3%, NR 30.1 ± 3.4%; Fig. 3c and Extended Data Fig. 6a). After orthogonalizing the components, we visualized population trajectories in the low-dimensional space defined by the block and position axes. In both regions, trajectories were more strongly separated along the block axis between blocks with different goal patterns (blocks 1 vs. 2) than between blocks with identical goal patterns (blocks 1 vs. 3) (mPFC: 4.64 ± 0.34 vs. 1.82 ± 0.34; NR: 8.61 ± 1.48 vs. 4.67 ± 0.91; p < 0.001, Wilcoxon signed-rank test). In contrast to CA1, however, population trajectories in mPFC and NR showed less consistent structure along the position axes (Fig. 3c, insets).

To further quantify the information carried along these axes, we projected population activity onto either the block component or the position components and assessed decoding performance using linear discriminant analysis (Fig. 3d). In both mPFC and NR, goal decoding remained high along the block axis (mPFC: 0.88 ± 0.02; NR: 0.86 ± 0.02) but dropped to near chance along the position axes. Position information was decoded above chance from the position axes, but overall position decoding accuracy in mPFC and NR was substantially lower than in CA1 (decoding errors: mPFC 38.4 ± 2.9 cm, NR 40.9 ± 3.1 cm), indicating weaker spatial fidelity in these regions.

To directly compare coding properties across regions, we subsampled neural populations to matched sizes (100 neurons per session). After subsampling, position decoding accuracy remained significantly higher in CA1 than in mPFC or NR, whereas goal-pattern decoding was comparable across all three regions, both for dPCA-projected and full-dimensional population activity (Fig. 3e).

Together, these results show that mPFC and NR encode goal-related information with a representational structure comparable to that observed in CA1, while exhibiting substantially weaker representations of spatial position (Fig. 3f). This shared goal-state structure motivated the hypothesis that NR contributes causally to goal-state coding in CA1.

### mPFC, NR, and CA1 persistently represent goal states during locomotion and immobility

The analyses above demonstrate that mPFC and NR carry goal-state information during active navigation. A prominent feature of CA1 activity, however, is the presence of goal-associated spike sequences that occur during periods of immobility (Fig. 2). This raises the question of whether goal-state representations in the mPFC–NR–CA1 circuit extend beyond locomotion and persist during stationary periods.

**Fig. 4:**
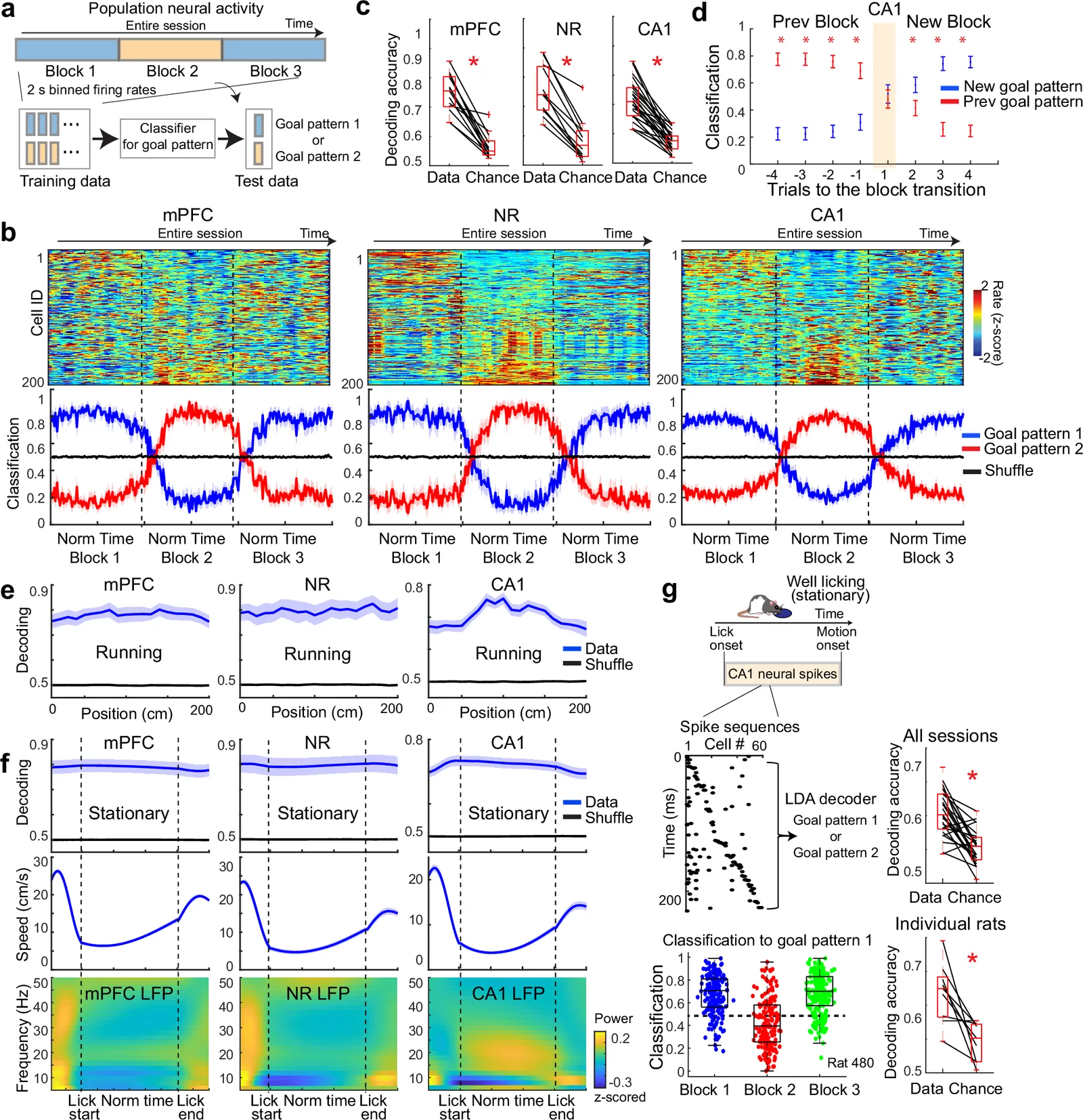
Persistent goal-state coding across the mPFC-NR-CA1 circuit throughout locomotor and stationary states. **a** Schematic of the goal-pattern decoding strategy. Ensemble activity was segmented into 2-s bins; for training, 15 bins were randomly sampled from blocks 1 and 3 and 30 from block 2, and the classifier predicted the goal pattern of withheld test bins. **b** Top, color-coded, z-scored activity of 200 randomly sampled neurons across a full session (blocks 1–3), sorted by firing-rate difference between goal patterns 1 and 2; time axes normalized to each block’s duration. Bottom, goal-pattern classification probabilities on the same axis. **c** Box plots of mean goal-pattern decoding performance from mPFC, NR, and CA1 ensembles versus chance estimated by within-block pseudo-segmentation controls (Extended Data Fig. 7a, b). Performance is significantly above chance in all regions (*p < 0.05, Wilcoxon signed-rank test; mPFC n = 13, NR n = 12, CA1 *n* = 25). **d** Trial-by-trial decoding performance aligned to block transitions, significantly above the theoretical chance level of 0.5 (*p < 0.05, n = 25, Wilcoxon signed-rank test). During the first trial after each transition (shaded), water was pre-delivered at the goal well and sub-maze LEDs illuminated to aid recognition of the new goal configuration. **e** Goal-pattern decoding during the running phase for each region. **f** Goal-pattern decoding during the stationary (well-licking) phase. Despite marked reductions in running speed and LFP theta power, decoding remains stable. **g** Goal-pattern classification from pre-navigation CA1 spike sequences during quiescence, applying the classifier from (**a**) to the spike-sequence events detected as in Fig. 2a. Bottom left, classification for individual events. Right, box plots of decoding performance across all 25 sessions and individual rats. Chance was estimated by within-block pseudo-segmentation controls, as in (**c**). *p < 0.05, Wilcoxon signed-rank test.

To address this question, we applied linear discriminant analysis (LDA) to classify the active goal pattern across entire recording sessions using time-segmented population activity, irrespective of the animal’s behavioral state (Fig. 4a). The decoder was trained on mean firing rates computed from randomly sampled 2-s time segments (equal numbers from blocks 1 and 3, and twice as many from block 2 to balance class labels) and tested on held-out segments (see Methods). Importantly, training segments were selected without regard to the animal’s position, movement, or locomotor state.

Despite this state-agnostic training procedure, the goal configuration was reliably decoded throughout each session in all three regions (Fig. 4b). Decoding accuracy was significantly above chance in mPFC, NR, and CA1 (mPFC 0.747 ± 0.018, NR 0.754 ± 0.025, CA1 0.714 ± 0.013; all p < 0.001, Wilcoxon signed-rank test; mPFC: 13 sessions from 3 animals; NR: 12 sessions from 3 animals; CA1: 25 sessions from 9 animals; Fig. 4c and Extended Data Fig. 7a, b). At block transitions, when the middle-goal location changed, goal-state representations switched within 1–2 trials (4–8 journey segments) across all three regions (Fig. 4d and Extended Data Fig. 7c), indicating rapid and coordinated updating of goal-state representations throughout the circuit.

We next examined whether goal-state decoding performance depended on the animal’s behavioral state. Transitions between maze running and stationary well-licking periods were accompanied by pronounced reductions in running speed and hippocampal theta power. Despite these changes, goal-state decoding performance remained stable across behavioral states in all three regions (Fig. 4e, f). This result indicates that goal-state representations in the mPFC–NR–CA1 circuit are maintained irrespective of locomotor state.

Given that goal-state representations persist during stationary periods, we asked whether these state differences are also reflected in the pre-navigation spike sequences observed in hippocampal CA1 during immobility. To test this, we computed population firing rates during individual pre-navigation spike-sequence events and applied the LDA classifier trained on 2-s segments (Fig. 4g; see Methods). The currently engaged goal state was decoded from pre-navigation sequences significantly above chance (decoding performance: data, 0.604 ± 0.007; chance, 0.553 ± 0.005; *p* < 0.001, Wilcoxon signed-rank test).

Together, these results demonstrate that goal-state information is persistently represented across mPFC, NR, and CA1 throughout task engagement, regardless of whether the animal is locomoting or immobile. This persistent goal-state signal is also expressed in CA1 pre-navigation spike sequences, linking global contextual representations to the transient internal dynamics that precede goal-directed navigation.

### NR silencing reduces goal-dependent CA1 dynamics while sparing spatial coding

**Fig. 5:**
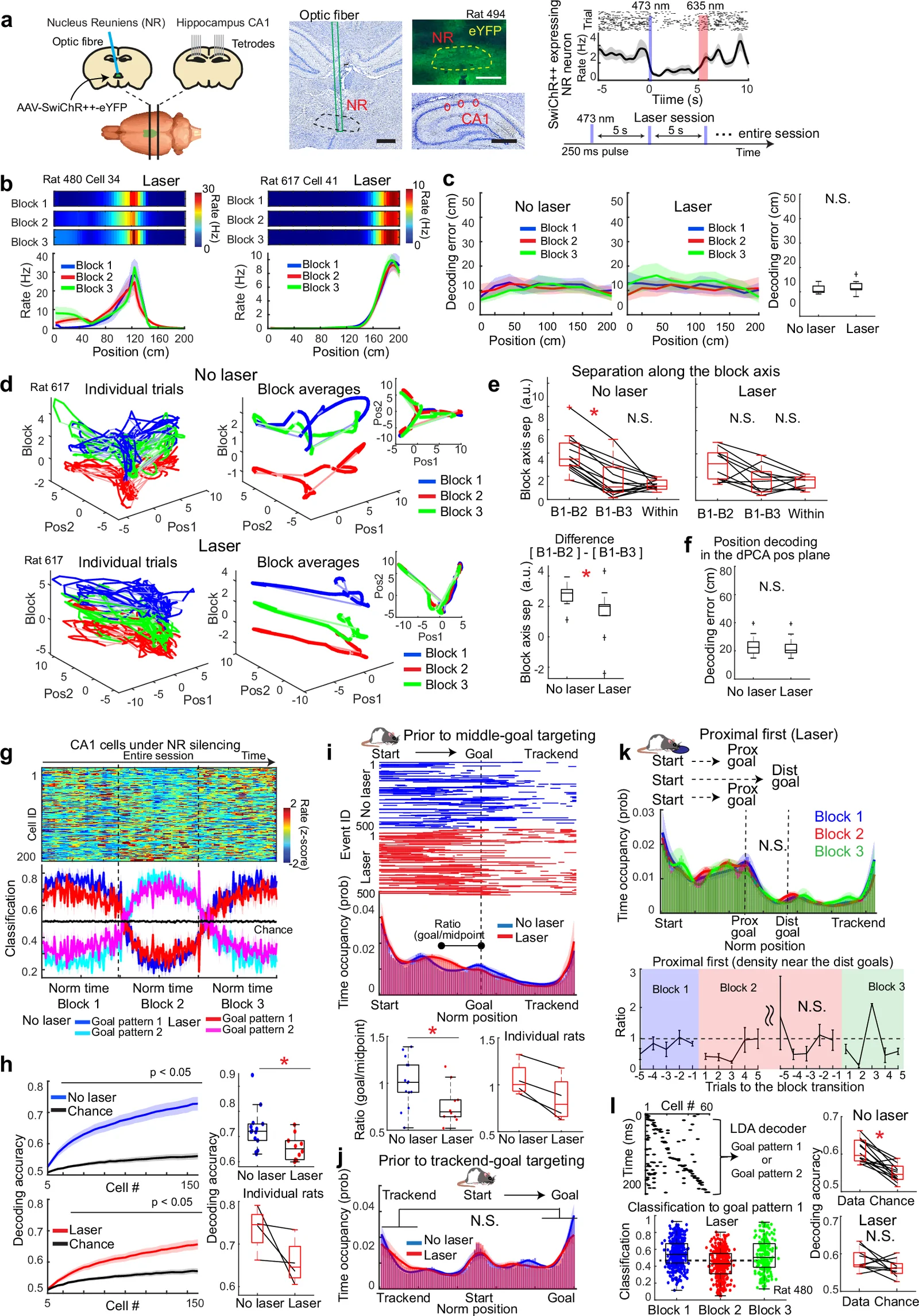
NR silencing diminishes CA1 goal-state coding and pre-navigation prospective activity while sparing spatial coding. **a** Left, surgical schematic. Middle, sections showing the NR optic-fiber track, SwiChR++–eYFP expression, and CA1 tetrode tracks. Scale bar = 1 mm. Right, example SwiChR++ NR neuron showing reduced firing to 473-nm light; protocol: 250-ms pulses at 5-s intervals. **b** Firing-rate maps of two representative CA1 place cells with stable spatial tuning and minimal rate modulation across blocks. **c** Decoding errors across maze positions with and without NR silencing. Box plot (right): no significant difference (*p* > 0.05, Wilcoxon rank-sum test; *n* = 14, 10). **d** CA1 activity projected onto dPCA-derived block and position axes. **e** Block-axis separation (blocks 1–2 vs 1–3) and maximum within-block separation. Block-specific separation is significantly reduced during silencing (no laser p < 0.001; laser *p* = 0.065, Wilcoxon signed-rank test); blocks 1–3 versus within-block is not significant (*p* = 0.463; 0.557). Bottom, direct comparison shows significant reduction with laser (*p* = 0.013). Wilcoxon rank-sum test. **f** Spatial information in dPCA position components; no significant laser difference (*p* > 0.05). **g** Top, z-scored activity of 200 CA1 neurons during silencing. Bottom, goal-pattern decoding from 150 CA1 neurons, laser versus no-laser. **h** Cell-number–matched goal-pattern decoding versus chance (Extended Data Fig. 10e), significantly reduced during silencing (*p* = 0.015, Wilcoxon rank-sum test). **i** Time-occupancy of pre-navigation spike sequences preceding middle-goal trials. The goal-to-preceding-midpoint ratio (Fig. 2e) is significantly reduced with laser. Bottom, sessions (*n* = 14, 10) and 4 rats. *p = 0.038, Wilcoxon rank-sum test; *p* = 0.049, linear mixed-effects model. **j** As in (i), for trackend goals; no significant difference (*p* > 0.05). **k**, As in Fig. 2f, during silencing. Bottom, trial-wise densities relative to block 1. ROI from control data (Fig. 2g), applied unchanged; no significant across-block differences (*p* > 0.05, *n* = 10). **l** Goal-pattern classification from pre-navigation spike sequences, as in Fig. 4g. Bottom left, individual events (rat 480, laser). Right, decoding significantly impaired during silencing (no laser *p* < 0.001, laser *p* = 0.193; chance from within-block pseudo-segmentation).

The observation that goal states are consistently represented across mPFC, NR, and CA1 raises the possibility that NR contributes to goal-state representations in CA1, potentially relaying information from mPFC. To test whether NR activity is necessary for goal-state representations in CA1, we performed optogenetic silencing of NR neurons while recording CA1 activity during task performance. An adeno-associated virus encoding the inhibitory opsin SwiChR++^52^ was injected into NR, and an optic fiber was implanted at the same site (Fig. 5a and Extended Data Fig. 2). The same animals were also implanted with tetrode microdrives targeting dorsal CA1. NR inactivation was achieved by delivering brief 473-nm laser pulses at low frequency (0.2 Hz) throughout the recording session.

We first assessed whether NR silencing affected behavioral performance. Task accuracy and running speed did not differ significantly between sessions with and without laser stimulation (task accuracy: no laser, 89.5 ± 2.1%; laser, 86.5 ± 3.0%; p = 0.519; running speed: no laser, 20.7 ± 1.1 cm/s; laser, 19.2 ± 1.1 cm/s; p = 0.429; Wilcoxon rank-sum tests; Extended Data Fig. 8a), indicating that NR silencing did not impair overall task engagement or locomotion.

We next examined the effects of NR silencing on CA1 neuronal activity, comparing recordings from the same animals across sessions with and without laser stimulation (no laser: 923 neurons; laser: 916 neurons; 4 animals). Classification of CA1 neurons revealed a significant reduction in the proportion of rate-remapping place cells during laser sessions (no laser: 39.1 ± 2.8%; laser: 29.6 ± 2.7%; p = 0.020, Friedman test; Fig. 5b and Extended Data Fig. 9a–f). A similar reduction was observed among non-spatial cells with significant goal-dependent rate changes (no laser: 30.3 ± 1.4%; laser: 21.3 ± 1.8%; p = 0.020). In contrast, the proportion of global-remapping place cells did not differ significantly between conditions. Consistent with these findings, population-vector (PV) correlation analyses showed that under NR silencing, ensemble activity no longer differed between blocks with different versus identical goal configurations, for either spatial or non-spatial cells, in contrast to the clear goal-dependent differences observed in control sessions (Extended Data Fig. 9g).

Importantly, NR silencing did not affect the mean firing rates (no laser: 1.53 ± 0.08 Hz; laser: 1.44 ± 0.07 Hz; p = 0.883), bursting rates (no laser: 0.021 ± 0.003 Hz; laser: 0.015 ± 0.002 Hz; p = 0.060), spatial information content of CA1 place cells (no laser: 0.580 ± 0.014 bits/spike; laser: 0.578 ± 0.013 bits/spike; p = 0.934, Kolmogorov–Smirnov test; Extended Data Fig. 9b, e), or position decoding accuracy from CA1 population activity (no laser: 10.9 ± 0.5 cm; laser: 11.9 ± 0.8 cm; *p* = 0.169; Wilcoxon rank-sum test; Fig. 5c).

To assess how NR silencing altered the structure of CA1 population activity, we applied the same dPCA-based manifold analysis used in Fig. 1i, j. Under NR silencing, the separation between CA1 population trajectories for different goal blocks was significantly reduced along the block axis (Fig. 5d–f and Extended Data Fig. 10a). This reduction was consistent across sessions and animals (goal-axis separation difference between blocks 1 vs. 2 and blocks 1 vs. 3: no laser, 2.81 ± 0.20; laser, 1.64 ± 0.57; *p* = 0.013, Wilcoxon rank-sum test; laser condition × block-pair interaction, *p* = 0.038, repeated-measures ANOVA). The separation between blocks 1 and 3 did not significantly exceed the maximum trial-to-trial separation within individual blocks, confirming that the NR-silencing effect reflects reduced goal-state discrimination rather than a change in the same-goal-block baseline. In contrast, spatial representations along the position axes were unaffected (position decoding error: no laser, 23.5 ± 1.9 cm; laser, 22.9 ± 2.3 cm; *p* = 0.661).

To further quantify the impact of NR silencing on goal-state information, we constructed LDA-based goal-pattern classifiers from randomly selected 2-s population activity segments, irrespective of behavioral state (Fig. 5g). After matching population size across conditions by subsampling, NR silencing significantly reduced goal-pattern decoding accuracy in CA1 (no laser: 72.8 ± 2.2%; laser: 65.6 ± 1.6% with 150 randomly sampled neurons; p = 0.015, Wilcoxon rank-sum test; Fig. 5h and Extended Data Fig. 10e). This effect was confirmed by a linear mixed-effects model (condition as a fixed effect, animal as a random intercept), with significance assessed via a permutation test in which condition labels were shuffled within each animal (p = 0.013).

Finally, we examined whether NR silencing affected pre-navigation CA1 spike sequences, which normally exhibit goal-dependent modulation (Fig. 2). NR inactivation did not significantly alter the frequency or duration of detected spike-sequence events (no laser: 3.71 ± 0.34 Hz, 51.8 ± 2.0 ms; laser: 3.58 ± 0.34 Hz, 55.1 ± 2.9 ms; p > 0.05, Wilcoxon rank-sum test; Extended Data Fig. 8c). However, prior to middle-goal–directed journeys, NR silencing significantly reduced the persistence of pre-navigation sequences toward the goal location (Fig. 5i and Extended Data Fig. 10g). In contrast, the spatial distribution of pre-navigation sequences preceding trackend–goal–directed journeys was largely unaffected (Fig. 5j). Moreover, under NR silencing, pre-navigation sequence activity did not show a statistically significant block-dependent adaptation to middle-goal locations (Fig. 5k and Extended Data Fig. 10h), indicating that the impact of NR silencing on goal-directed sequences is specific to target wells that change between blocks.

Consistent with these observations, classification of individual pre-navigation spike-sequence events by goal pattern was not significantly above the estimated chance level during NR silencing, whereas significant classification was observed in control sessions (decoding performance: no laser 0.608 ± 0.008; laser 0.584 ± 0.009; chance 0.552 ± 0.007 and 0.559 ± 0.008, respectively; p < 0.001 no laser vs. chance, p = 0.193 laser vs. chance, Wilcoxon signed-rank test; Fig. 5l). Together, these results indicate that NR activity supports the robust expression of goal-state-dependent modulation in CA1 population activity and pre-navigation dynamics, while leaving CA1 spatial coding largely intact.

## Discussion

In this study, we show that population activity in mPFC, NR, and CA1 carries goal-state representations that distinguish navigation episodes within the same spatial context, and that inactivation of NR significantly reduces the CA1 goal-state signal. These results identify NR as a pivotal node in the circuit supporting this hippocampal representation. CA1 implements goal-state coding through an orthogonal scheme in which spatial position and goal-state information occupy largely independent dimensions of the population activity manifold. This organization allows goal-state signals to be incorporated without disrupting positional coding, thereby minimizing representational interference between episodes formed in the same spatial context. Importantly, goal-state signals persist across both locomotor and stationary phases, enabling the hippocampus to engage a goal-state-specific map not only during navigation but also during planning before movement begins.

Previous studies have implicated the prefrontal cortex in representing navigational goals or intended destinations^36,40^, raising the possibility that prefrontal–hippocampal interactions shape goal-dependent hippocampal activity. Here, however, we find that mPFC and NR do not encode the immediate next goal of individual journeys (Extended Data Fig. 5d,e). Instead, these regions form persistent neural states that reflect the blockwise goal configuration. We thus use the term “goal state” to refer to the blockwise configuration of rewarded locations, not the next destination within a journey. These states are maintained across locomotion and immobility and remain robust despite trial-by-trial behavioral variability, suggesting that they provide a stable contextual signal that distinguishes goal-configuration–specific episodes over extended timescales. This contextual signal is represented in mPFC and NR and depends on NR activity for its robust expression in CA1, where it selectively modulates the goal-coding dimension of the population manifold. Consistent with this model, inactivation of NR reduced CA1 goal-state coding, the separation between goal-state-specific CA1 manifolds, and goal-dependent prospective activity in pre-navigation CA1 spike sequences, indicating that NR supports the robust expression of this CA1 goal-state dimension. This selective dissociation—attenuated goal-state coding with spared spatial representations—suggests that NR-dependent goal-state activity does not reshape the hippocampal spatial map itself, but rather modulates goal-state-specific activity within an otherwise stable spatial framework.

Despite this marked reduction in CA1 goal-state coding, NR silencing produced no apparent behavioral deficit in the present linear maze task. Prior work using the same maze, albeit with variations in task rules, has shown that performance depends on the orbitofrontal cortex^40^ and on dopamine release in the nucleus accumbens^53^, whereas hippocampal lesions leave learning and performance intact, consistent with the view that the hippocampus is dispensable for this task^54^. NR-dependent goal-state activity in CA1 described here therefore likely contributes to functions beyond simple goal targeting. We propose that it supports mental simulation or prospective planning of upcoming trajectories, a role that may become especially prominent in more complex spatial environments.

The CA1 goal-state representations described here can be viewed as an instance of the broader context-coding schemes reported previously. Trajectory-dependent “splitter cells” were originally described in trajectory-choice tasks^17–20^, whereas rate remapping—whereby place cells alter their firing rates across contexts while preserving spatial tuning—was initially described in response to changes in the sensory context of an environment^10^. Two theoretical accounts have since been advanced to explain such trajectory- and context-dependent firing: temporal-context models, in which a decaying trace of recent experience is incorporated into place-cell activity and predicts temporally graded coding, and latent-state inference, in which experience is assigned to discrete internally inferred states that need not preserve spatial or temporal structure^49^. These frameworks make distinct, testable predictions about the geometry of population activity, which we used here to characterize the CA1 goal-state signal. Our findings are more consistent with a latent-state account and further define the geometry and expression of this signal. First, the goal-state code occupies a population dimension largely distinct from the spatial-position axes and lacks the continuous spatial organization of the position code, as expected for a discrete state representation. Second, goal-state decoding remained largely stable across maze position, arguing against a simple position-dependent temporal-context gradient. Third, the goal-state signal persists during stationary and quiescent periods and is expressed in pre-navigation spike sequences, demonstrating that it is not restricted to active locomotion. This behavioral-state independence is notable given the strong locomotion dependence of most hippocampal activity, including large changes in firing rate and LFP theta power^32–35^. Consistent with state-independent expression, putative CA1 interneurons, which fire across a wide range of behavioral states, also carried goal-state information (Extended Data Fig. 3g, 7f,g). Finally, silencing NR selectively reduced coding along the block axis while sparing the spatial representation, identifying NR as a causal contributor to the CA1 goal-state signal. Together, these findings characterize the CA1 goal-state representation as a discrete latent-state-like code that occupies a representational dimension distinct from the spatial coding manifold.

This persistence of goal-state representations offers a mechanistic explanation for how the hippocampus can generate goal-dependent pre-navigation spike sequences. Because goal-specific CA1 population states are already engaged during immobility, the hippocampus can express prospective activity biased toward the intended destination before movement onset. This framework helps reconcile diverse observations regarding hippocampal pre-navigation spike sequences, which have been reported to represent future, past, or alternative trajectories depending on task demands^26,27,55^. Such variability may reflect top-down control over which goal-state-specific map is selected at a given moment, biasing the generation of behaviorally relevant sequences, as proposed in several theoretical models^56,57^.

Although goal configuration was the experimentally controlled variable in the present study, the broader computational problem extends beyond goals. Animals repeatedly encounter the same environment under different task demands, motivational states, and intended actions, requiring memory systems to distinguish overlapping behavioral episodes without disrupting the underlying spatial map. Our results reveal one implementation of this principle: goal-defined navigation episodes can be separated along a non-spatial population dimension while preserving the spatial organization of CA1 activity. More broadly, this mechanism offers a circuit-level solution to a problem central to episodic memory, cognitive control, and adaptive behavior across species: how memory systems achieve flexible retrieval and planning in stable environments^2,58–61^. Our findings suggest that the hippocampus can resolve this challenge by constructing parallel spatially aligned maps, each indexing a distinct goal-defined behavioral episode through largely orthogonal coding of spatial and non-spatial information. A key computational consequence of this architecture is that previously established maps can be preserved rather than overwritten, as new experiences accumulate. When a previously encountered goal pattern reappeared after an intervening block, the corresponding CA1 population representation was selectively reinstated, indicating that CA1 maintains multiple goal-state-specific maps within the same environment. This reinstatement is a defining feature of a memory system and is distinct from the gradual temporal drift commonly reported in cortical and hippocampal populations^62–64^. Consistent with this distinction, goal-state coding was consistently stronger than the discrimination of arbitrarily defined time segments within the same block (Extended Data Fig. 7a, b), and although CA1 activity also carries block-sequence information, this information resides in a subspace largely orthogonal to the goal-state coding axis (Extended Data Fig. 10i–k). This geometry allows the hippocampus to retain stable representations of shared structure while flexibly indexing individual episodes.

In this framework, goal-state representations in mPFC and NR may act as a selection signal that biases which spatial map is engaged. This interpretation aligns with the proposed roles of the prefrontal cortex^65–67^ and nucleus reuniens^68–71^ in memory encoding, retrieval, and consolidation. For example, NR inactivation impairs the encoding and retrieval of contextual fear memories^71^ and of trajectory-choice encoding in a spatial working memory task^70^, implicating NR in hippocampus-dependent coding of contextual states. The present study provides a circuit-level account of how NR activity shapes hippocampal coding: we identify goal-state-specific neural representations across the mPFC–NR–CA1 circuit and show that this state coding persists into the stationary phase and modulates prospective coding in the hippocampus, moving beyond the encoding/retrieval framework examined in prior work. Notably, this locomotion-independent goal-state signal in mPFC and NR allows the hippocampus to sustain a consistent internal state that links task-specific activity across behavioral phases, from rate remapping during locomotion to pre-navigation spike sequences during immobility. By identifying prefrontal–thalamic contributions to the selection of spatially aligned hippocampal maps, our findings bridge circuit-level interactions with the population-coding principles that support flexible memory access, planning, and goal-directed behavior.

## Methods

### Subjects

All experiments were approved by the local authorities (RP Darmstadt, protocols F126/1009, F126/1026, and F126/2005) in accordance with the European Convention for the Protection of Vertebrate Animals used for Experimental and Other Scientific Purposes. Subjects were fourteen male Long-Evans rats weighing 400 to 550 g (aged 3–5 months) at the start of the experiment. Rats were housed individually in Plexiglass cages (45 × 35 × 40 cm; Tecniplast GR1800) and maintained on a reversed 12-h light-dark cycle, with behavioral experiments performed during the dark phase. Animals were mildly water-restricted with unlimited access to food and kept at 85–90% of their free-feeding body weight throughout the experiment. Nine rats were implanted with tetrode drives bilaterally in the dorsal CA1 of the hippocampus. Four of them additionally received injections of AAV2/1-CaMKIIα-SwiChR++-eYFP into the nucleus reuniens (NR) of the thalamus, where optic fibers were also implanted. Five rats were implanted with Neuropixels 1.0 probes: one with 2 probes in NR, one with 1 probe in NR, two animals with 1 probe in mPFC, and another with 1 probe in mPFC and 2 probes in NR.

### Surgery, virus injection, and drive implantation

Anesthesia was induced by isoflurane (5% induction concentration, 0.5–2% maintenance adjusted according to physiological monitoring). For analgesia, Buprenovet (Buprenorphine, 0.06 mg/mL; WdT) was administered by subcutaneous injection, followed by local intracutaneous application of either Bupivacaine (Bupivacaine hydrochloride, 0.5 mg/mL; Jenapharm) or Ropivacaine (Ropivacaine hydrochloride, 2 mg/mL; Fresenius Kabi) into the scalp. Rats were subsequently placed in a Kopf stereotaxic frame, and an incision was made in the scalp to expose the skull. After horizontal alignment, several holes were drilled into the skull to place anchor screws, and craniotomies were made for microdrive implantation. The microdrive was fixed to the anchor screws with dental cement, while two screws above the cerebellum were connected to the ground of the recording devices. All tetrodes were then positioned at a depth of 750 μm from the cortical surface. All animals received analgesics (Metacam, 2 mg/mL Meloxicam; Boehringer Ingelheim) and antibiotics (Baytril, 25 mg/mL Enrofloxacin; Bayer) for at least 5 d post-surgery.

For tetrode recordings, rats were implanted with a microdrive that contained 28 individually adjustable tetrodes made from 17 μm polyimide-coated platinum-iridium (90–10%; California Fine Wire; plated with gold to impedances below 150 kΩ at 1 kHz). The tetrode bundle consisted of 30-gauge stainless steel cannulas, soldered together in 14 × 2 circular shapes. Tetrode drives were implanted within the dorsal CA1 region of the hippocampus bilaterally, with coordinates at AP: −4.0 mm, ML: 3.5 mm. The tetrodes were adjusted to depths of 1.8–2.2 mm DV. Concurrently, four of these rats also received injections of AAV2/1-CaMKIIα-SwiChR++-eYFP (a gift from Dr Karl Deisseroth)^52^ into NR at four sites in each hemisphere, with each site receiving 250 nl (a total of 1000 nl per hemisphere). The injections were administered at a rate of 100 nl/min with coordinates of AP: −2.0 mm and −2.5 mm, ML 0.6 mm, and DV 6.5 and 6.75 with an angle of 4° toward the midline. After each injection, there was a 10-minute waiting period before proceeding with the next injection site. An optic fiber (Lambda fiber, OptogeniX) was implanted into the NR at the coordinates of AP: −2.25 mm, ML 0.6 mm, and DV: 6.75 mm with an angle of 4° toward the midline. After the surgery, the animals were allowed to recover for 5-7 days before starting recordings. Optogenetic experiments were conducted at least 4 weeks after surgery to allow for opsin expression.

In addition, one rat was implanted with a Neuropixels 1.0 probe in NR with coordinates of AP − 2.5 mm, ML 0.6 mm, and DV: 7.5 with an angle of 5° to the midline. Another subject was implanted with two Neuropixels probes in NR bilaterally with the aforementioned coordinates. One rat had three Neuropixels 1.0 probes: two in NR bilaterally and another in the mPFC with the coordinates of AP 3.25 mm, ML 0.6 mm, and DV 5.0 mm with an angle of 4 degrees to the midline. Two rats were implanted with one Neuropixels 1.0 probe in the mPFC.

### Histological procedures

Once the experiments were completed, the animals were deeply anesthetized with sodium pentobarbital and perfused intracardially with saline, followed by 10% formalin. The brains were extracted and fixed in formalin for at least 72 h at 4 °C. Frozen coronal sections were cut (30 µm), stained with cresyl violet, and mounted on glass slides. To confirm the implanted locations of the tetrodes, optic fibers, or Neuropixels probes, coronal sections at the implanted locations were directly mounted on glass slides from the cryostat, stained with cresyl violet, and covered with coverslips using mounting solution (HISTOMOUNT, Invitrogen, 008030).

To confirm AAV-mediated protein expression, sections were immunostained to enhance the fluorescence signals of SwiChR++-eYFP. The sections were first placed in a blocking buffer (4% [v/v] normal goat serum and 2% [w/v] bovine serum albumin in pH 7.6 TBST) for 30 min. The staining of eYFP was performed with an antibody against GFP (anti-GFP Chicken IgY, 1:1000 dilution, Invitrogen, A10262) together with a corresponding secondary antibody (goat anti-chicken IgY AF594, 1:500 dilution, Invitrogen, A32759). All the images were acquired using a slide scanner (Zeiss Axio Scan.Z1).

### Behavioral task

Rats were acclimated to a 2-m-long linear maze equipped with ten equally spaced water-delivery wells (20 cm apart). The training regimen consisted of three phases. Initially, rats were familiarized with the maze by having access to 100 µl liquid rewards (0.3% saccharin) positioned at both ends of the maze. The wells were prefilled with reward, facilitating the animals’ learning to alternate between the trackend wells over the first one to two days. In the second phase, a middle well was introduced to the goal-well sequence. Water was pre-delivered in the designated wells to facilitate the animal’s learning of a three-well navigation sequence. This training phase took 1–2 days. Finally, a time delay between an animal’s well licking and the reward delivery was introduced. The duration of this delay was fixed throughout each behavioral session, but gradually increased over a period of 7 to 14 days until it reached a range of 0.8 to 1.5 s. Once rats achieved more than 80% accuracy with a delay of at least 0.5 s, the middle well in the sequence was altered to a new location after several trial repetitions. To signal this transition, water was pre-delivered to the new goal well, and LEDs underneath all wells in the maze were turned on until the completion of the first cycle of new goal sequence trials (comprising four journey segments). All experiments were performed under minimal-light conditions (no light source in the recording room, with only weak ambient light from the computer monitors in the adjacent room), and the animal’s position and direction were monitored by two-colored LEDs on the headstage.

### Optogenetic inactivation

Optogenetic experimentation commenced after three weeks of these recording sessions (approximately four weeks post-surgery). During these experiments, 473 nm laser pulses of 250 ms duration were emitted at a frequency of 0.2 Hz using a DPSS laser (Shanghai Laser & Optics Century Co., Ltd.). The laser power at the fiber tip was adjusted to 20 mW. These pulses were applied throughout the recording session. After the session, 635 nm light from a diode laser (Shanghai Laser & Optics Century Co., Ltd) was applied for 10 s to facilitate the recovery from SwiChR++-dependent inactivation.

### Recording procedures and spike sorting

All data processing and analyses were performed with MATLAB (MathWorks). Neural signals from tetrode recordings were acquired and amplified using 64-channel headstages (RHD2164; Intan Technologies), combined with the OpenEphys acquisition system at a sampling rate of 15 kHz. The signals were band-pass filtered at 300–6000 Hz, and spikes were detected and assigned to separate clusters using Kilosort^72^ (https://github.com/cortex-lab/KiloSort) with parameters set to the spike threshold at −4 and the number of filters at 2× the total channel number. Each channel was independently grouped with ‘kcoords’ parameters, and the noise parameter determining the fraction of noise templates spanning all channel groups was set to 0.01. For Neuropixels probe recordings, the signals, high-pass filtered at 300 Hz, were collected with the National Instruments PXIe System and OpenEphysGUI software. Spikes were detected and assigned to separate clusters using Kilosort 3.0 (https://github.com/MouseLand/Kilosort). The resulting clusters were manually reviewed using autocorrelograms and waveform characteristics in principal-component space. Well-isolated single units were retained, whereas multiunit activity and noise clusters were discarded. Neurons with mean firing rates less than 0.1 Hz were excluded.

Neuropixels probe tracks were reconstructed from Nissl-stained sections. Units were assigned to anatomical regions based on the probe channel on which the spike waveform amplitude was maximal. For NR recordings, analyses were restricted to units whose maximal-amplitude channel fell within the 150 channels closest to the probe tip and within the histologically identified NR region; units assigned to channels dorsal to this region were excluded. For mPFC recordings, all channels located within histologically verified anterior cingulate cortex, prelimbic, or infralimbic regions were included.

### Firing properties of individual neurons

To assess the spatial tuning properties of CA1 neurons during navigation, we first defined the beginning of individual journeys using the criteria that running speed exceeded 10 cm/s and the animal was more than 10 cm from the start well. The end of the journey was defined as the time of the first lick detection at the goal well. Error trials and transition trials (the first cycle with new goal introduction) were excluded. We considered firing rates during these defined navigation phases from the beginning to the end of journeys. To avoid counting the same cells multiple times, we used only one recording session from each animal. Spike times of individual CA1 neurons were converted into firing rates by convolving a Gaussian kernel with a bandwidth of 250 ms. We then computed the spatial information content of each neuron’s firing pattern. The spatial information metric quantifies how well spikes of a neuron predict the animal’s location in the environment. We adopted the formula provided by Skaggs et al.^73^, which accounts for the variability in firing rate across different spatial bins and the occupancy probability of each bin, as shown in the following formula:

$${Spatial}\,{Information}={\sum }_{i=1}^{N}{p}_{i}\frac{{\lambda }_{i}}{\lambda }{\log }_{2}\frac{{\lambda }_{i}}{\lambda }$$

Here, ${p}_{i}$ is the occupancy probability of the ith bin, ${\lambda }_{i}$ is the mean firing rate at the i-th bin, λ is the overall mean firing rate, and N is the number of bins. All neurons that exhibited the maximum firing rates above 1 Hz across the two blocks with the same goal combinations (i.e., blocks 1 and 3 in Fig. 1a) were considered. Among them, neurons that exhibited spatial information > 0.25 bits/spike in both blocks were considered spatial cells, and the rest were considered non-spatial cells.

Spatial cells were further categorized based on the stability of spatial tuning. The significance of spatial tuning differences was assessed based on spatial correlations between 1-dimensional firing rate maps along the linear maze obtained from two blocks for each neuron. By shifting the relative positions of these rate maps along the maze positions, we obtained the distributions of spatial correlations across different spatial alignments of the two rate maps. If the 95th percentile of this distribution was achieved within a 10 cm shift of the original data, these two maps were considered to have stable spatial tuning between blocks. Spatial cells with stable spatial tuning between two same-goal blocks were considered place cells.

Place cells were further classified based on their remapping features. Global-remapping cells were defined as neurons that exhibited significant field shifts between blocks with different goal combinations (i.e., blocks 1 and 2) by using the same spatial correlation criteria described in the previous paragraph. For the remaining place cells excluding global-remapping cells, we assessed the overall rate changes by using two-way ANOVA with goal patterns and spatial positions as independent variables. Neurons that showed p < 0.05 in the main effect of goal pattern in the ANOVA and exhibited larger rate differences between different-goal blocks (blocks 1 and 2) than between same-goal blocks (blocks 1 and 3) were considered rate-remapping place cells.

The same criteria for rate remapping were applied to non-spatial CA1 cells. Non-spatial neurons that exhibited p < 0.05 in the main effect of goal pattern in the ANOVA and exhibited larger mean rate differences between different-goal blocks than between same-goal blocks were classified as non-spatial rate-remapping cells.

The center of mass (COM) of firing fields in the maze was calculated for individual neurons using the following formula:

$${COM}=\frac{{\sum }_{i=1}^{N}{\lambda }_{i}\times {{{\rm{pos}}}}_{i}}{{\sum }_{i=1}^{N}{\lambda }_{i}}$$

Here, ${\lambda }_{i}$ is the mean firing rate at the i-th bin, ${{pos}}_{i}$ is the spatial position of the i-th bin, and N is the number of bins. Changes in spatial tuning were assessed by computing the absolute differences in the COMs between blocks. The rate changes between blocks were defined as the absolute difference between the peak firing rates in the two blocks, normalized by their maximum. The comparison of the distributions was performed using the Kolmogorov-Smirnov test.

To assess whether NR silencing altered CA1 bursting, bursts were detected from spike trains of individual CA1 place cells using an inter-spike interval criterion. A burst was defined as a sequence of at least three spikes in which all consecutive inter-spike intervals were ≤6 ms. Burst rate was calculated as the number of detected bursts divided by the total recording duration. Spike rate was calculated as the total number of spikes divided by the recording duration.

### Population vector and statistical analysis

Previous studies have shown that, even when individual neurons maintain the same spatial tuning, spatial codes at a neural population level can differ by changing the distribution of firing rates among neurons (i.e., rate remapping)^10^. To evaluate this effect, we performed a population vector (PV) correlation analysis on all place cells, excluding global-remapping cells. The z-scored firing rates of neurons were calculated for individual 2 cm position bins. Correlations of population firing rates across position bins between different blocks were calculated. Statistical analyses were performed on the PV correlations at the same positions. We used only one recording session per animal, but datasets from two different running directions were treated as repeated measures from the same animals.

### Position decoding

To estimate the spatial position represented by CA1 neurons, we applied a Bayesian decoding approach with a uniform prior^26,45^. This approach requires constructing spatial rate maps for individual neurons. Spike times of individual CA1 neurons were converted into firing rates by convolving a Gaussian kernel with a bandwidth of 250 ms, and the mean firing rates in individual 2 cm bins were estimated across the times when the animal occupied these bins. The times when the animal’s running speed was <10 cm/s were excluded. We constructed separate maps for the two running directions in the linear maze. By using these rate maps, position decoding was performed from the population activity of CA1 neurons. Assuming that neurons exhibit Poisson firing statistics, the likelihood of observing a spike rate vector at individual positions and directions is given by:

$$\Pr \left({pos},\,{dir}|{spikes}\right)=C\left(\mathop{\prod }_{i=1}^{N}{{f}_{i}\left({pos},{dir}\right)}^{{n}_{i}}\right)\exp \left(-\tau {\sum }_{i=1}^{N}{f}_{i}({pos},\,{dir})\right)$$

where C is a normalization constant, τ is the time bin width, ${f}_{i}\left({pos},{dir}\right)$ is the mean firing rate of a neuron i at a given position and direction, ${n}_{i}$ is the observed spike count of the neuron i, and N is the total number of neurons. A 250 ms time window was used to estimate the animal’s position on a behavioral timescale. The maximum likelihood of position and direction was estimated from the neural population activity in individual time bins.

To minimize decoder overfitting, we assessed the decoder’s performance using a ten-fold cross-validation approach. Briefly, we extracted 10% of the data as a test dataset, and the remaining data were used for training to construct rate maps. This procedure was repeated until every fold had served once as the test set. The decoding performance was then estimated by calculating the position decoding errors and the coefficient of determination (R^2^) for both position and direction estimates relative to those obtained from a video camera.

To estimate the impact of goal changes on the position decoding performance, we used two decoding approaches. In the first approach, the data were combined across blocks to train a decoder, and its performance was estimated on test datasets from individual blocks (general decoder). In the second approach, decoders were constructed individually for each block of data, and their performance was evaluated on test datasets from the same blocks (blockwise decoder). We found that the decoding performance was not significantly different between these two strategies nor between blocks (Extended Data Fig. 3b–d).

### Pre-navigation spike sequence detection

To detect pre-navigation sequential spiking events, we applied the same Bayesian decoder used for position estimation on a behavioral timescale. However, we restricted position decoding to the animal’s stationary times before navigation, focusing on 3-s periods before leaving start wells with running speed below 10 cm/s. We applied a moving time window of 20 ms, shifting every 5 ms, to estimate positions. The decoded positions were then assessed using the following three criteria. First, consecutive decoded positions should not exhibit a sudden jump of more than 20 cm. Second, the duration of sequences must exceed 20 ms. Finally, the decoded sequences should move at least 25 cm away from the animal’s position. For analyses combining sessions with different goal locations, sequence occupancy was first computed in 2.5 cm bins along the physical track and then linearly interpolated onto normalized spatial axes. For middle-goal analyses, the start-to-middle-goal segment and middle-goal-to-track-end segment were normalized separately, allowing occupancy to be aligned to the middle-goal location across sessions. To compute the occupancy ratios of pre-navigation sequences in Figs. 2e and 5i, additional references were defined at the midpoints of the middle-goal-directed and subsequent journeys. The occupancy-ratio metric in Fig. 5i was motivated by the spatial pattern observed in Fig. 2e. The middle-goal and journey-midpoint reference points were determined from task geometry and applied identically to laser and no-laser sessions. For comparisons between proximal and distal middle-goal configurations, each trial was normalized into three segments: 50 bins from the start well to the proximal goal, 25 bins from the proximal to the distal goal, and 50 bins from the distal goal to the track end. The regions of interest in Fig. 2g and Fig. 5k were defined as the contiguous bins showing significant blockwise differences in Fig. 2f, and trial-wise densities were calculated by averaging occupancy across these regions.

### Dimensionality reduction using demixed principal component analysis

To characterize the structure of neural population dynamics jointly encoding goal configurations and spatial positions, we applied demixed principal component analysis (dPCA)^50^. Neural activity was organized by neuron identity, maze position, trial block, and trial. Maze positions were discretized into 20 spatial bins for each running direction. To minimize the influence of behavioral uncertainty during goal adaptation, neural activity from the first three cycles following each change in goal configuration was excluded from the analysis. For dPCA, firing rates were averaged across trials within each position bin and goal-configuration condition. Blocks 1 and 3, which shared the same goal configuration, were pooled and contrasted with block 2, which contained the alternative goal configuration. dPCA was then used to decompose population activity into components associated primarily with spatial position and block identity. A regularization parameter of 10^−4^ was used to stabilize component estimation and reduce sensitivity to noise in low-variance dimensions. From the resulting components, we selected the first block component and the first two position components for further analysis. Because dPCA components are not guaranteed to be mutually orthogonal, we orthogonalized these three components using a Gram–Schmidt procedure, removing projections of the block component from the position components. Population activity from individual trials was subsequently projected onto the orthogonalized components, yielding a low-dimensional representation that captured the dominant position- and block-related variance. These components were visualized in three-dimensional space to illustrate the trajectories of neural population activity across different block and spatial conditions.

### LDA-based goal-pattern and position decoding

To quantify spatial and goal-state information across the mPFC–NR–CA1 circuit without assuming spatial tuning of individual neurons, we applied an LDA-based decoding approach to estimate the animal’s position. Neural data were divided into training and test sets using 10-fold cross-validation. Firing rates were estimated by convolving spike trains with a Gaussian kernel (bandwidth 250 ms) and were z-scored across the session.

We constructed LDA decoders from ensemble neural activity corresponding to 20 spatial bins for each running direction along the linear maze, separately for each trial block. Using the withheld test data, we first assessed whether goal patterns could be decoded from population activity. We then constructed an LDA decoder for spatial position, yielding 39 (40 − 1) projection axes, and evaluated decoding performance across the full dimensionality on the test dataset. To generate orthogonal decoders for goal and position, we first reduced the dimensionality of the position decoder by retaining components explaining 80% of the variance. From this position-coding subspace, the component of the goal-coding axis was subtracted, resulting in a position subspace orthogonal to goal coding. All LDA decoders were implemented using the MATLAB fitcdiscr function with a regularization parameter gamma of 0.1, and decoding probabilities were obtained using the predict function on the test dataset. Chance levels were estimated by class-label shuffling.

### Behavior-state-agnostic goal-pattern decoding

To decode goal patterns associated with individual task blocks across behavioral states, we applied linear discriminant analysis (LDA) to population neural activity recorded throughout the session, including both running and immobility periods. The data were segmented into 2-s windows. To construct the goal-pattern decoder, neural activity from the first three cycles following the introduction of a new goal configuration was excluded to avoid contamination by transitional uncertainty during goal adaptation. An LDA decoder was trained using randomly sampled 2-s segments from blocks sharing the same goal pattern. Specifically, 15 segments were sampled from each of blocks 1 and 3 (goal pattern 1) and 30 segments from block 2 (goal pattern 2), thereby equalizing the total number of training samples across goal patterns. The trained decoder was then applied to the remaining 2-s segments from the entire recording session, including segments around goal transitions, to estimate the probability that each segment belonged to goal pattern 1 or goal pattern 2. This procedure was repeated 1000 times with independent random sampling, and decoding performance at each time segment was averaged across iterations to obtain a representative estimate for the session.

Chance-level performance was assessed using two complementary approaches. First, a shuffled-label control was implemented using the same decoding procedure, except that goal-pattern labels were randomly shuffled during decoder training. This procedure was repeated 1000 times to generate a distribution of chance-level decoding performance. The second control was to estimate whether these classifications were specific to goal changes. This was performed by artificially assigning three consecutive epochs within the same goal block to different classes (class 1 for the first and third segments, class 2 for the second segment) and applying the same decoding procedure to these data. This analysis was repeated for each of the three blocks, and decoding performance was averaged across blocks to estimate chance-level performance.

### Goal-pattern classification for pre-navigation spike sequences

To assess whether pre-navigation CA1 spike sequences occurring during immobility carried information about goal patterns, we applied a goal-pattern LDA decoder directly to the population spiking activity during individual spike-sequence events. While the goal-pattern decoder described in the preceding section used firing rates estimated by a Gaussian kernel with a bandwidth of 250 ms, the kernel bandwidth was reduced to 50 ms in the present analysis to capture population activity at the shorter timescale of spike-sequence events. For each sequence event, firing rates were averaged over its duration and fed into the LDA decoder to classify the underlying goal pattern.

### Statistical analysis

Statistical analyses were conducted using MATLAB (2024a) statistical toolbox (MathWorks). All statistical tests were two-sided and non-parametric unless stated otherwise. The plots and text display mean (line) ± s.e.m. (shaded). In box plots, the center line indicates the median, and the box limits indicate the 25th and 75th percentiles.

### Reporting summary

Further information on research design is available in the Nature Portfolio Reporting Summary linked to this article.

## Supplementary information


Supplementary Information
Peer Review file
Reporting Summary


## Source data


Source Data 1
Source Data 2


## Data Availability

Source data are provided with this paper. Additional raw data are available from the corresponding author upon request.

## Code availability

The analyses were performed using standard software packages described in the Methods. Custom code used for the analyses is available from the corresponding author upon request.
