# Supplementary material for: Prefrontal–thalamic goal states organize spatially aligned hippocampal maps
Source: Nat Commun. 2026 Aug 29;17:9233. doi: 10.1038/s41467-026-77240-6 (PMC13526029; doi:10.1038/s41467-026-77240-6)
Supplement: Supplementary file 1 [file 41467_2026_77240_MOESM1_ESM.pdf]

Extended Data Figures

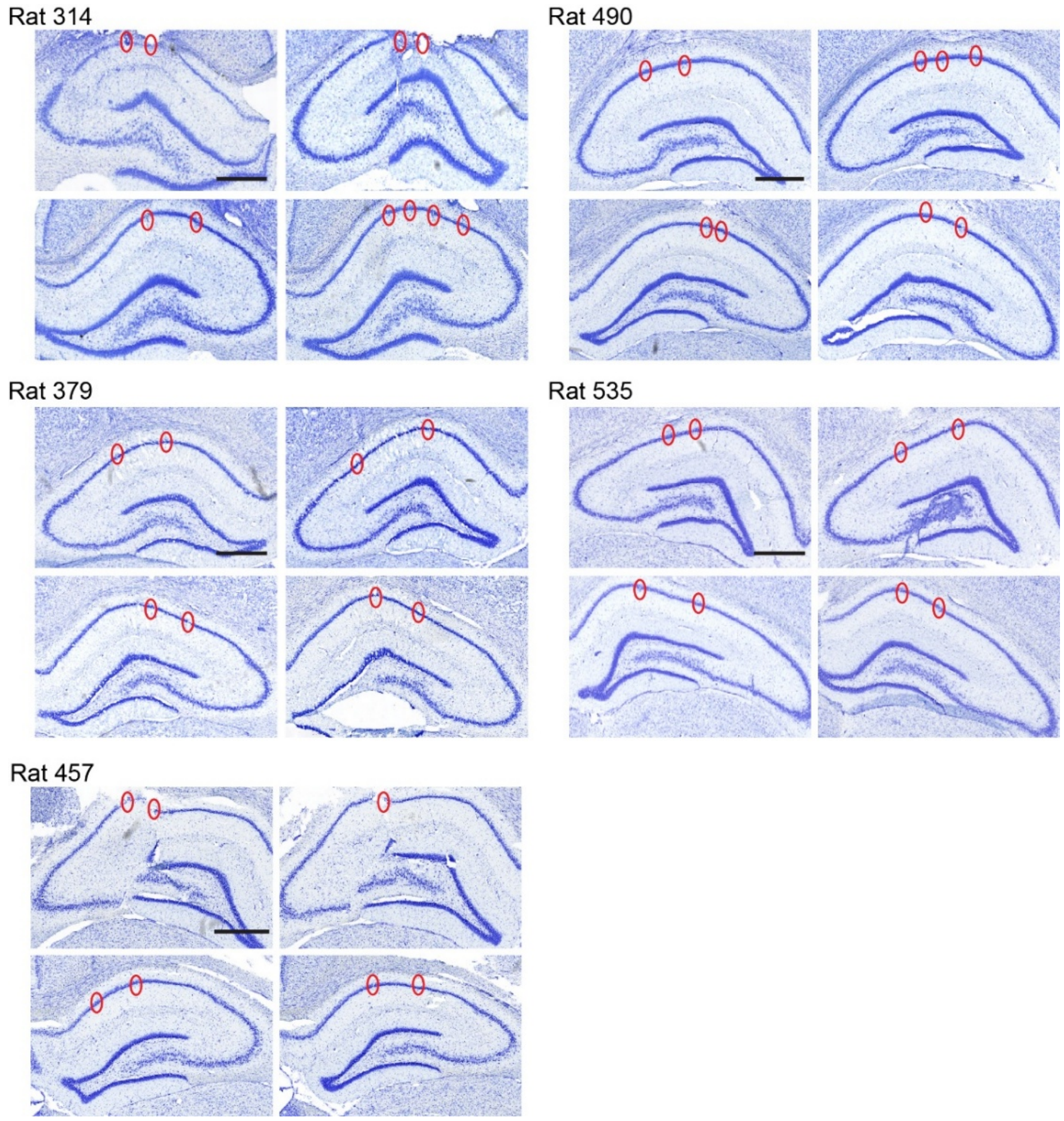

Extended Data Figure 1: Nissl-stained coronal sections showing electrode positions in hippocampal area CA1.

Tetrode tracks are marked by red circles. Tetrodes were implanted bilaterally in CA1. Scale bar = 1 mm.

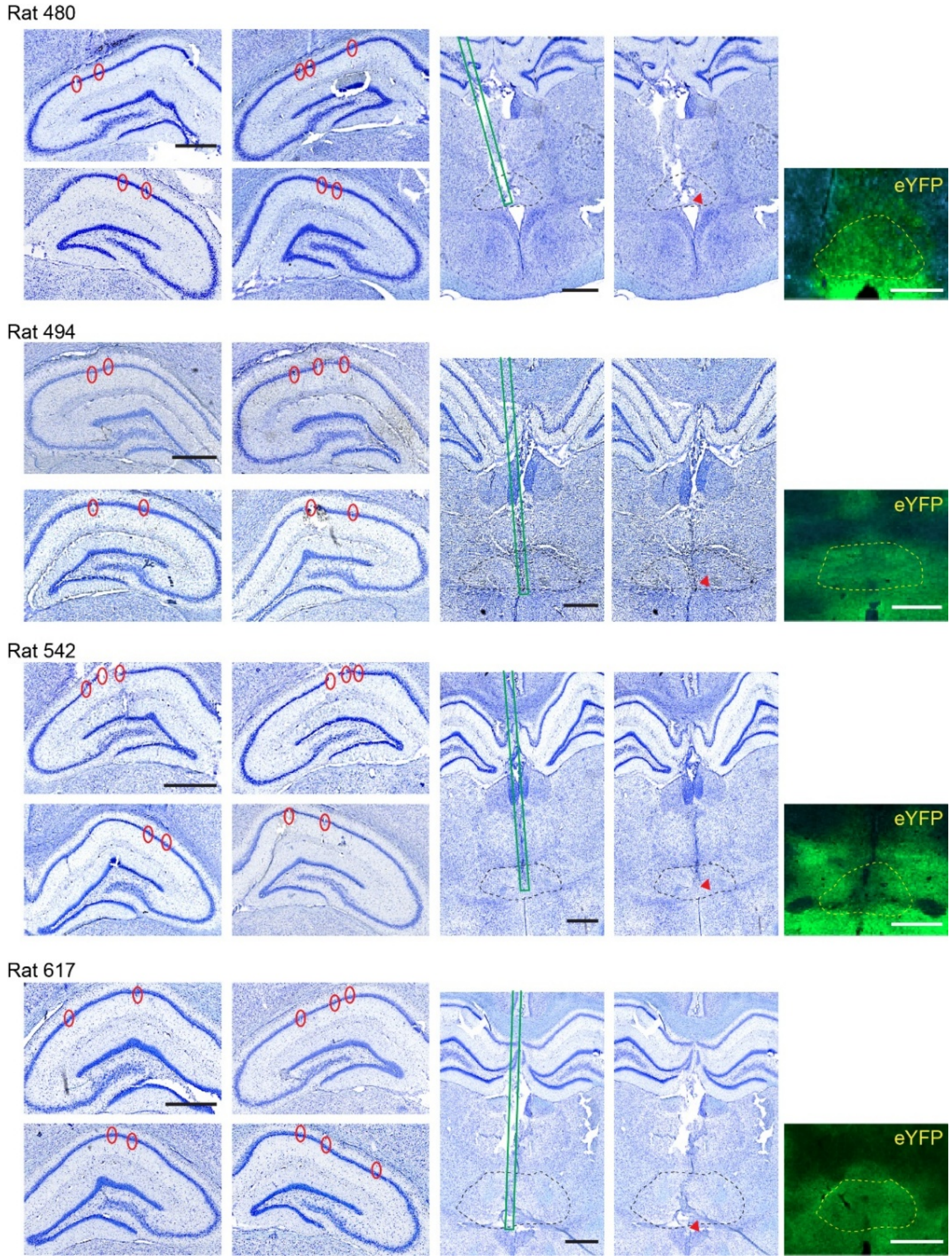

Extended Data Figure 2: Nissl-stained coronal sections showing electrode locations in hippocampal area CA1, together with optic fiber tracks and SwiChR++ expression in NR. Brain sections are from animals used for CA1 recordings during optogenetic silencing of NR. Tetrode tracks (red circles) confirm bilateral implantation in hippocampal area CA1. For each animal, the three rightmost panels show the optic fiber track (green line) targeting NR (black dashed outline), the same section with the fiber tip marked by a red triangle, and eYFP immunofluorescence (green) confirming SwiChR++ expression within NR (yellow dashed outline). Scale bar = 1 mm. Although viral expression extended beyond NR, we used a tapered Lambda fiber (400 µm diameter, 1 mm taper, 0.39 NA; OptogeniX) that directs illumination laterally along the taper rather than downward from the tip as in standard flat fibers, reducing the likelihood of affecting hypothalamic regions below the fiber. Nonspecific inactivation cannot be fully ruled out; however, these hypothalamic regions, specifically the dorsal and dorsomedial hypothalamic nuclei, send only sparse projections, if any, to the hippocampus.

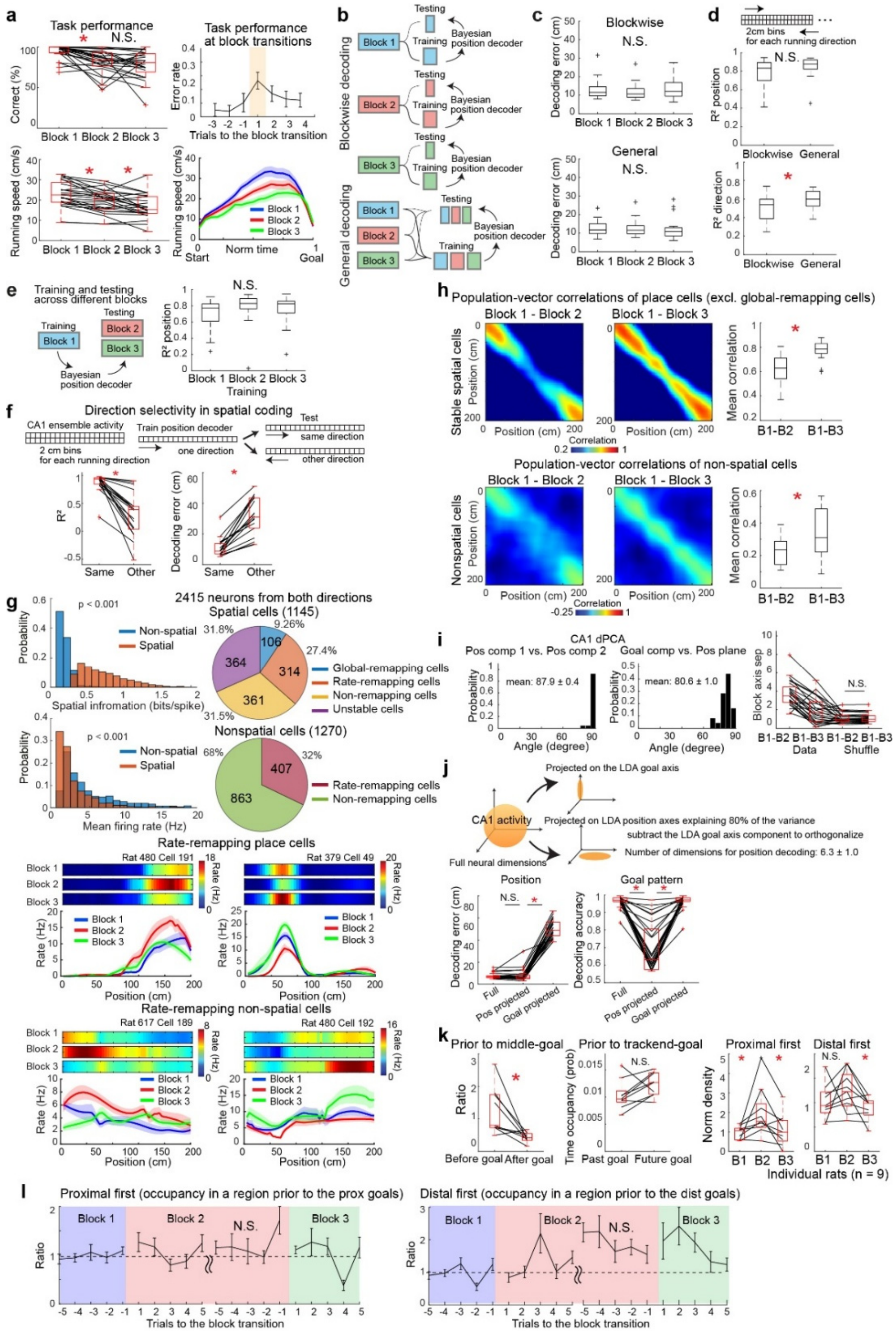

Extended Data Figure 3: Behavioral analyses, decoding performance, neuronal classification, and pre-navigation CA1 spike sequences.

a, Task performance. Top left, correct performance across blocks; top right, error rates at block transitions. Trial 1 denotes the initial journeys introducing a new goal, during which water was pre-delivered and LEDs beneath all wells were illuminated; animals rapidly adapted to goal changes. Bottom, running speed for individual journeys from the start to the goal wells, showing a gradual decrease over the session. $p < 0.05$, Wilcoxon signed-rank test. **b,** Schematic of the differences between the blockwise and general decoders. **c,** Left, position decoding error across blocks for the blockwise (top) and general (bottom) decoders. Kruskal–Wallis test. **d,** Coefficient of determination (R^2) for the two decoders, for position and direction estimation. The general decoder yielded significantly higher R^2 values overall. 25 sessions from 9 animals.

$p < 0.05$, Wilcoxon signed-rank test. **e,** Cross-block performance of the Bayesian position decoder. The decoder was trained on one block and applied to the other two. Performance was high (trained on block 1, $R^2 = 0.71 \pm 0.03$; block 2, 0.79 ± 0.04; block 3, 0.76 ± 0.03), with no significant difference by training block (Kruskal–Wallis, $p = 0.096$), further indicating no blockwise difference in decoding performance. **f,** Direction dependence of position decoding. The decoder was built from activity during one running direction and applied when the animal ran in the same or the opposite direction. Performance dropped markedly for the opposite direction, indicating direction-dependent spatial coding. $p < 0.001$, Wilcoxon signed-rank test. **g,** Left two panels, histograms of spatial information and mean firing rate for spatial and non-spatial CA1 neurons. $p < 0.001$, Kolmogorov–Smirnov test. Right, pie charts showing the proportions of CA1 cell categories, separately for spatial and non-spatial neurons. Bottom, representative spatial and non-spatial CA1 neurons showing rate remapping after goal changes. **h,** Population-vector (PV) correlations for place cells excluding global-remapping cells (top) and for non-spatial cells (bottom). Left, color-coded matrices of PV correlations at individual maze positions between blocks 1 and 2, and between blocks 1 and 3. Right, box plots of position-averaged PV correlations for each block pair. $p < 0.05$, Friedman test. **i,** Left, angles between the two position components and between the block component and the two position components extracted by dPCA, showing near-orthogonality of the axes. Right, axis separation for shuffled data. To generate the null, block labels were shuffled within matched position and running-direction bins; the original dPCA axes were retained and block-axis separation recomputed on the shuffled data. Under this null, block-axis separation was markedly reduced (shuffled = 1.12 ± 0.11; empirical: B1–B2 = 3.73 ± 0.29, B1–B3 = 1.91 ± 0.28), confirming that the original separation reflects genuine block-related structure. **j,** Orthogonal representations of position and goal in CA1 population activity. Activity was computed in 20 position bins for each of two running directions across three blocks. A goal classifier (LDA) discriminated the two goal patterns, yielding a single goal-coding axis. An LDA position decoder yielded 39 ($40 - 1$) axes, reduced to the components explaining 80% of the variance (6.3 ± 1.0 dimensions). From this position-coding subspace, the component projected onto the goal-coding axis was subtracted, giving a position subspace orthogonal to the goal-coding axis. Spatial and goal information were then compared using LDA decoding on the full neural space, the position subspace, or the goal-coding axis alone. Spatial information was largely preserved in the position subspace but not on the goal-coding axis, whereas goal information was confined to the goal-coding axis and largely absent from the position subspace. CA1 population activity thus orthogonalizes position and goal information within the neural manifold. **k,** Supporting analysis for Fig. 2e (left two) and Fig. 2g (right two), showing consistency across all nine animals. Box plots summarize 9 animals; some points are missing where no sequence events were detected in a given block. $p < 0.05$, Wilcoxon signed-rank test. **l,** Trial-wise density of pre-navigation sequences near the distal goal in proximal-first sessions (left) and near the proximal goal in distal-first sessions (right), as in Fig. 2g; regions of interest defined as in Fig. 2g. During block 2, sequence density at the block 1 goal was not reduced relative to its block 1 level, even as new goal-specific density emerged at the block 2 goal. Pre-navigation sequences thus appear to add a representation of the new goal rather than replace the previous one. During block 3, the density that had appeared at the block 2 goal was reduced, while the original block 1 representation remained intact. These results imply an asymmetry in goal updating: the first-block representation is maintained, whereas representations acquired in later blocks are more labile and can be attenuated when a further goal change is introduced.

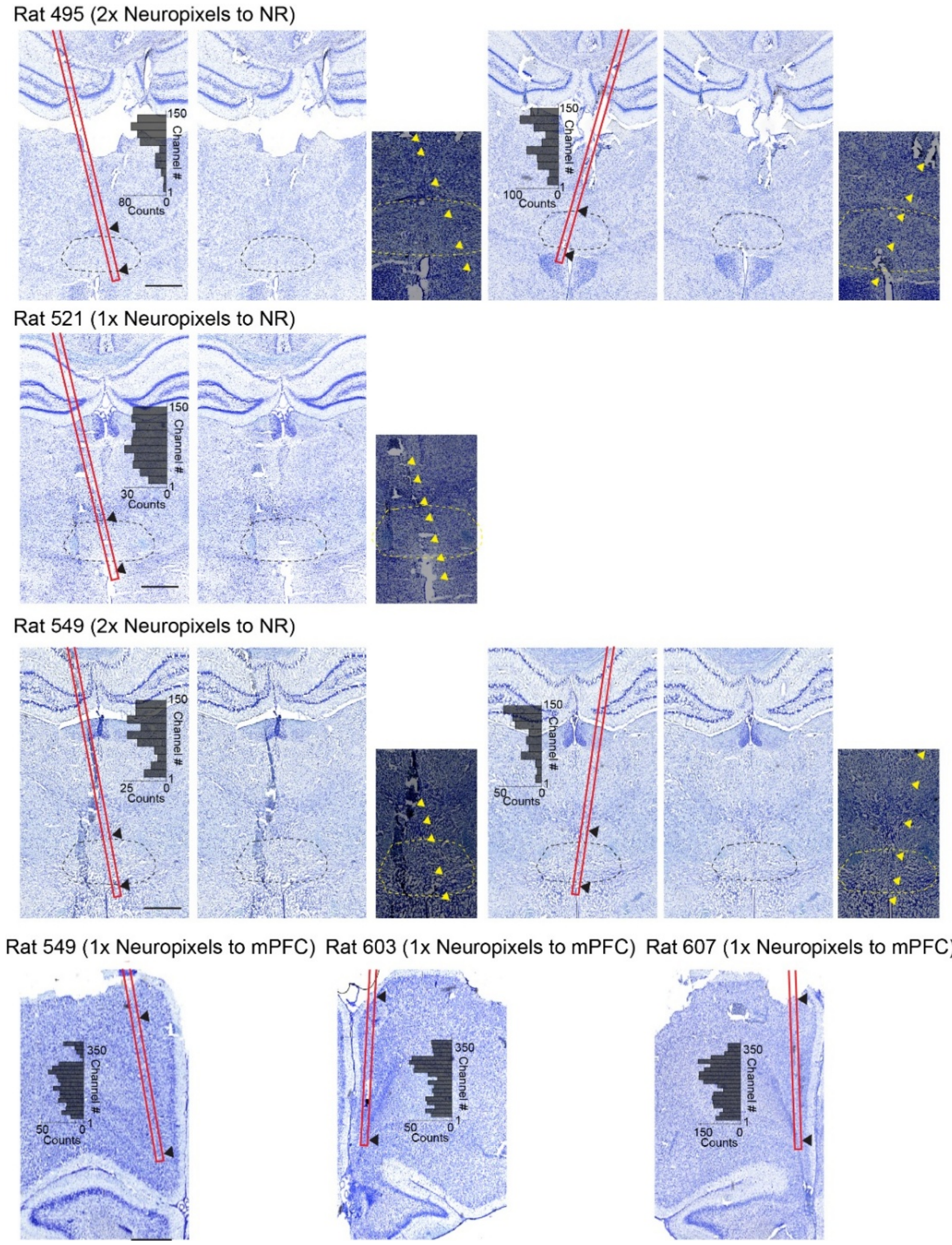

Extended Data Figure 4: Nissl-stained coronal sections showing the positions of Neuropixels probes in mPFC or NR.

Probe tracks are marked by red lines and NR is outlined by a black dashed line. Inset histograms show the distribution of channel positions for recorded cells within the probe region indicated by the two black arrows. For NR sections, the same image without the probe indicator (middle) and an enlarged image with adjusted color contrast (right) are also shown. Yellow arrowheads mark the estimated probe trajectory, inferred from local tissue separation, mechanical disruption, and cell displacement across adjacent sections.

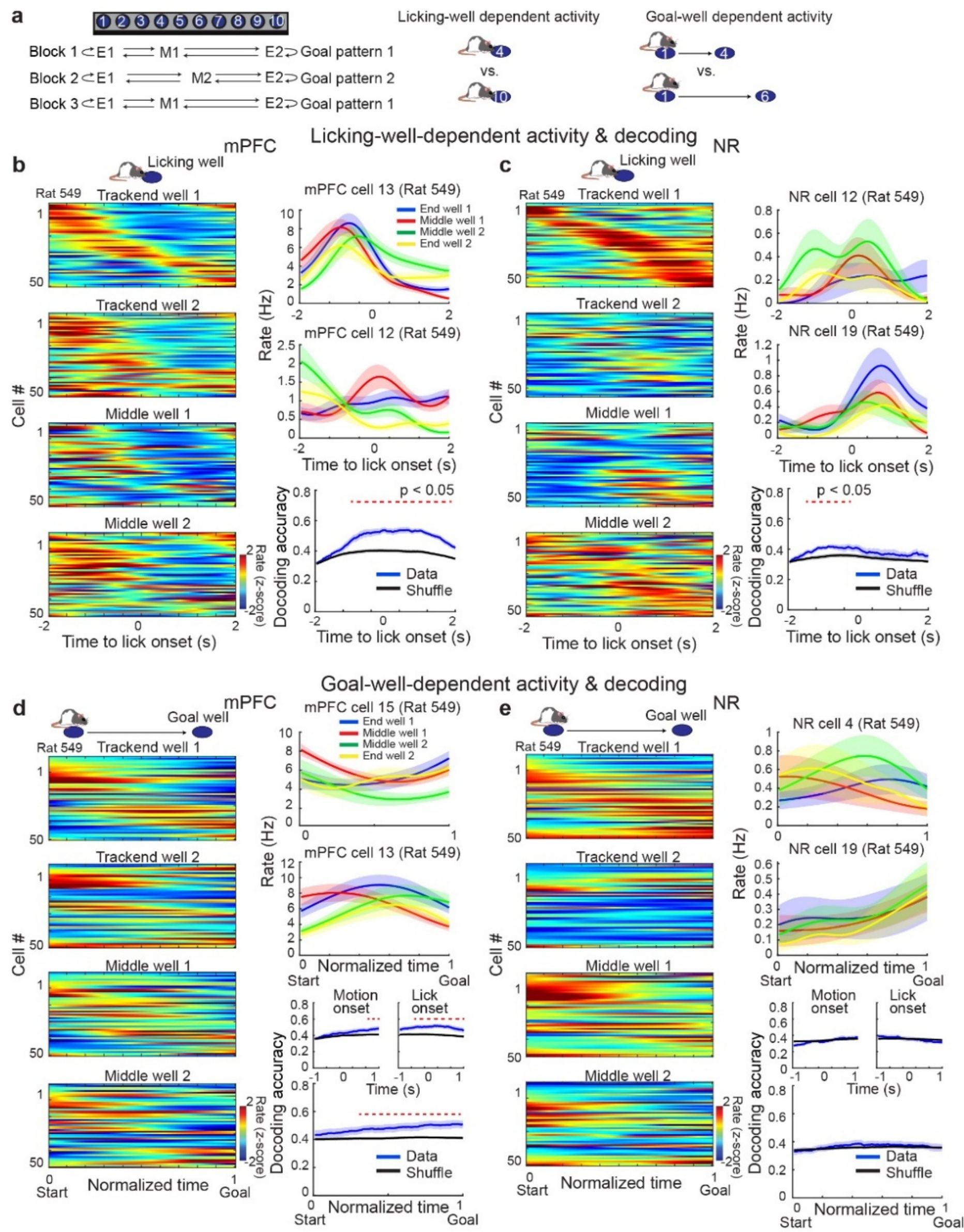

Extended Data Figure 5: Representations of licking wells and goal wells in mPFC and NR.

a, Schematic of the task structure, illustrating the distinction between licking-well-dependent and goal-well-dependent activity. **b,** Licking-well-dependent activity in representative mPFC neurons. Left, color-coded activity of 50 representative neurons aligned to lick onset. Right, mean firing rates of two example neurons and decoding performance for licking-well identity. Decoding used leave-one-out cross-validation (one trial for testing and the remaining trials for training). An LDA decoder was trained on z-scored firing-rate vectors in a −2 to 2 s window relative to lick onset, with trial numbers equalized across classes, and evaluated on the held-out trial; this was repeated for all trials. Chance was estimated two ways: (i) training on shuffled class labels, and (ii) controlling for running direction by splitting trials by direction and shuffling class labels within each direction group (an estimate of decoding based on directional differences alone). The larger of the two estimates was used as the chance level. Red dashed lines indicate $p < 0.05$, Wilcoxon rank-sum test. **c,** Same as **b**, for NR neurons. **d,** Goal-well-dependent activity in representative mPFC neurons. Left, color-coded activity of 50 representative neurons during navigation. Right, mean firing rates of two example neurons and

decoding performance for goal-well identity. An LDA decoder was trained on z-scored firing-rate vectors in windows from −1 s to navigation onset and from −1 s to goal-well licking onset. Chance estimation was as in **b**. Red dashed lines indicate $p < 0.05$, Wilcoxon rank-sum test. **e,** Same as **d**, for NR neurons. Together, these results indicate that, under these task conditions, neither mPFC nor NR showed robust prospective decoding of the upcoming goal-well location before navigation onset.

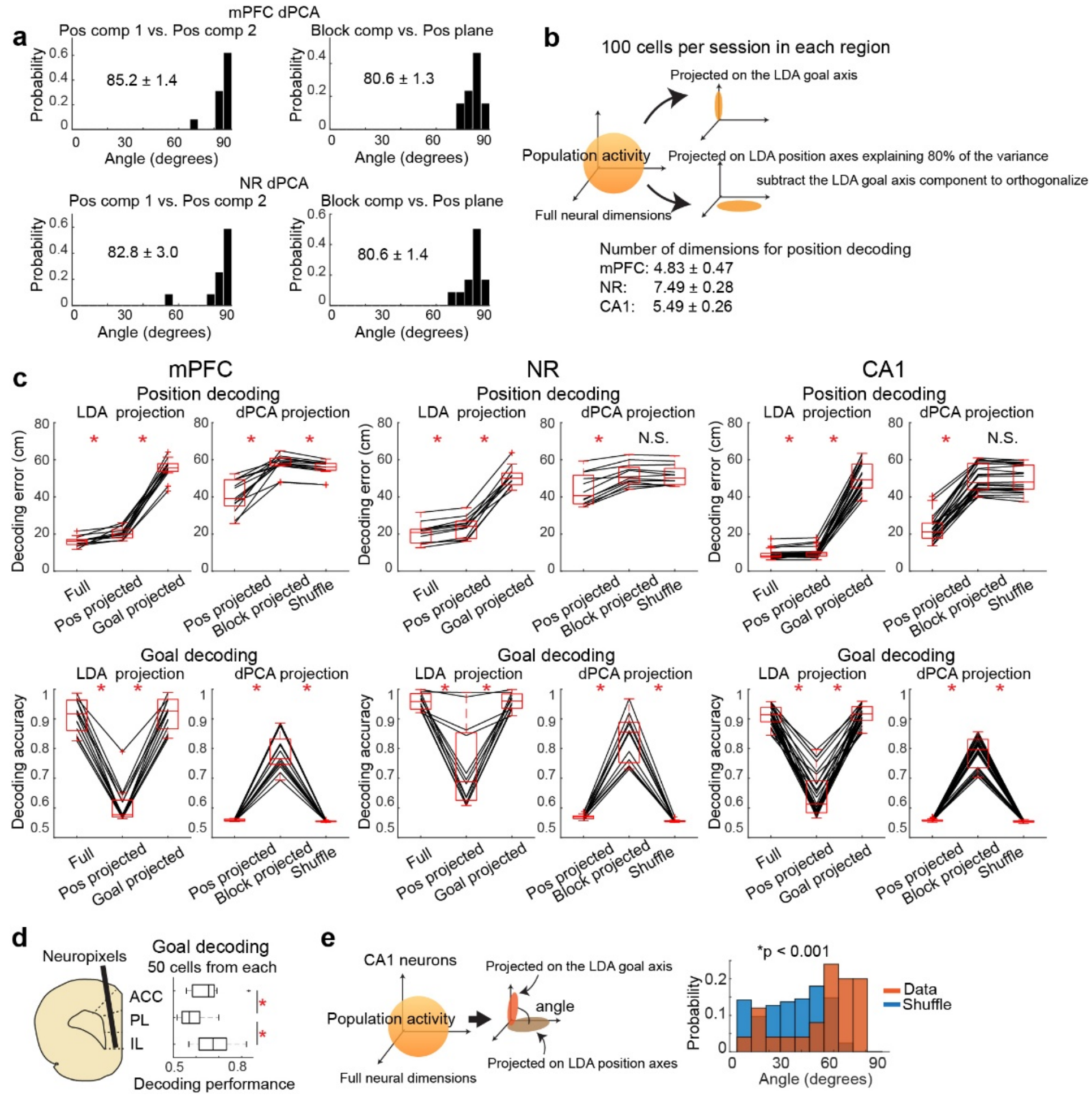

Extended Data Figure 6: Performance comparison of goal-state and position coding across mPFC, NR, and CA1.

a, Angles between the two position components and between the block component and the two position components extracted by dPCA, showing near-orthogonality of the axes. **b,** As in Extended Data Fig. 3j, an LDA goal classifier discriminated the two goal patterns, yielding a single goal-coding axis. An LDA position decoder yielded 39 (40 − 1) axes, reduced to the components explaining 80% of the variance. From this position-coding subspace, the component projected onto the goal-coding axis was subtracted, giving a position subspace orthogonal to the goal-coding axis. An equal number of neurons (100 cells) was used for each brain region. Spatial and goal information were then compared using LDA decoding on the full neural space, the orthogonalized position subspace, or the goal-coding axis alone. **c,** Summary of decoding performance across brain regions for the LDA-based projections defined in **b** and for dPCA-derived projections. **d,** Subregional differences in goal-state coding. Fifty cells were randomly sampled from each of three prefrontal subregions: infralimbic (IL), prelimbic (PL), and anterior cingulate cortex (ACC). Decoding performance differed significantly (*$p < 0.05$, Wilcoxon signed-rank test), indicating a regional bias in goal-state information. **e,** Orthogonal embedding of spatial and goal-state information in CA1. LDA decoders were constructed as in **b** but without the orthogonalization step, and the angle between the LDA projection spaces was computed. Values were compared with those from data shuffled across trial blocks and positions. The angle was significantly larger for the real data (*$p < 0.001$, Kolmogorov–Smirnov test), supporting near-orthogonal embedding of the two types of information.

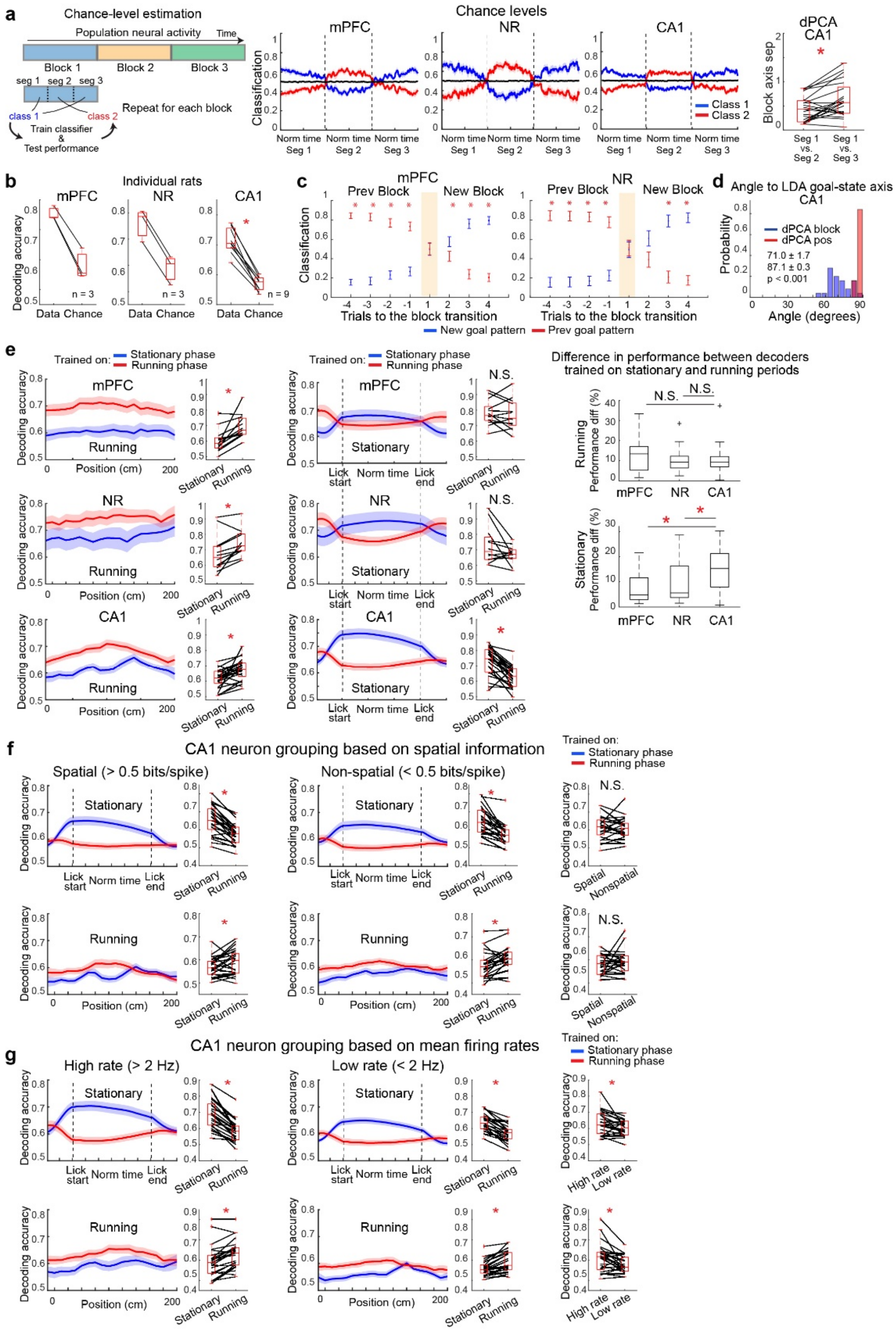

Extended Data Figure 7: Chance-level estimation and locomotor-state independence of goal-state coding in mPFC, NR, and CA1.

a, Estimation of chance-level goal-pattern decoding. To control for decoding unrelated to goal changes, three consecutive segments within the same goal block were artificially assigned to different classes (classes 1, 2, and 1 for the first, second, and third segments), and the same decoding procedure was applied. This was repeated for each of the three blocks and averaged to estimate chance-level performance. The three plots on the right show the resulting chance levels for each brain region. Far right, impact on dPCA separation along the block axis in CA1: dPCA was computed from the original data, but the arbitrarily time-segmented data within blocks were projected onto the dPCA axes. Axis separation was significantly larger between

segments 1 and 3 than between segments 1 and 2 (*p = 0.012, Wilcoxon signed-rank test) — i.e. separation grows for temporally more distant data, consistent with temporal drift of neural activity, a pattern opposite to that seen for true goal-state coding. **b,** Box plots of goal-pattern decoding performance for individual rats in each brain region, showing consistently higher performance than the chance levels estimated in **a**. **c,** Trial-by-trial goal-pattern decoding aligned to block transitions. Performance is significantly above chance ($p < 0.05$, Wilcoxon signed-rank test) and emerges rapidly after transitions. During the first cycle after each transition (shaded), water was pre-delivered at the goal wells and LEDs beneath the maze were illuminated to aid recognition of the new goal configuration. **d,** Angles between the goal-state classification axis (as in Fig. 4a) and the dPCA-derived axes in CA1. The goal-state axis is nearly orthogonal to the dPCA position components and more closely aligned with the dPCA block component. **e,** State dependence of goal-state representations. Goal-state classifiers were trained on neural activity from either stationary or running phases and applied to both states. Box plots summarize decoding performance. During running, classifiers trained on running-phase data outperformed those trained on stationary data. During stationary phases, classifiers trained on running-phase data performed comparably to those trained on stationary data in mPFC and NR, but not in CA1. This asymmetry suggests that goal-related representations generalize across locomotor states more strongly in mPFC and NR than in CA1. $p < 0.05$, Wilcoxon signed rank test for the within-region comparisons and Wilcoxon rank-sum test for the between-region comparisons. **f,** Contribution of CA1 cell types to goal-state decoding. CA1 neurons were split by spatial information content (high or low relative to 0.5 bits/spike), with cell numbers matched between groups within each session before decoding. No significant difference in decoding performance was observed, indicating that goal-state coding is not preferentially driven by either spatially tuned or weakly spatial neurons. **g,** Contribution of CA1 neurons categorized by mean firing rate. CA1 neurons were split into high- and low-firing-rate groups (threshold, 2 Hz), with cell numbers matched. Goal-pattern decoding was significantly higher for high-firing-rate neurons, suggesting they contribute disproportionately to goal-state coding.

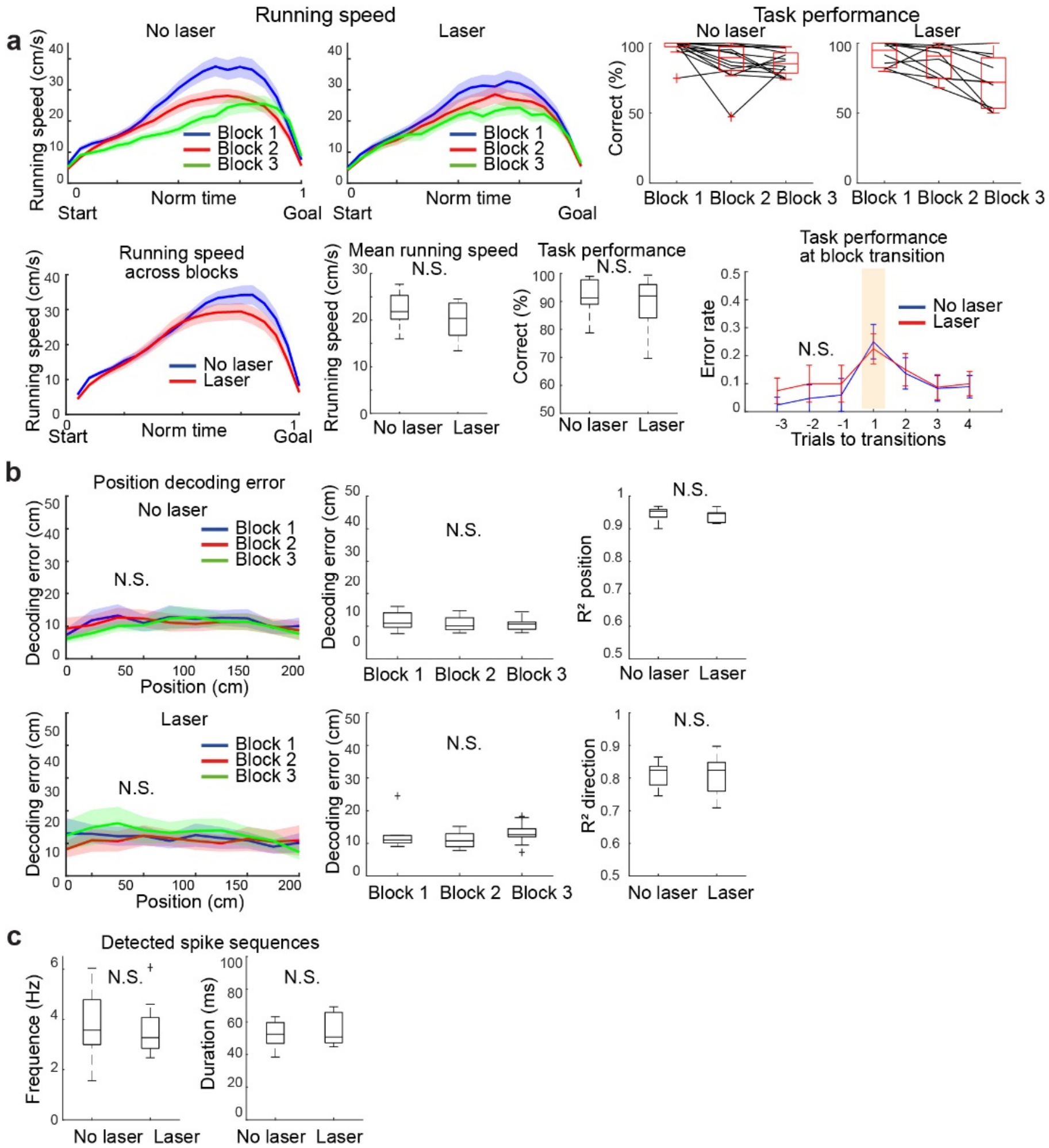

Extended Data Figure 8: NR silencing did not significantly affect task performance, position decoding, or CA1 spike sequence frequency or duration.

a, Top left, running speed during goal-directed journeys across blocks, with and without laser. Top right, box plots of task performance across blocks, with and without laser. Bottom left, average running speed for laser and no-laser sessions. Bottom middle, box plots comparing running speed and task performance. Bottom right, error rates at block transitions. No significant differences were observed between conditions. $p > 0.05$, Wilcoxon rank-sum test. **b,** Left, position-decoding errors from CA1 population activity across maze positions, with and without laser. Middle, position-decoding errors across blocks ($p > 0.05$, Kruskal–Wallis test). Right, box plots comparing the coefficient of determination (R^2) for decoded position and running direction between laser and no-laser sessions ($p > 0.05$, Wilcoxon rank-sum test). No significant differences were observed. **c,** Detected spike-sequence events. Neither the frequency nor the duration differed significantly between laser and no-laser sessions. $p > 0.05$, Wilcoxon rank-sum test.

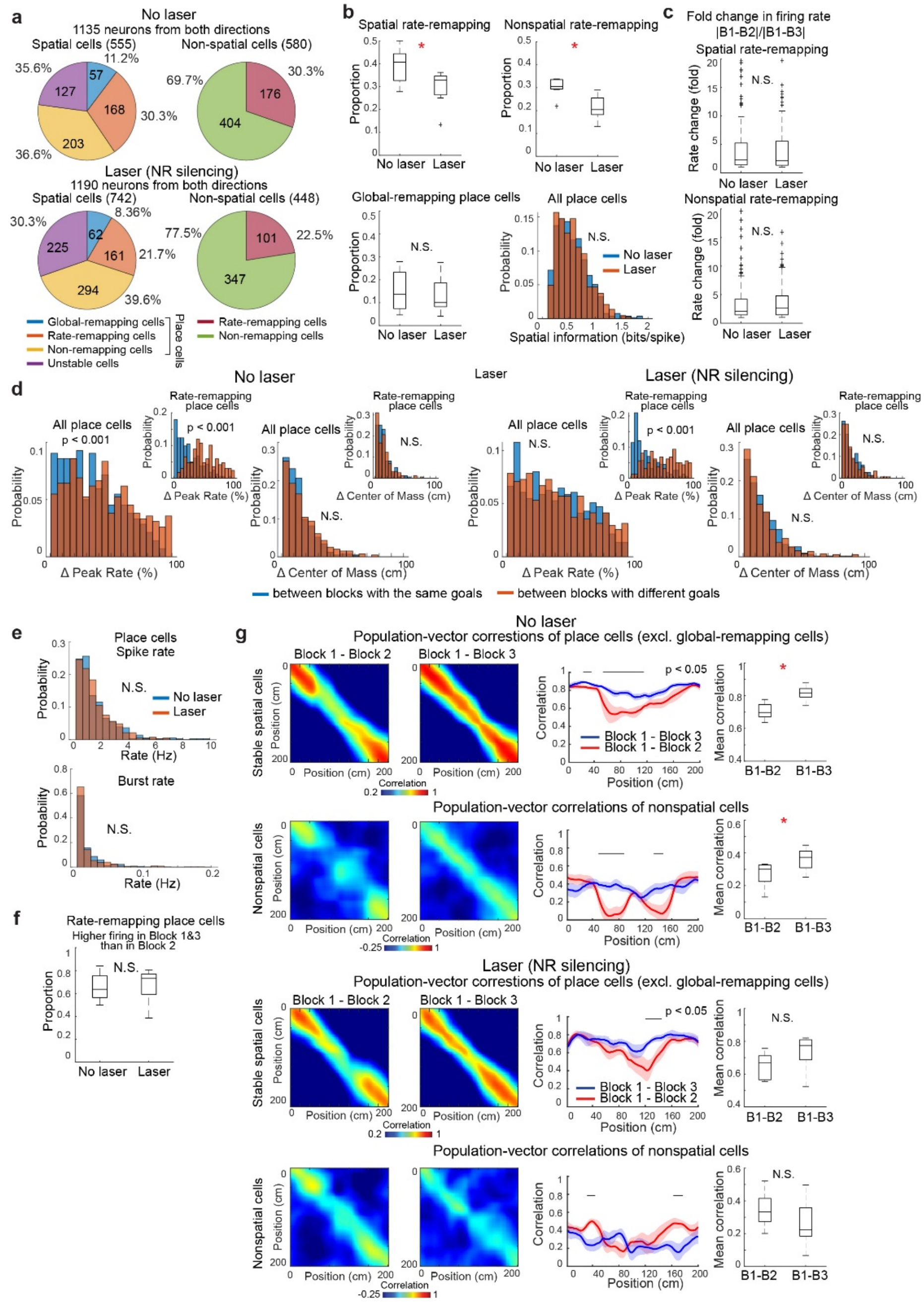

Extended Data Figure 9: Reduction of CA1 rate remapping by NR silencing.

a, Pie charts of the proportions of CA1 cell categories, plotted separately for spatial and non-spatial neurons. Data are from 4 animals, with sessions performed with and without laser. **b,** Proportions of rate-remapping place cells (top left), non-spatial rate-remapping cells (top right), and global-remapping place cells (bottom left). The proportion of rate-remapping cells differed significantly between sessions with and without laser (rate-remapping place cells: Friedman test, $\chi^2(1) = 5.40$, $p = 0.020$; non-spatial rate-remapping cells: $\chi^2(1) = 5.40$, $p = 0.020$), but global-remapping cells did not ($\chi^2(1) = 0.60$, $p = 0.439$). Bottom right, distribution of spatial information for all place cells, showing no significant difference between conditions (Kolmogorov–Smirnov test, $p = 0.934$). **c,** Fold changes in firing-rate modulation comparing blocks 1–2 (different goal patterns) with blocks 1–3 (same goal pattern), for rate-remapping place cells (top) and non-spatial rate-remapping cells (bottom). No significant differences

between laser and no-laser sessions. $p > 0.05$, Wilcoxon rank-sum test. **d,** Histograms of peak-firing-rate differences between same-goal blocks (blue) and different-goal blocks (red), for sessions with and without laser. In control sessions, place cells showed a significant difference in rate-change distributions between blocks 1–2 and 1–3 (*$p < 0.001$, Kolmogorov–Smirnov test), whereas this block-pair difference was not detectable during NR silencing ($p = 0.729$). Center-of-mass shifts of place fields remained stable across blocks with different goals and were unaffected by NR silencing. **e,** Spike rate (top) and burst rate (bottom) for place cells with and without laser. A burst was defined as ≥3 spikes with all consecutive inter-spike intervals ≤6 ms. No significant differences ($p > 0.05$, Kolmogorov–Smirnov test). **f,** Proportion of rate-remapping place cells with higher firing rates in blocks 1 and 3 than in block 2. For each rate-remapping neuron, we asked whether its firing rate was higher in blocks 1 and 3 or in block 2. About 60% of rate-remapping place cells showed higher rates in blocks 1 and 3, indicating a modest baseline asymmetry in the population. This proportion was not significantly altered by laser ($p = 0.439$, Friedman test), suggesting that NR silencing did not bias the relative prevalence of neurons preferring either goal configuration. **g,** Same analysis as in Extended Data Fig. 3h, comparing sessions with and without laser. Black lines in the middle panels indicate significant effects ($p < 0.05$, Friedman test). Right, comparison between blocks 1–2 and 1–3. Mean population-vector (PV) correlations for place cells excluding global-remapping cells differed significantly in control sessions ($\chi^2(1) = 7.35$, $p = 0.007$) but not during NR silencing ($\chi^2(1) = 3.75$, $p = 0.053$). For non-spatial rate-remapping cells, differences were significant without laser ($\chi^2(1) = 9.60$, $p = 0.002$) but not with laser ($\chi^2(1) = 0.00$, $p = 1.000$). Data are from 8 sessions across 4 animals.

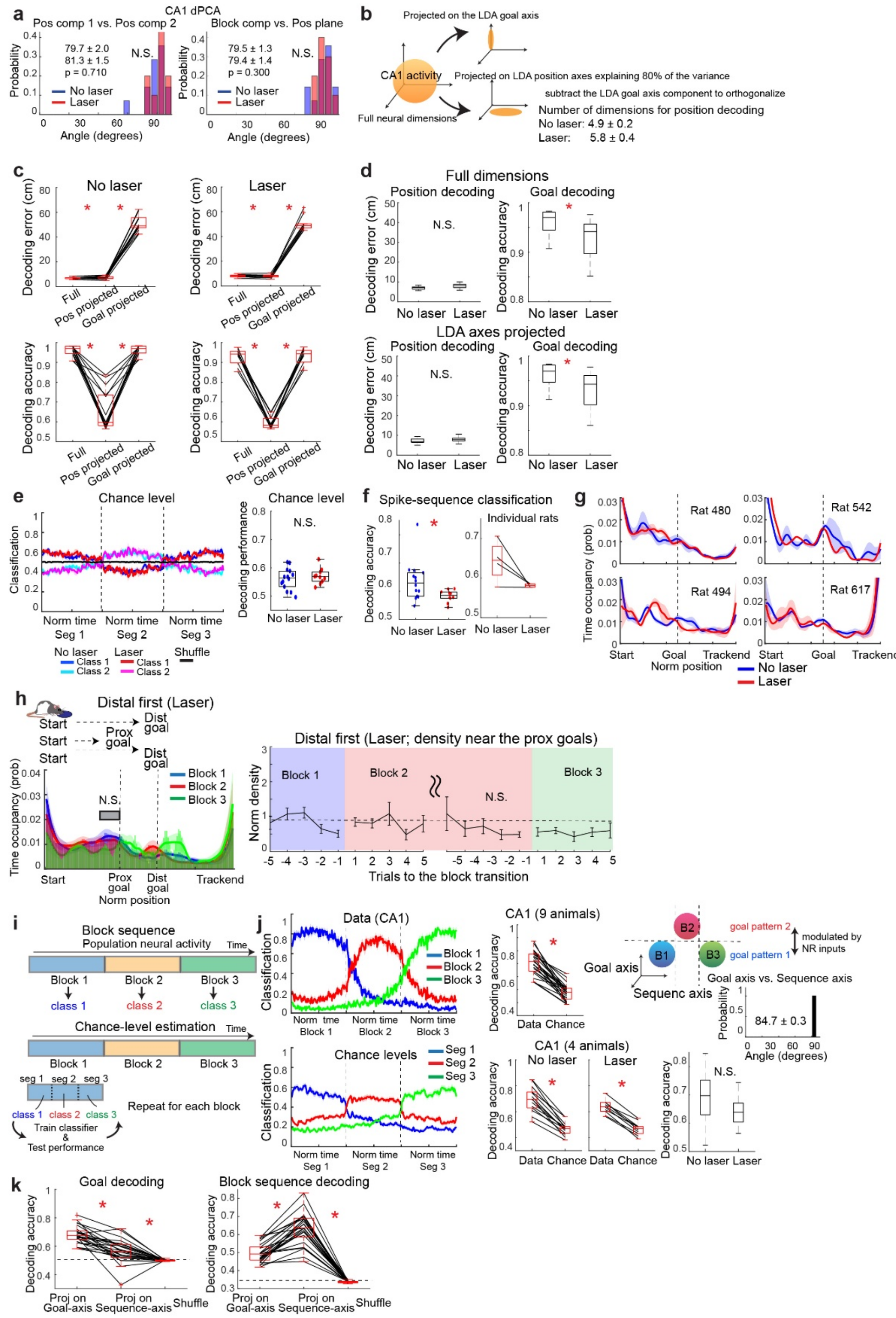

Extended Data Figure 10: Selective effects of NR silencing on CA1 goal-state coding and pre-navigation spike sequences.

a, Angles between dPCA-derived components in sessions with and without laser. No significant difference. $p > 0.05$, Kolmogorov–Smirnov test. **b,** As in Extended Data Fig. 3j, a goal-pattern classifier (LDA) discriminated the two goal patterns, yielding a single goal-coding axis. An LDA position decoder yielded 39 (40 − 1) axes, reduced to the components explaining 80% of the variance. The projection onto the goal-coding axis was subtracted from the position-coding subspace, giving a position subspace orthogonal to the goal-coding axis. **c,** Summary of decoding performance for sessions with and without laser using the LDA-based projections

defined in **b**: position decoding error (top) and goal-pattern classification accuracy (bottom). *$p < 0.05$, Wilcoxon signed-rank test. **d,** Decoding performance using full-dimensional LDA classifiers (top) and the LDA-based orthogonal projections defined in **b** (bottom). $p < 0.05$, Wilcoxon rank-sum test. **e,** Estimation of chance-level goal-pattern decoding, as in Extended Data Fig. 7a. Chance-level performance did not differ between sessions with and without laser. **f,** Classification performance of pre-navigation spike sequences in sessions with and without laser, for all sessions (left) and individual animals (right). $p < 0.05$, Wilcoxon rank-sum test. **g,** Time occupancies of pre-navigation CA1 sequences with and without laser for individual rats; averages across animals are shown in Fig. 5i. **h,** Same analysis as in Fig. 2f, applied to sessions with NR silencing. Left, time-occupancy probabilities of pre-navigation spike sequences for trials targeting a distal goal in blocks 1 and 3 and a proximal goal in block 2. Right, trial-wise densities normalized to block 1. No significant differences across blocks. $p > 0.05$, Wilcoxon signed-rank test. **i,** Classification performance of block-order progression from randomly sampled 2-s neural-activity segments, irrespective of locomotor state (top), and the corresponding chance-level estimation (bottom). **j,** Left, classification performance for temporal sequences of blocks (top) and corresponding chance levels (bottom). Middle and right, box plots of decoding performance across all sessions from nine animals (top) and comparisons of the same four animals with and without laser (bottom). $p < 0.05$, Wilcoxon signed-rank test. The schematic (top right) illustrates orthogonal neural coding of goal-state and temporal-sequence information, supported by the near-orthogonal distribution of angles between these axes. **k,** Separability between goal and sequence information. Orthogonal LDA decoders for goal and block sequence were constructed as in **b**. Goal decoding was significantly better for activity projected onto the goal-coding axis than onto the sequence axis, whereas block-sequence decoding was significantly better for activity projected onto the sequence axis, supporting separate embedding of the two information types.
