# Supplementary material for: Prefrontal–thalamic goal states organize spatially aligned hippocampal maps
Source: Nat Commun. 2026 Aug 29;17:9233. doi: 10.1038/s41467-026-77240-6 (PMC13526029; doi:10.1038/s41467-026-77240-6)
Supplement: Supplementary file 2 [file 41467_2026_77240_MOESM2_ESM.pdf]

nature portfolio

Peer Review File

Prefrontal–thalamic goal states organize spatially aligned hippocampal maps

Corresponding Author: Professor Hiroshi Ito

This file contains all reviewer reports in order by version, followed by all author rebuttals in order by version.

Version 0:

Reviewer comments:

Reviewer #1

(Remarks to the Author)
Summary
The authors explore how the hippocampus represents contextual information, where context is modulated by goal location along a linear track. Using electrophysiological recordings in rats they show activity in hippocampus (CA1) is segregated by context along a population dimension that is near orthogonal to spatial coding. This contextual code can also be observed in medial prefrontal cortex (mPFC) and nucleus reunions (NR). While silencing the NR does not affect task performance, it does significantly affect the population vector in CA1, which no longer shows significant difference between blocks with different vs identical goal configurations, while sparing spatial coding.

Overview: A very comprehensive manuscript with detailed analysis. Claims are largely supported by the data. A few suggestions below.

Major
1. Is the use of the term ‘episode-specific’ appropriate given this task? Surely ‘context’ would be more appropriate? Using the term episode-specific suggests that the task captures inherent features of episodic memory. Yet this task does not capture the rich what/where/when features of an episodic memory. Instead the mice are exposed to a given context for several minutes (up to 15 consecutive trials). It seems misleading to therefore use the term ‘episode-specific’, both in the title and throughout the Discussion.
2. Are the hippocampal cells that discriminate between blocks 1/3 vs 2 akin to a splitter cell? If so, how are the reported findings novel? If not, what is different about this neural code and a splitter cell code?
3. Can the authors make clearer how this paper builds on previous studies that have applied optogenetic manipulations to NR to examine the effect of NR on hippocampus? Previous work (e.g. https://pubmed.ncbi.nlm.nih.gov/29940254/, https://pubmed.ncbi.nlm.nih.gov/30282726/) would suggest that the NR hippocampal projection indeed provides goal relevant and contextual information. Can the authors articulate more clearly what is new here?
4. Figure 3e shows that CA1 better encodes space (which we would expect), but all recorded brain regions (mPFC, CA1 and NR) encode goal location. How specific is the goal location encoding to this network of regions, or is this a brain wide code? Is there a control brain region where goal encoding is not observed?
5. Ln 178: which principle components explain the block and position components? If not the first 3 components then how were they selected? Seems like the block component is only explained by very small % variance?
6. Ln 377: NR inactivation does not impair task performance. This implies that NR input to hippocampus is not necessary for the task, suggesting that task-relevant neural codes are represented in other brain regions?
7. Figure 5i: looks like there is no effect of NR laser in two out of four rats? Why is this? Is there an effect overall across rats? What proportion of points in the upper plot come from the two rats showing an effect, vs the two rats not showing an effect?
8. Fig 5e suggests that when NR is silenced there isn’t a deficit in distinguishing contexts but in recognising that two contexts are the same. Data in 5f also suggests this may be the case. This seems somewhat different to the narrative presented throughout the manuscript.
9. Fig5j: how are these windows for comparison chosen without introducing a multiple comparisons problem?

Minor

1. In 310-311 may want to edit this statement to say ‘in this task, population activity in mPFC and NR primarily functions…’ as there are of course other primary functions of these brain regions which cannot be revealed by this task.
2. Ln 334: not clear what is being trained and tested? What is meant by goal patterns being reliably decoded? Do they train on goal vs not goal location and then test on held out data samples? How are these not simply spatial representations?
3. Figure 2h is not clear: what is being shown and which statistical test do the red stars refer to?
4. Figure 4c and 4g: while the results look compelling, would it not be more robust to compare the group mean of the data against a null distribution generated using permutation tests, with >1k shuffles?
5. In Figure 5f, left hand panels, is there a significant interaction between on and off laser? An interaction is shown in right hand panel but with a different y axis label.
6. Do the authors distinguish between the pyramidal neurons and interneurons in their analysis? Why are putative interneurons included, for example in Fig1c, Rat617, Cell146 looks like an interneuron.

Reviewer #2

(Remarks to the Author)
This is a thoughtful and technically sophisticated manuscript that addresses how hippocampal population activity can preserve spatial information while also distinguishing different goal-defined navigation episodes. Using a well-controlled A/B/A goal-switching design, large-scale recordings from CA1, mPFC, and nucleus reuniens, population-level decoding/dPCA analyses, and optogenetic NR perturbation, the authors present evidence that CA1 encodes goal or task-context information in a dimension that is largely separable from position coding. The study has several notable strengths: the behavioral design allows the same spatial environment to be examined under different goal configurations; the analyses attempt to dissociate spatial coding, goal-state coding, and pre-navigation sequence content; and the NR silencing experiment provides a potentially important causal link between thalamic activity and CA1 goal-context representations. The combination of hippocampal population geometry, pre-run sequence analysis, and circuit perturbation makes the work potentially significant. However, several aspects of the analysis and interpretation require clarification or additional controls before the strongest claims are fully supported. In particular, the manuscript would benefit from more rigorous anatomical validation of recording/manipulation sites, clearer definitions of the spatial binning and normalization used in sequence-occupancy analyses, stronger null models for the orthogonality and cross-block separation claims, and more conservative language regarding mPFC–NR–CA1 pathway causality.

1. The manuscript shows Nissl-stained sections with tetrode and optic fiber placements, and Fig. 3a appears to show representative Neuropixels tracks in mPFC and NR. However, the manuscript does not clearly provide systematic reconstructions of Neuropixels recording sites across animals, including the estimated locations of recorded units along the probe trajectory. This is particularly important for NR recordings, given the small sample sizes used here and the proximity of neighboring thalamic and hypothalamic nuclei. Authors should provide atlas-registered probe tracks and estimated recording-site locations for each Neuropixels animal/session, together with criteria for assigning units to mPFC or NR and excluding off-target recordings.
2. Similarly, the Nissl-stained sections shown in Extended Data Figure 2 are intended to show optical fiber tracks. However, despite the visual annotation, the fiber tracks themselves are not visually discernible. This is particularly important in this study, because the fluorescent histological sections accompanying these show strong expression in the hypothalamus, in some cases stronger than in NR. If the histological images themselves, without the estimated fiber locations overlaid, cannot confirm fiber placement, then a separate experiment, in which fibers placement is confirmed using dye-coated fibers or thermolytic lesioning, is in order.
3. Figure 1g shows similar decoding accuracy with blockwise vs general decoding, but this may not be the most sensitive way of detecting spatial coding differences across blocks. This result is used to substantiate the claim that location coding is unchanged across blocks or goal states. A more direct test would be to see decoders trained on each block and tested on each other block. The higher population vector correlation between blocks 1 and 3 than between 1 and 2 (EDF 3g) makes this especially important to show.
4. Extended Data Figure 3h presents a finding of ‘nearly-orthogonal’ spatial and goal components – but orthogonal relative to what null distribution? This is a concern in high-dimensional state space, and derivations of such a space, which tends to drive vector angles toward orthogonality.
5. Figure 1j is presented in arbitrary units. To make this result interpretable, a null comparator should be presented using a position- and direction- matched shuffle across blocks. If enough trials worth of data are available, it would also be good to see a block-to-self (e.g. B1-B1, B2-B2, and B3-B3) comparison, to determine the ceiling for this axis separation effect. Figure 1k contains a Shuffled condition, but it is not clear how this shuffle was performed, i.e. whether position and direction were held constant across blocks.
6. The cross-block claims would be bolstered by some additional controls for temporal drift. CA1 population activity drifts over time, and an A/B/A design helps but does not completely eliminate temporal confounds. A useful temporal-drift control would be to assign pseudo-blocks of matched duration by circularly shifting block boundaries or placing pseudo-boundaries within blocks, then recomputing, e.g., dPCA separation. The observed goal-block separation should exceed this pseudo-block null.
7. The effect shown in 2e is subtle, and I am therefore concerned with how the spatial bins are grouped for analysis. The authors describe a process of some sort of spatial normalization (Line 759, “The time occupancies of sequence representations were estimated at every 2.5 cm bin for each recording session and normalized for the distances of the middle or trackend goal locations”), but it is not clear what is meant by this. The regions delineated for comparison in Figure 2e are described as ‘quadrants’ – how were these regions defined, precisely? The regions annotated in the trackend comparison (2e right) look smaller than the ‘quadrants’ in 2e left – are they? Why did the authors switch from quadrants to ‘regions of interest’ for the pre-running sequence analysis (2f), and how were these location sizes determined? Then once again they switch back to another definition for spatial location groupings for Figure 5 (“…ratio of occupancy near the

upcoming goal…”). It would be helpful to show that these effects are robust to changes in the spatial groupings.
8. I had trouble interpreting Extended Data Figure 3k. What does it mean to have a probability >1 for ‘slope to goal’? if the y-axis is mean to somehow denote the slope of the sequence occupancy trace before and after goal approaches, it is still not clear why slope and not magnitude is the relevant metric here, or over what set of spatial bins this slope was computed.
9. 2g only shows occupancy for distal goal in proximal-first sessions, and for the proximal-first goal in distal-first sessions. it would be helpful to see occupancy decline in the regions associated with block 1, as occupancy shifts toward the block 2 goal during block 2.
10. In Extended Data Fig. 4, the authors should explain why chance decoding is above 0.5 for all three regions – is this a result of trial number imbalance across conditions?
11. In several places, the manuscript uses language that appears stronger than what is directly supported by the presented data. For example, line 310-311 states: “population activity in mPFC and NR primarily functions to segregate navigation episodes associated with different goal patterns”. This claim overstates what can is demonstrated here, as these results do not establish what the “primary functions” of mPFC and/or NR are during this task. What is supported by Figure 3 is that population activity in both structures is associated with goal states more reliably than with location.
12. Another overstated claim, which is repeated throughout, is that the orthogonal goal-state representation observed in HPC is transmitted from mPFC to NR to HPC. For example, the abstract says: ”This goal-state signal is supplied by a prefrontal–thalamic pathway, in which population activity in medial prefrontal cortex and nucleus reuniens forms persistent representations”. Similarly, in the intro (line 77): “…we examine how goal-related information is causally transmitted to the hippocampus”. And in line 441: “In this study, we show that prefrontal top-down input routed through the mPFC–NR–CA1 pathway enables the hippocampus to distinguish distinct navigation episodes…” What the authors have demonstrated instead is a correlation between the observed orthogonal goal-state signal in HPC and mPFC and NR activity, combined with causal evidence that NR activity is critical for the HPC representation. This does not rule out the possibility that the goal state signal is transmitted via a broader circuit.
13. The authors should report first-order effects of NR silencing on CA1 activity, including mean/median firing rates, firing-rate distributions across neurons, fraction of active cells, and burst statistics if available. This is important for distinguishing a selective disruption of goal-state coding from a more general change in CA1 excitability, gain, or network state. The rate-remapping analyses appear to quantify unsigned changes in firing rate. It is therefore unclear whether goal-related rate remapping reflects a balanced redistribution of activity across CA1 cells, a global increase/decrease in CA1 firing in block 2, or selective modulation of positively versus negatively tuned neurons. The authors should report signed firing-rate changes across blocks, including the fractions of cells increasing versus decreasing from block 1 to block 2, and test whether NR silencing preferentially affects either sign of modulation.
14. The NR inhibition effects on goal occupancy in the pre-running sequences are presented as a probability ratio (Figure 5j). The interpretation would be strengthened by presenting these ratios alongside raw occupancy curves with confidence intervals, rather than the ratios alone. It would also be helpful to see animal-level statistics, a mixed-effects model with session nested within animal, and permutation/bootstrap controls.

Minor issues:
1. The manuscript relies heavily on findings presented in Extended Data figures, but references to these figures leave out the figure panel, citing only the figure number. This makes it difficult to discern what evidence is being used to support what claim. References to extended data figures should cite which panel is being used to substantiate each specific claim.
2. Typos: Extended data figure 3g: “Population-vector correstions”
3. Some of the figure panels are out of order – e.g. 5e and 5f

Version 1:

Reviewer comments:

Reviewer #1

(Remarks to the Author)
While the authors have made a number of changes to the manuscript, three of the major points I raised in round 1 have not been addressed. I therefore have outstanding comments which I list below.

1. Regarding my first point in round 1: “Is the term “episode-specific” appropriate given the task? Etc…”
The authors have not addressed my concern that the term “episode-specific” in the title is misleading (point 1 of my previous review). So called “episodes” are actually multiple trials in a particular context, while use the term “episode-specific” implies that the task and analyses make claims about episodic memory, which is not the case.

2. Regarding my second point in round 1: “Are the hippocampal cells that discriminate between blocks 1/3 vs 2 akin to a splitter cell? If so, how are the reported findings novel? If not, what is different about this neural code and a splitter cell code?”
The authors additional reference to splitter cells in the revised Discussion section doesn’t account for theories that would encompass the data presented in the paper. In particular, splitter cells may reflect the underlying latent state or temporal context (https://elifesciences.org/articles/82357). While the authors suggest their data are novel because the rate remapping is not driven by externally perceived sensory information, the data seem entirely consistent with the idea that rate remapping (and splitter cells) represent different latent states. Surely this theory (which is supported by existing data) should be referenced earlier in the manuscript, i.e. in the Results section, to situate the findings within the context of the wider

literature?
3. Regarding my third point in round 1: “Can the authors make clearer how this paper builds on previous studies that have applied optogenetic manipulations to NR to examine the effect of NR on hippocampus?..etc.” The authors provide a detailed and clear response in the rebuttal but have only added citations into the manuscript. I recommend adding into the Discussion the section (or a version of it) from the rebuttal that starts “What our study adds..”. This is to ensure that the novelty of the current results remain clear.

The fourth, fifth, sixth, seventh, eight and ninth point, together with minor comments from round 1 have been addressed. I have no additional comments for the authors.

Reviewer #2

(Remarks to the Author)
The authors have made substantial and constructive revisions, and many of my original concerns have been addressed. I have a few remaining minor points that should be clarified before publication.
Major issue: Histology and placement. I appreciate the authors’ including animal-wise histological images for illustrating probe and fiber placements in the NR, as well as channel-wise cell/spike counts based on those estimated probe channel locations, but I am still not able to see convincing evidence that the fibers and probes in question are indeed terminating in the NR. Rat 521 shows displacement of Ca1 and the dentate overlying the targeted NR, which helps in reconstructing the probe trajectory. But where the probe terminates is unclear, and in other animals, I am not able to see any disrupted tissue whatsoever. This is a crucial piece of validation for this study, for reasons explained in the first review.
The following would help with this validation:
1. Side-by-side images with and without the drawn-on putative probe location, along with detailed annotations/descriptions of which specific tissue features the authors believe indicate probe/fiber-related tissue disruption.
2. Darkfield/autofluorescence images from these slides, potentially showing tissue damage.
3. A standalone experiment using, e.g., thermolytic lesions, dye-coated fibers, etc, to demonstrate consistency in targeting the NR in the authors' hands
4. If unmounted tissue from the animals in this study is still available, IHC data showing other markers of scarring around the putative probe/fiber sites (e.g. Iba1, GFAP, NeuN, Dapi, or other markers of tissue damage)

Minor issues
1. Language regarding sequence occupancy effects. The revised analyses of NR silencing effects on pre-navigation sequence occupancy are improved, particularly with the addition of raw occupancy curves and animal-level/mixed-effects statistics. However, the effects remain relatively subtle, and some of the statistical support appears close to threshold. I would therefore suggest softening language such as “abolished” or equivalent phrasing, where present, to “reduced,” “attenuated,” or “significantly diminished.” This would better match the magnitude and robustness of the reported effects.
2. Clarification of spatial ROI/bin definitions in sequence analyses. The revised manuscript now provides a clearer description of the spatial normalization procedure used for pre-navigation sequence occupancy. However, I remain somewhat uncertain about whether the regions summarized in Fig. 2g and Fig. 5i/k were defined independently of the observed effects. In particular, if regions of interest were selected based on bins showing significant block or laser effects, then subsequent summary statistics over those same bins should be described as descriptive summaries rather than independent confirmatory tests. The authors should clarify whether these spatial windows were defined a priori, based on the task geometry, or based on the observed bin-wise effects. If the latter, the text and figure legends should make this explicit.
3. Robustness of sequence occupancy results to spatial window choice. Relatedly, because the pre-navigation sequence occupancy effects are spatially local and modest in magnitude, the authors should clarify whether the main conclusions are robust to reasonable changes in the goal-centered window definition. The added analyses improve interpretability, but a brief statement indicating that the effects are not dependent on one particular bin grouping or ROI definition would strengthen confidence in the result.
4. Pathway-level causal language. The authors have substantially improved the language regarding the mPFC->NR->CA1 pathway, particularly in the Abstract and Results. However, a few statements in the Discussion still appear to imply a more specific pathway-level causal mechanism than is directly demonstrated. The data show that mPFC and NR both represent goal-state information, and that NR activity is required for robust CA1 goal-state coding. They do not directly establish that mPFC input to NR, or serial mPFC->NR->CA1 transmission, is the source of the CA1 signal. I would suggest one additional pass through the Discussion to replace phrases such as “this prefrontal-thalamic signal” with more precise wording, for example “goal-state representations in mPFC and NR,” “NR-dependent goal-state activity,” or “prefrontal-thalamic activity involving NR.” This would preserve the authors’ model while keeping the causal claim aligned with the experiments performed.
5. Figure and legend clarity. The revised manuscript is clearer than the original, but some figure legends remain dense and difficult to parse. For panels involving shuffled controls, pseudo-block controls, or normalized spatial occupancy, the legends should explicitly state what was shuffled or normalized, what was held constant, and what statistical unit was used. This is particularly important for the new control analyses, which are valuable but will be difficult for readers to evaluate unless the logic is spelled out in the figure legends as well as in the Methods.

Overall, I view these as minor issues of clarification and calibration rather than major concerns. The revised manuscript is substantially improved, and the additional analyses address many of the central concerns raised in review.

Open Access This Peer Review File is licensed under a Creative Commons Attribution 4.0 International License, which permits use, sharing, adaptation, distribution and reproduction in any medium or format, as long as you give appropriate credit to the original author(s) and the source, provide a link to the Creative Commons license, and indicate if changes were made.
In cases where reviewers are anonymous, credit should be given to 'Anonymous Referee' and the source.
The images or other third party material in this Peer Review File are included in the article's Creative Commons license, unless indicated otherwise in a credit line to the material. If material is not included in the article's Creative Commons license and your intended use is not permitted by statutory regulation or exceeds the permitted use, you will need to obtain permission directly from the copyright holder.
To view a copy of this license, visit https://creativecommons.org/licenses/by/4.0/

We sincerely thank the reviewers for their careful reading of our manuscript and for their constructive comments and suggestions. We have revised the manuscript accordingly, and have performed a number of new analyses to address the reviewers' concerns — in particular, additional analyses clarifying the statistical robustness of our findings across animals, as well as control analyses, requested by the reviewers, that further support our main conclusions. We have also carefully reassessed the locations of all Neuropixels probes and optic fibers. Finally, we have corrected several overstatements present in the original manuscript. In our view, these revisions have substantially improved the manuscript. A point-by-point response to each reviewer's comments follows below.

Reviewer #1 (Remarks to the Author):
Summary
The authors explore how the hippocampus represents contextual information, where context is modulated by goal location along a linear track. Using electrophysiological recordings in rats they show activity in hippocampus (CA1) is segregated by context along a population dimension that is near orthogonal to spatial coding. This contextual code can also be observed in medial prefrontal cortex (mPFC) and nucleus reunions (NR). While silencing the NR does not affect task performance, it does significantly affect the population vector in CA1, which no longer shows significant difference between blocks with different vs identical goal configurations, while sparing spatial coding.
Overview: A very comprehensive manuscript with detailed analysis. Claims are largely supported by the data. A few suggestions below.

Thank you for the positive comments.

Major
1. Is the use of the term 'episode-specific' appropriate given this task? Surely 'context' would be more appropriate? Using the term episode-specific suggests that the task captures inherent features of episodic memory. Yet this task does not capture the rich what/where/when features of an episodic memory. Instead the mice are exposed to a given context for several minutes (up to 15 consecutive trials). It seems misleading to therefore use the term 'episode-specific', both in the title and throughout the Discussion.

We thank the reviewer for raising this point. By "episode," we do not intend to imply that the task captures the full what/where/when structure of episodic memory. Rather, we use the term in a broader behavioral sense, referring to a goal-defined block of task experience in which the animal repeatedly traverses the same environment under a particular goal configuration. We have removed "episode-specific" from data-specific claims throughout the Discussion and now consistently use "goal-state-specific," "goal-defined navigation episodes," or "goal-defined behavioral episodes" when referring to the neural representations identified in this task. We also considered the

alternative term "context," but believe it could be misleading in our paradigm because the external sensory and spatial context is intentionally held constant across blocks. The experimentally controlled variable is not a change in the physical environment, but an internally defined goal state associated with a goal-defined behavioral episode. We therefore retain "episode-specific" in the title and Abstract because it compactly captures the central conceptual finding that hippocampal maps are specific to these goal-defined behavioral episodes, while our revised wording makes clear that we are not claiming that the task models episodic memory in the full cognitive-psychology sense.

2. Are the hippocampal cells that discriminate between blocks 1/3 vs 2 akin to a splitter cell? If so, how are the reported findings novel? If not, what is different about this neural code and a splitter cell code?

Splitter cells, as classically defined, are characterized in trajectory-decision settings such as the T-maze, where the two options differ in both goal and trajectory (e.g., Wood et al., 2000; Frank et al., 2000). In our previous study (Ito et al., 2015), we held the goal constant while varying the trajectory and concluded that splitter-cell activity is largely attributable to differences in prospective trajectory. Importantly, splitter cells are defined only in the overlapping spatial segment that precedes the trajectory choice (i.e., the stem before the T-junction).

The present study addresses the converse situation: the maze is linear and animals have no trajectory choice, yet the goal location varies across blocks. Despite the absence of a trajectory choice, we observe rate remapping qualitatively similar to that seen in our T-maze experiments, indicating that goal differences alone, independent of trajectory, can drive rate remapping. Critically, this rate remapping is observed throughout the running phase rather than only at a decision point, generalizing the splitter-cell concept to broader spatial state coding.

A further novel contribution of the present work is extending this phenomenon to the stationary phase. Goal-state-driven activity persists even when the animal is immobile and continues to modulate prospective coding. This extends previously described goal- or trajectory-dependent running-phase activity to a more general form of persistent state coding, driven by the prefrontal–thalamic–hippocampal circuit.

The comments on the splitter cells are now described in lines 467-471.

3. Can the authors make clearer how this paper builds on previous studies that have applied optogenetic manipulations to NR to examine the effect of NR on hippocampus? Previous work (e.g. https://pubmed.ncbi.nlm.nih.gov/29940254/, https://pubmed.ncbi.nlm.nih.gov/30282726/) would suggest that the NR- hippocampal projection indeed provides goal relevant and contextual information. Can the authors articulate more clearly what is new here?

Both Maisson et al. (2018) and Ramanathan et al. (2018) establish that the nucleus reuniens (NR) is behaviorally required for tasks involving spatial working memory and contextual fear memory, respectively. The advance offered by our study lies at a different level: rather than asking whether NR contributes to memory through behavioral readouts, we characterize the specific information content that the prefrontal–NR projection conveys to the hippocampus and how it is integrated into hippocampal coding.

Maisson et al. demonstrated that silencing NR during the sampling phase of a spatial working memory task impairs choice accuracy, in a paradigm centered on the encoding and retrieval of trajectory choices. Our paradigm differs in a key respect: animals must update goal locations across blocks rather than commit to trajectory choices, since the linear maze precludes trajectory choice altogether. Consistent with this, NR silencing in our task did not impair behavioral performance, which makes direct behavioral comparison with Maisson et al. difficult. Nonetheless, the goal-state coding described here may be related to the choice-phase NR contribution they report.

Ramanathan et al. identified a related behavioral role for NR in contextual fear memory. Because their paradigm does not involve navigation or explicit goals, direct comparison is more difficult; but, like Maisson et al., their findings may reflect a broader role for NR in conveying state-related information to the hippocampus.

What our study adds beyond these works is: (i) a circuit-level characterization of how NR activity shapes hippocampal coding; (ii) direct identification of goal-state-specific neural representations across the mPFC–NR–CA1 circuit, rather than purely behavioral observations; and (iii) the demonstration that this state coding persists into the stationary phase and modulates prospective coding in the hippocampus, extending the role of NR beyond the encoding/retrieval framework examined in prior work.

We have now cited these two papers in the Discussion section (lines 519-522) where we speculate on a broader role of NR in memory encoding and retrieval.

4. Figure 3e shows that CA1 better encodes space (which we would expect), but all recorded brain regions (mPFC, CA1 and NR) encode goal location. How specific is the goal location encoding to this network of regions, or is this a brain wide code? Is there a control brain region where goal encoding is not observed?

We thank the reviewer for raising this important question. We acknowledge that the present study does not have recordings from a brain region outside the mPFC–NR–CA1 network, and we therefore cannot directly establish whether the goal-state code we describe is specific to this network or distributed more broadly. A definitive answer would require recordings from a candidate control region, for example primary sensory cortex or a non-limbic association area, and we agree this is an important direction for follow-up work. However, in the revised manuscript, we addressed whether goal encoding is uniformly distributed across the mPFC or concentrated in

specific subregions. To do so, we re-analyzed our Neuropixels recordings by sorting neurons into anatomical mPFC subregions (anterior cingulate cortex, prelimbic, and infralimbic) based on probe channel distributions and histological reconstruction, and computed goal decoding performance separately for each subregion. We found significant differences in decoding performance across subregions: in particular, decoding from neurons in the prelimbic region (corresponding to the middle range of probe channels) was significantly lower than decoding from the more dorsal (anterior cingulate) and more ventral (infralimbic) populations. This result is shown in new Extended Data Fig. 6d. This subregional heterogeneity argues against goal-state coding being a generic, brain-wide phenomenon.

5. Ln 178: which principle components explain the block and position components? If not the first 3 components then how were they selected? Seems like the block component is only explained by very small % variance?

In demixed PCA, components are demixed according to defined task variables (here, block and position), and within each marginalization they are ordered by explained variance. Each component is therefore assigned to either the block or position marginalization. We used the largest block component (block-PC1) and the two largest position components (position-PC1 and position-PC2).

The reviewer is correct that the block component captures a smaller fraction of total variance than the position components, reflecting the dominance of spatial position in firing-rate variance in CA1, mPFC, and NR during navigation. However, the absolute variance captured by a component does not directly determine decoding performance. Decoding depends on the signal-to-noise ratio of the relevant component (i.e., how consistently the same component activations recur across trials of the same condition) rather than on absolute variance magnitude. A low-variance but trial-reliable signal can support robust decoding, whereas a high-variance but trial-variable signal may not.

We have clarified this point in lines 174-178.

6. Ln 377: NR inactivation does not impair task performance. This implies that NR input to hippocampus is not necessary for the task, suggesting that task-relevant neural codes are represented in other brain regions?

The reviewer raises an important point. Our NR inactivation result indicates that the hippocampal goal-state code described here is not required for task performance on the linear maze per se, and that task-relevant codes therefore must exist outside the NR–CA1 pathway. Several lines of evidence support this interpretation.

First, in a related linear-maze paradigm (alternation between arbitrarily assigned paired goal wells), we previously showed that goal-directed navigation depends critically on the orbitofrontal cortex:

optogenetic perturbation of OFC activity specifically disrupted goal-targeting accuracy in subsequent navigation (Basu et al., Nature 2021). Second, our recent preprint shows that navigation performance in the linear maze requires dopamine release into the nucleus accumbens (Takakuwa et al., bioRxiv 2026). Third, in our companion work (Basu et al., bioRxiv 2025), animals with hippocampal lesions learned and performed the task without significant performance deficits, consistent with the present finding that NR-driven hippocampal goal coding is dispensable for the execution of this task.

Taken together, these results suggest that the immediate behavioral demands of the task (selecting and reaching the correct goal well) are supported primarily by prefrontal–striatal circuits (OFC and the midbrain dopamine–accumbens system) rather than by the hippocampus. The mPFC–NR–CA1 pathway described in this manuscript is therefore likely contributing to functions that go beyond moment-to-moment task execution. Plausible roles include mental simulation or planning of upcoming navigational paths, as demonstrated in this manuscript, which may become behaviorally relevant in more complex spatial layouts. Identifying the specific role of mPFC–NR–CA1 goal coding in navigation is an important direction for follow-up work that we now discuss explicitly in the revised manuscript (lines 457-465).

7. Figure 5i: looks like there is no effect of NR laser in two out of four rats? Why is this? Is there an effect overall across rats? What proportion of points in the upper plot come from the two rats showing an effect, vs the two rats not showing an effect?

This corresponds to Fig. 5h in the revised manuscript. We agree that the effect of NR laser inhibition appears stronger in two animals than in the other two animals. In the latter two animals, the effect was smaller and more variable (Reviewer Fig. 1). The raw session-level data for these animals are now shown separately below. In both cases, only two laser sessions were available, and the session-to-session variability was relatively large. One laser session showed a clear reduction in decoding performance, whereas the other showed a weaker or no obvious reduction. Nevertheless, both the mean and median decoding accuracies were lower during laser application than in the corresponding no-laser condition.

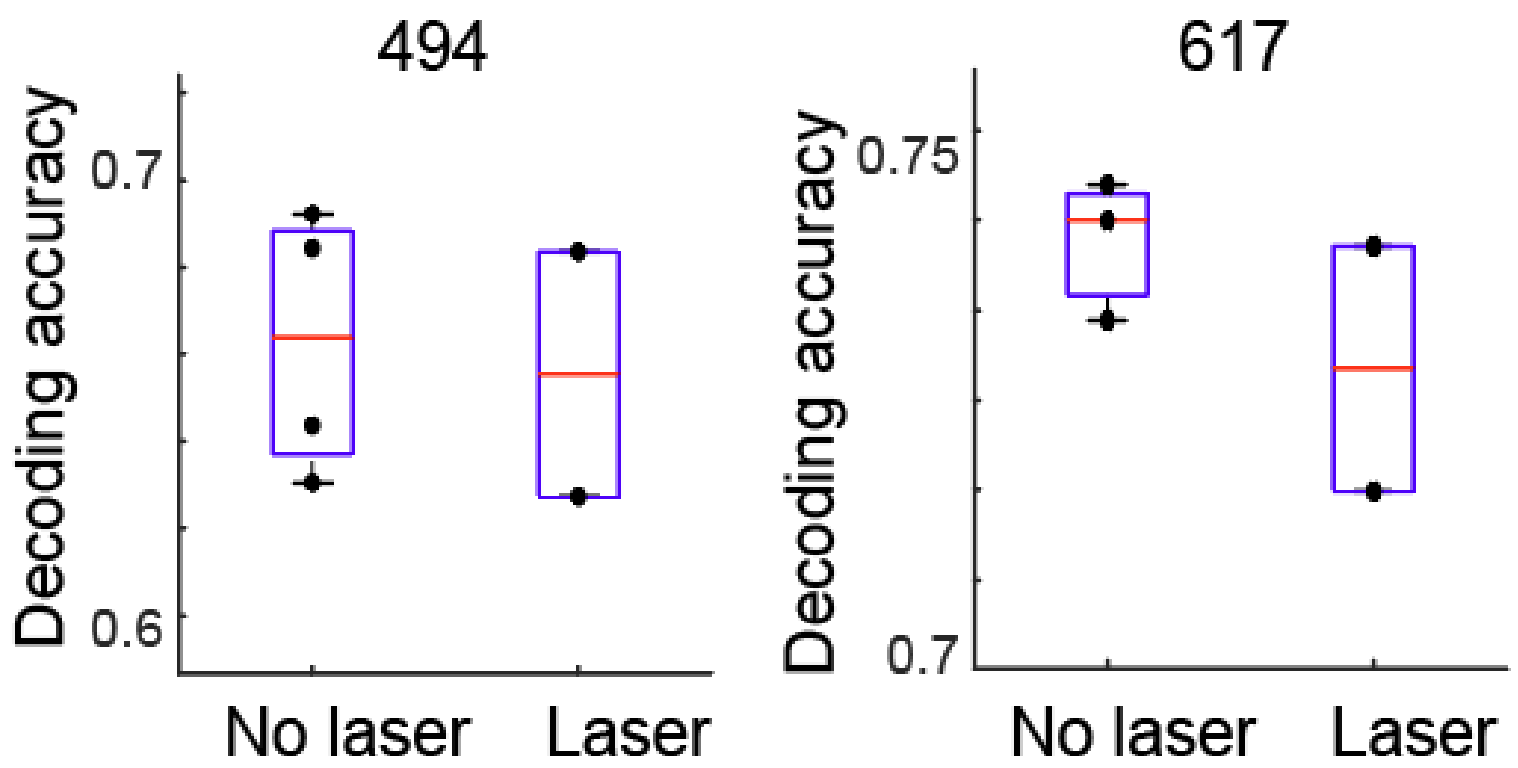

Reviewer Figure 1. Decoding accuracy in the two animals showing the smallest effects of NR silencing.

To address whether the overall effect was driven only by the two animals with larger effects, we used a linear mixed-effects model with condition no laser vs. laser as a fixed effect and animal as a random intercept. This model estimated a negative effect of laser manipulation on decoding accuracy, $\beta = -0.0716$, corresponding to an approximately 7.2 percentage-point reduction relative to the no-laser condition.

Because the number of sessions differed across animals and conditions, we further assessed statistical significance using a permutation test in which condition labels were shuffled within each animal, thereby preserving the animal-specific session structure. This analysis confirmed a significant reduction in decoding accuracy during laser application, permutation $p = 0.013$. Thus, although the magnitude of the NR laser effect varied across animals, the overall reduction in decoding accuracy remained significant after accounting for animal identity and unequal session numbers.

This point is clarified in the revised manuscript (lines 400-403).

8. Fig 5e suggests that when NR is silenced there isn't a deficit in distinguishing contexts but in recognising that two contexts are the same. Data in 5f also suggests this may be the case. This seems somewhat different to the narrative presented throughout the manuscript.

This is an important distinction, and we thank the reviewer for raising it. To address this point directly, we performed an additional analysis in which the dPCA separation between blocks was compared against the maximum separation observed across trials within the same blocks. This explicitly tests whether the expected between-block difference is significantly larger than within-block variability (Figure 5e).

The analysis shows that the separation between block 1 and block 3 is consistently not significantly different from the within-block separation, regardless of whether the laser is applied. This indicates that laser application does not enhance the separation between block 1

and block 3 (i.e., same-goal blocks), but rather reduces the separation between block 1 and block 2 (i.e., different-goal blocks). Under laser application, hippocampal population activity therefore fails to discriminate between blocks with distinct goals, rather than over-discriminating between blocks with the same goal.

This interpretation is further supported by the reduced proportion of rate-remapping place cells observed under NR silencing (Extended Data Fig. 9).

We have clarified this point in the revised manuscript (lines 388-391).

9. Fig5j: how are these windows for comparison chosen without introducing a multiple comparisons problem?

This corresponds to Fig. 5i in the revised manuscript. The analysis in Figure was designed to quantify the maintenance of event occupancy toward the upcoming goal location, paralleling the analysis in Fig. 2e. We computed the ratio of event occupancy at the upcoming goal location to that at the midpoint of the journey, providing a single scalar summary of how event occupancy evolves over the second half of the trajectory. Because goal locations varied across sessions, defining this window relative to the upcoming goal provided a common reference frame for cross-animal comparison.

This analysis constitutes a single pre-specified comparison testing one hypothesis, whether event occupancy is maintained toward the upcoming goal, rather than an exploratory scan across candidate windows. Under no-laser conditions, occupancy was sustained across the window; under laser, it was significantly reduced. Because a single planned test of a single, pre-specified window was performed, no multiple-comparisons correction applies.

We have clarified this in the revised manuscript (Fig. 5i).

Minor

1. ln 310-311 may want to edit this statement to say 'in this task, population activity in mPFC and NR primarily functions…' as there are of course other primary functions of these brain regions which cannot be revealed by this task.

We agree that the original wording could be interpreted too broadly, as this task does not assess all possible functions of mPFC or NR. We have therefore revised the sentence to make the conclusion explicitly task-specific: "Together, these results show that mPFC and NR encode goal-related information comparably to CA1, whereas their representation of spatial position is substantially weaker. This pattern is consistent with the view that activity in the mPFC–NR pathway contributes to the hippocampal goal-state signal, which is integrated with spatial information in CA1 to form spatially aligned, goal-specific population representations."

2. Ln 334: not clear what is being trained and tested? What is meant by goal patterns being reliably decoded? Do they train on goal vs not goal location and then test on held out data samples? How are these not simply spatial representations?

The construction of the goal-state decoder is illustrated in Fig. 3a. The "goal patterns" refer to the animal's currently engaged goal configuration: one configuration was used in blocks 1 and 3, and a different configuration in block 2. The classifier was trained to decode which goal configuration the animal was currently engaged in, given population activity at any time point during the session.

Training was performed on balanced trials sampled across blocks 1–3, and the decoder was tested on held-out trials. The classes being decoded are the active goal configurations (e.g., 1-4-10 vs. 1-7-10), not spatial positions. Critically, animals traverse the same spatial trajectory in all blocks, and therefore, the decoder cannot be reduced to a spatial classifier.

We have clarified this point in the revised manuscript (lines 305-311).

3. Figure 2h is not clear: what is being shown and which statistical test do the red stars refer to?

The plot is essentially the same as Fig. 2g and is intended to show the session-wise and animal-wise tendency of the effect, demonstrating that the same trend (i.e., change in pre-navigation sequences) is observed across sessions and animals. The statistical test is the Wilcoxon signed-rank test. We have concluded that this plot is not essential for the main figure and have moved it to Extended Data Fig. 3k.

4. Figure 4c and 4g: while the results look compelling, would it not be more robust to compare the group mean of the data against a null distribution generated using permutation tests, with >1k shuffles?

The presented chance level was estimated using a permutation-based procedure. However, rather than shuffling class labels, which produces decoding performance near 0.5 and is a relatively weak control, we adopted a more conservative null in which the transition points between goal configurations were assigned arbitrarily (Extended Data Fig. 7a). This null captures how well the classifier could distinguish arbitrary temporal structure in the population activity (e.g., arbitrary classification due to representational drift of neural activity) rather than true goal-related changes. The null was computed per session by 1000 iterations, yielding one averaged chance value per session and matching the sample size of the observed data.

We found that the observed decoding accuracy was significantly higher than this per-session chance level in both Fig. 4c and 4g. This indicates that the decoder performed better than would be expected from non-goal-related temporal structure such as drift alone. We have now clarified this permutation procedure in the text.

5. In Figure 5f, left hand panels, is there a significant interaction between on and off laser? An interaction is shown in right hand panel but with a different y axis label.

Yes. Because the No-laser and Laser values were paired within sessions, we performed a repeated-measures ANOVA and tested the interaction by comparing the paired effect sizes between conditions. This analysis revealed a significant interaction ($p = 0.038$). We have now clarified the statistical test and corrected the y-axis label in the right-hand panel to indicate that it shows the interaction contrast. We have edited the manuscript and the figure (lines 400-403 and Fig. 5e).

6. Do the authors distinguish between the pyramidal neurons and interneurons in their analysis? Why are putative interneurons included, for example in Fig1c, Rat617, Cell146 looks like an interneuron.

We included these representative examples because putative interneurons contribute substantially to the persistent goal-state signal in CA1. This has been shown in the Extended Data Fig. 3g and 7f. We first confirmed that classic rate-remapping is observed in putative interneurons. Regarding the goal-state coding, we separated spatial and nonspatial cells and both types of neurons show robust goal-state coding. This is biologically meaningful: most CA1 pyramidal cells are strongly position-selective and modulated by locomotion state, whereas a persistent goal-state representation that generalizes across positions and behavioral states is naturally supported by cells with broader spatial tuning. Including interneurons in the analysis was therefore a deliberate choice. This point is now discussed in lines 479-482.

Reviewer #2 (Remarks to the Author):

This is a thoughtful and technically sophisticated manuscript that addresses how hippocampal population activity can preserve spatial information while also distinguishing different goal-defined navigation episodes. Using a well-controlled A/B/A goal-switching design, large-scale recordings from CA1, mPFC, and nucleus reuniens, population-level decoding/dPCA analyses, and optogenetic NR perturbation, the authors present evidence that CA1 encodes goal or task-context information in a dimension that is largely separable from position coding. The study has several notable strengths: the behavioral design allows the same spatial environment to be examined under different goal configurations; the analyses attempt to dissociate spatial coding, goal-state coding, and pre-navigation sequence content; and the NR silencing experiment provides a potentially important causal link between thalamic activity and CA1 goal-context representations. The combination of hippocampal population geometry, pre-run sequence analysis, and circuit perturbation makes the work potentially significant. However, several aspects of the analysis and interpretation require clarification or additional controls before the strongest claims are fully supported. In particular, the manuscript would benefit from more rigorous anatomical validation of recording/manipulation sites, clearer definitions of the spatial binning and normalization used in sequence-occupancy analyses, stronger null models for the orthogonality and cross-block separation claims, and more conservative language regarding mPFC–NR–CA1 pathway causality.

1. The manuscript shows Nissl-stained sections with tetrode and optic fiber placements, and Fig. 3a appears to show representative Neuropixels tracks in mPFC and NR. However, the manuscript does not clearly provide systematic reconstructions of Neuropixels recording sites across animals, including the estimated locations of recorded units along the probe trajectory. This is particularly important for NR recordings, given the small sample sizes used here and the proximity of neighboring thalamic and hypothalamic nuclei. Authors should provide atlas-registered probe tracks and estimated recording-site locations for each Neuropixels animal/session, together with criteria for assigning units to mPFC or NR and excluding off-target recordings.

We agree this is an important addition and have now provided systematic per-animal reconstructions.

For each animal, probe tracks were reconstructed from Nissl-stained sections, and the boundaries of NR were defined directly from cytoarchitecture using the Paxinos & Watson atlas as an orientation reference. The reconstructed track and the inferred location of each recording channel are shown for every animal in revised Extended Data Fig. 4.

Units were assigned to NR or mPFC based on the probe channel on which the unit's spike amplitude was maximal. For NR recordings, we restricted analysis to the 150 channels closest to the probe tip, which fall within the Nissl-defined boundaries of NR; channels dorsal to this region

were excluded. For mPFC recordings, histological confirmation indicated that all 384 channels of the probe fell within anterior cingulate, prelimbic, or infralimbic cortex, and all were therefore included. Revised Extended Data Fig. 4 also shows, for each animal, the histogram of included units along the probe for both NR and mPFC recordings, confirming that recorded units are concentrated within the target regions.

2. Similarly, the Nissl-stained sections shown in Extended Data Figure 2 are intended to show optical fiber tracks. However, despite the visual annotation, the fiber tracks themselves are not visually discernible. This is particularly important in this study, because the fluorescent histological sections accompanying these show strong expression in the hypothalamus, in some cases stronger than in NR. If the histological images themselves, without the estimated fiber locations overlaid, cannot confirm fiber placement, then a separate experiment, in which fibers placement is confirmed using dye-coated fibers or thermolytic lesioning, is in order.

We agree that this is an important concern and address fiber placement and viral spread separately.

Fiber tip location. Revised Extended Data Fig. 2 now provides higher-magnification Nissl images for each animal with fiber tip positions marked. Across all 4 animals, fiber tips were localized within NR.

Viral spread and off-target silencing. AAV expression extended ventrally into hypothalamic regions in all animals, and this warrants careful evaluation. Three complementary lines of evidence indicate that the reported effects are unlikely to be explained by off-target silencing.

First, a biophysical argument. We used tapered Lambda fibers (Optogenix; 400 µm fiber, 1 mm taper, 0.39 NA), whose emission geometry differs fundamentally from that of flat-cleaved fibers. Optogenix provides a light-propagation simulator based on the Monte Carlo model of Stujenske et al. (2015). Although our exact custom geometry could not be simulated directly, simulations are available for the closest related configuration (200 µm fiber, 1 mm taper, 0.39 NA). Because the 400 µm fiber's emission area is approximately four times that of the 200 µm fiber, a 200 µm simulation at 5 mW yields irradiance values comparable to those produced by our 400 µm fiber at 20 mW, and is therefore informative for our configuration (Reviewer Fig. 2). The power distributions differ markedly: a flat-cleaved fiber concentrates emission below the tip and maintains ≥ 3 mW/mm² (the irradiance used by Berndt et al. (2016) to characterize SwiChR++ kinetics) up to ~1.35 mm ventral to the tip, whereas the tapered Lambda fiber distributes most power laterally along the taper and drops below 3 mW/mm² within ~0.3 mm ventral to the tip. The geometry of the Lambda fiber therefore strongly restricts ventral light spread into hypothalamic territory.

Second, an anatomical argument. The hypothalamic regions within the spread zone — dorsal and dorsomedial hypothalamus — provide only sparse projections, if any, to the hippocampal

formation (Swanson, 1976; Wyss et al., 1979; Vertes et al., 2007). Off-target silencing of these regions is therefore unlikely to account for the hippocampal effects we report.

Third, a neural-coding argument. The manuscript provides converging evidence that goal-state coding is expressed within NR itself and within its major afferent and efferent partners (mPFC and CA1), consistent with a circuit-level effect originating in NR rather than in hypothalamic structures lacking the relevant connectivity.

Together, the consistency of the effect across 4 animals, combined with the biophysical, anatomical, and circuit-level considerations above, supports the interpretation that the manipulation acts primarily through NR. We have added this limitation explicitly to the legend of Extended Data Fig. 2.

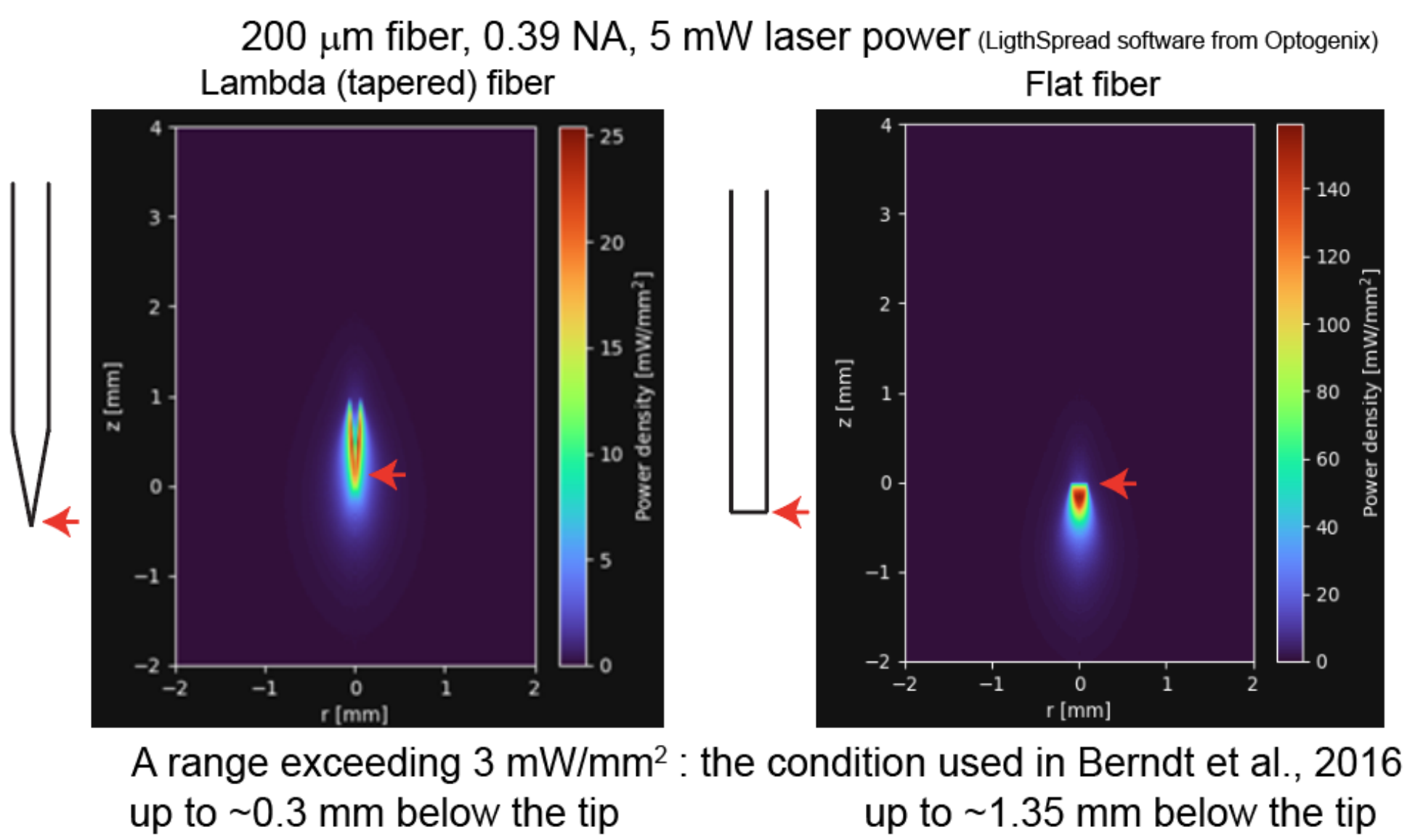

Reviewer Figure 2. Simulated light spread from a Lambda-B fiber.

Wyss, J. M., Swanson, L. W. & Cowan, W. M. A study of subcortical afferents to the hippocampal formation in the rat. *Neuroscience* **4**, 463-476 (1979).

Swanson, L. W. An autoradiographic study of the efferent connections of the preoptic region in the rat. *J Comp Neurol* **167**, 227-256 (1976).

Vertes, R. P., Hoover, W. B., Szigeti-Buck, K. & Leranth, C. Nucleus reuniens of the midline thalamus: link between the medial prefrontal cortex and the hippocampus. *Brain Res Bull* **71**, 601-609 (2007).

3. Figure 1g shows similar decoding accuracy with blockwise vs general decoding, but this may not be the most sensitive way of detecting spatial coding differences across blocks. This result is used to substantiate the claim that location coding is unchanged across blocks or goal states. A

more direct test would be to see decoders trained on each block and tested on each other block. The higher population vector correlation between blocks 1 and 3 than between 1 and 2 (EDF 3g) makes this especially important to show.

Following the reviewer's suggestion, we performed cross-block Bayesian position decoding: decoders were trained on data from each block and tested on the other two blocks (new Extended Data Fig. 3e). Cross-block decoding accuracy was uniformly high across all train/test pairs and did not differ significantly across training blocks (Kruskal-Wallis, $p = 0.096$).

This apparent dissociation between PV correlation and decoding accuracy is expected: Bayesian decoding relies primarily on the spatial location of place-field peaks, whereas population vector correlation is additionally sensitive to changes in absolute firing rate (rate remapping) that do not shift place-field locations. Stable place-field positions across blocks therefore support stable cross-block decoding even when firing rates (and consequently PV correlations) vary across blocks.

4. Extended Data Figure 3h presents a finding of 'nearly-orthogonal' spatial and goal components – but orthogonal relative to what null distribution? This is a concern in high-dimensional state space, and derivations of such a space, which tends to drive vector angles toward orthogonality.

We thank the reviewer for raising this point. We address the two components of the concern in turn.

First is about what was meant by "nearly orthogonal" in Extended Data Fig. 3h (now 3i). Unlike standard PCA, dPCA does not enforce orthogonality between marginalization components by construction, but it nonetheless tends to produce near-orthogonal axes in any high-dimensional dataset. We confirmed this by applying dPCA to shuffled data, which yielded angles comparable to those in the unshuffled data (~85°). In the original manuscript, we used "nearly orthogonal" only to justify the forced orthogonalization applied for visualization in Fig. 1j, not as a substantive geometric claim. We have revised the corresponding text.

Second, we performed a direct test against a null distribution. To formally test the geometric relationship between coding axes, we used linear discriminant analysis (LDA) to identify the subspaces that maximally discriminate goal configurations and spatial positions, and computed the principal angle between these subspaces. We compared the empirical angle to a null distribution generated by shuffling goal/position labels ($n = 100$ shuffles per session). The empirical angle was significantly larger than the null (empirical median = 61.2°; shuffled median = 35.6°; $p < 0.001$ in the Kolmogorov-Smirnov test; new Extended Data Fig. 6e).

While this test confirms that the spatial and goal subspaces are more orthogonal than expected by chance, we agree with the reviewer that strict mathematical orthogonality is difficult to substantiate in high-dimensional space. For this reason, our central claim is framed in terms of information separability between subspaces rather than geometric orthogonality per se: goal information can be decoded from the goal subspace but is lost when neural activity is projected onto the spatial subspace, and the reverse holds for spatial information (e.g., Extended Data Fig.

3j). This functional separability supports our interpretation that goal and spatial information are carried by largely independent population subspaces, independently of the precise geometric angle between them.

5. Figure 1j is presented in arbitrary units. To make this result interpretable, a null comparator should be presented using a position- and direction- matched shuffle across blocks. If enough trials worth of data are available, it would also be good to see a block-to-self (e.g. B1-B1, B2-B2, and B3-B3) comparison, to determine the ceiling for this axis separation effect. Figure 1k contains a Shuffled condition, but it is not clear how this shuffle was performed, i.e. whether position and direction were held constant across blocks.

We thank the reviewer for these suggestions. We have performed both additional analyses and clarified the shuffle procedure.

Position- and direction-matched null comparator. To generate the null, we shuffled block labels within matched position and running-direction bins. The original dPCA axes were retained, and the block-axis separation was recomputed on the shuffled data. Under this null, block-axis separation difference collapsed to near zero (mean shuffled separation = 1.12 ± 0.11, vs. empirical separation: B1-B2 = 3.73 ± 0.29, B1-B3 = 1.91 ± 0.28; new Extended Data Fig. 3i), confirming that the original separation reflects genuine block-related structure.

Block-to-self ceiling. As a ceiling for the axis-separation measure, we computed separation between trials within the same block (B1-B1, B2-B2, B3-B3) using identical methodology (Fig. 1j). The maximum within-block separation was not significantly different from the Block 1-3 separation ($p = 0.145$), but was significantly smaller than the Block 1-2 separations ($p < 0.001$). This result is consistent with our experimental design, in which Blocks 1 and 3 share the same goal configuration while Block 2 introduces a different one. The fact that B1-B3 separation reaches only the within-block ceiling, rather than the larger separation seen with B2, confirms that the axis-separation measure captures genuine differences in goal state rather than session-to-session drift.

Shuffle procedure for Fig. 1k. The shuffle was a class-label shuffle of the decoder, in which decoder class labels (block identity) were permuted while position and direction were held constant. We have added this clarification to the figure legend and Methods.

6. The cross-block claims would be bolstered by some additional controls for temporal drift. CA1 population activity drifts over time, and an A/B/A design helps but does not completely eliminate temporal confounds. A useful temporal-drift control would be to assign pseudo-blocks of matched duration by circularly shifting block boundaries or placing pseudo-boundaries within blocks, then recomputing, e.g., dPCA separation. The observed goal-block separation should exceed this pseudo-block null.

We have addressed the temporal-drift confound with the pseudo-block analyses suggested by the reviewer, and we provide additional direct evidence that drift in our dataset is geometrically orthogonal to the goal-coding axis.

First, we computed a pseudo-block null by placing pseudo-boundaries within each block, generating pseudo-blocks that share the same goal configuration but differ only in temporal segment (Extended Data Fig. 7a). This null serves as the chance level for goal-state decoding accuracy (see also our response to point 10) and is more stringent than simple class-label shuffling, which yields values near 0.5. The genuine goal-state decoding accuracy clearly exceeds this pseudo-block null, confirming that goal-state coding cannot be explained by temporal drift.

Second, we applied the same pseudo-block procedure to the dPCA block-separation analysis (Extended Data Fig. 7a). For pseudo-blocks, the separation between blocks 1 and 3 was significantly larger than between blocks 1 and 2, which is the expected signature of temporal drift, separating segments in proportion to their temporal distance. This pattern is opposite to the one observed for genuine goal blocks, where the order of separation reflects goal-state similarity rather than elapsed time, indicating that the dPCA separation we report is driven by goal-state information rather than drift.

We also note that CA1 activity does carry information about the temporal sequence of blocks, which we explicitly characterize in Extended Data Fig. 10i–k. Block sequence could be decoded from CA1 activity significantly better than within-block arbitrary temporal segments. Importantly, this sequence-coding axis is nearly orthogonal to the goal-coding axis (mean angle = 84.7°), so block-sequence information resides in a subspace distinct from the goal-coding readout. In the revised manuscript, we further show that orthogonal subspaces can be constructed that primarily encode either goal configuration or block sequence (Extended Data Fig. 10j). Together, these analyses establish that goal decoding operates in a subspace geometrically separated from both the within-block drift and the block-sequence dimensions, and that the goal-block separation we report reflects genuinely goal-specific information.

7. The effect shown in 2e is subtle, and I am therefore concerned with how the spatial bins are grouped for analysis. The authors describe a process of some sort of spatial normalization (Line 759, "The time occupancies of sequence representations were estimated at every 2.5 cm bin for each recording session and normalized for the distances of the middle or trackend goal locations"), but it is not clear what is meant by this. The regions delineated for comparison in Figure 2e are described as 'quadrants' – how were these regions defined, precisely? The regions annotated in the trackend comparison (2e right) look smaller than the 'quadrants' in 2e left – are they? Why did the authors switch from quadrants to 'regions of interest' for the pre-running sequence analysis (2f), and how were these location sizes determined? Then once again they switch back to another definition for spatial location groupings for Figure 5 ("...ratio of occupancy near the upcoming

goal…”). It would be helpful to show that these effects are robust to changes in the spatial groupings.

We thank the reviewer for highlighting these confusing parts. We have revised the manuscript to address each point.

First we want to clarify the reason of spatial normalization (Fig. 2e). The plot in Fig. 2e is based on normalized rather than absolute position. This step is necessary because goal configurations differ across sessions and blocks (e.g., from well 1 to well 4, 5, 6, or 7), and combining trials is required to evaluate goal-specific trends. We therefore first computed event density at 2.5 cm bins along the actual track and then linearly interpolated this density onto a normalized axis spanning the start well to the middle goal. A second, independent normalization was applied between the middle goal and the trackend goal. We have rewritten the Methods to state this explicitly.

Definition of quadrants (Fig. 2e). The quadrants were designed to quantify a specific feature of the density distribution: event density appears to be sustained along the immediate future path from the start to the goal, then drops sharply beyond the goal. Our aim is to test this hypothesis statistically. However, because event density naturally decays with distance from the animal's current position, we need to disentangle this baseline decay from the goal-related modulation. This is the reason why a ratio of the density at different spatial references is computed to distinguish the goal-related component from this baseline decay. The quadrants divide the normalized axis into equal segments before and after the goal, allowing us to compute a pre-goal vs. post-goal decay. If sequence representations are maintained up to the goal, the pre-goal decay is shallower than the post-goal decay.

The comparison of the future and past goals in Fig. 2e right is more straightforward. We wanted to compare the density around the future and past goal spatial references, which are defined as corresponding quadrants on the same normalized axis. We have clarified this in the figure legend.

Switch to regions of interest (Fig. 2f) and Fig. 5. In Fig. 2f and Fig. 5, the comparison can be performed between blocks with different goal locations (proximal vs. distal goals) rather than within a single distribution. This shifts the appropriate statistical approach: rather than defining quadrants a priori, we performed spatial-reference-wise statistical comparisons across the normalized position axis and highlighted the regions where blocks differed significantly (shown as the black lines). No arbitrary location sizes were imposed as these regions emerge from the statistics themselves. For Fig. 2g, our aim was to show the trial-by-trial trend underlying the block-level effect in Fig. 2f. We therefore averaged event density across the 15 normalized bins immediately preceding the proximal goal — a window chosen to encompass the contiguous region identified as significant in Fig. 2f. The underlying normalization for this analysis divided each trial into three segments: 50 bins from the start to the proximal goal, 25 bins from the proximal to the distal goal, and 50 bins from the distal goal to the trackend. Fig. 5 uses the identical procedure. We have added these details to the Methods and unified the wording across figures so that the analysis pipeline is described consistently throughout.

Robustness. We agree that demonstrating robustness to the choice of grouping is important. In the following Reviewer Figure 3, we now show that the Fig. 2e effect is preserved across a range of slope estimation, which is contrasted with a simulated monotonically decaying distribution. The block comparisons in Figs. 2f and 5 do not depend on grouping choices, since they are computed bin-wise.

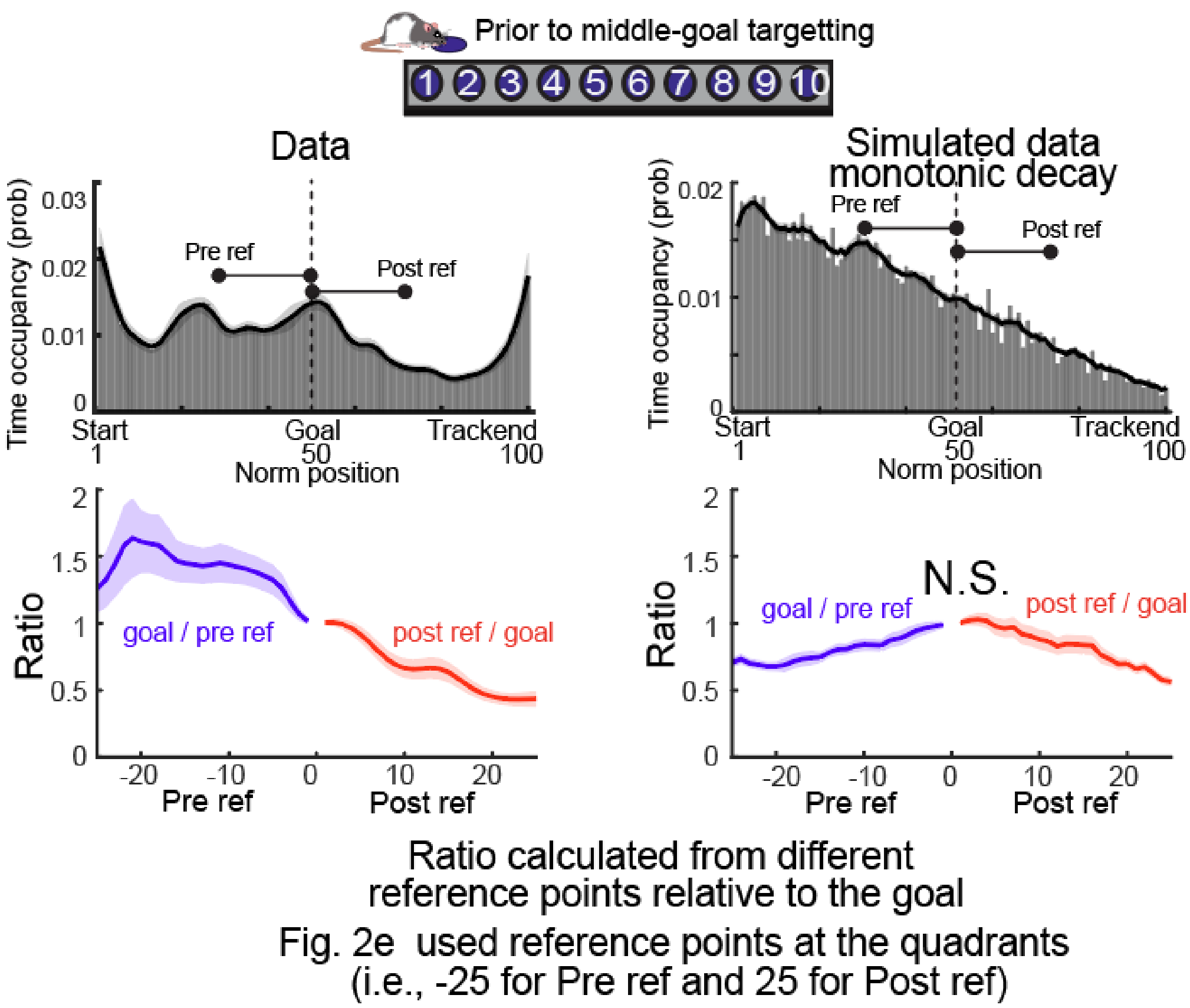

Reviewer Figure 3. Robustness of the pre-navigation spike sequence ratio to the choice of reference point.

8. I had trouble interpreting Extended Data Figure 3k. What does it mean to have a probability >1 for 'slope to goal'? if the y-axis is mean to somehow denote the slope of the sequence occupancy trace before and after goal approaches, it is still not clear why slope and not magnitude is the relevant metric here, or over what set of spatial bins this slope was computed.

We apologize for the confusing labels. This plot is closely related to point 7 above: for each animal, it shows the event occupancy ratio computed separately for the before-goal and after-goal segments of the journey. In the before-goal segment, a ratio significantly greater than 1 indicates that occupancy is sustained as the animal approaches the goal; in the after-goal segment, a ratio less than 1 indicates that occupancy drops more steeply once the goal is passed. We have relabeled

the y-axis as "Ratio" and the x-axis categories as "Before goal" and "After goal", and revised the legend accordingly.

Why slope rather than magnitude. Occupancy probability naturally decays with distance from the animal's current position regardless of goal structure, so the absolute magnitude at any point in space conflates two sources of decay: the natural distance-dependent fall-off and any goal-related modulation. The slope ratio isolates the goal-related component by asking whether the decay significantly changes at the goal location. We illustrate this logic with a control simulation (Reviewer Fig. 3), in which we generate distributions with monotonic, distance-dependent decay; the slope-ratio metric consistently remains below 1, confirming that the change of values from large to small at the goal position observed in our data cannot be explained by baseline decay alone.

Bins used for the slope calculation. Ratios were now computed over different reference points (Reviewer Fig. 3). The results are robust across different choices of reference points, confirming the change near the goal location is robustly significant across bin choices.

9. 2g only shows occupancy for distal goal in proximal-first sessions, and for the proximal-first goal in distal-first sessions. it would be helpful to see occupancy decline in the regions associated with block 1, as occupancy shifts toward the block 2 goal during block 2.

We agree this is an important complementary analysis and have now performed it in the new Extended Data Fig. 3l.

The result is informative. During block 2, the sequence density at the block 1 goal location was not reduced relative to its block 1 level, even as new goal-specific density emerged at the block 2 goal. Pre-navigation sequences therefore appear to add a representation of the new goal rather than replace the representation of the previous one. In contrast, during block 3, the sequence density that had appeared at the block 2 goal was reduced, while the original block 1 representation remained intact.

These observations suggest an asymmetry in how goal representations are updated: the representation acquired in the first block is robustly maintained, while representations acquired in subsequent blocks are more labile and can be attenuated when a further goal change is introduced. This is consistent with a memory system in which the initially established representation occupies a privileged state, and later representations are layered on more transiently. We are cautious about over-interpreting this asymmetry, since it may depend on features of our task structure (e.g., the specific block ordering and the longer cumulative exposure to the block 1 goal); a more systematic dissection would require manipulations of block order and duration, which we view as an important direction for follow-up work. We have added this analysis and a brief interpretation along these lines to the Extended Data figure legend.

10. In Extended Data Fig. 4, the authors should explain why chance decoding is above 0.5 for all three regions – is this a result of trial number imbalance across conditions?

We believe the reviewer is referring to Extended Data Fig. 7a (rather than Fig. 4). The chance values above 0.5 do not reflect trial number imbalance. Rather, the chance level in this panel was deliberately computed by randomly reassigning time segments *within the same goal block*, rather than by simple class label shuffling (which yield chance values near 0.5). We adopted this more stringent procedure, which is relevant to the reviewer's related concern (point 6) about whether slow temporal drifts, independent of goal-state specificity, could account for the observed decoding. The fact that goal decoding accuracy significantly exceeds it thus demonstrates that our results cannot be explained by drift alone.

11. In several places, the manuscript uses language that appears stronger than what is directly supported by the presented data. For example, line 310-311 states: "population activity in mPFC and NR primarily functions to segregate navigation episodes associated with different goal patterns". This claim overstates what can is demonstrated here, as these results do not establish what the "primary functions" of mPFC and/or NR are during this task. What is supported by Figure 3 is that population activity in both structures is associated with goal states more reliably than with location.

We agree that this task does not establish the full functional repertoire of mPFC or NR. We have therefore revised the sentence to make the conclusion explicitly task-specific: "Together, these results show that mPFC and NR encode goal-related information comparably to CA1, whereas their representation of spatial position is substantially weaker. This pattern is consistent with mPFC and NR providing a goal-state signal that is integrated with spatial information in CA1 to form spatially aligned, goal-specific population representations, an interpretation further supported by the NR-silencing results in Figure 5." We applied analogous revisions to other instances of overstated language elsewhere in the manuscript (see our response to point 12).

12. Another overstated claim, which is repeated throughout, is that the orthogonal goal-state representation observed in HPC is transmitted from mPFC to NR to HPC. For example, the abstract says: "This goal-state signal is supplied by a prefrontal–thalamic pathway, in which population activity in medial prefrontal cortex and nucleus reuniens forms persistent representations". Similarly, in the intro (line 77): "…we examine how goal-related information is causally transmitted to the hippocampus". And in line 441: "In this study, we show that prefrontal top-down input routed through the mPFC–NR–CA1 pathway enables the hippocampus to distinguish distinct navigation episodes…" What the authors have demonstrated instead is a correlation between the observed orthogonal goal-state signal in HPC and mPFC and NR activity,

combined with causal evidence that NR activity is critical for the HPC representation. This does not rule out the possibility that the goal state signal is transmitted via a broader circuit.

We thank the reviewer for this important point. Our data demonstrate (i) correlated goal-state representations across mPFC, NR, and CA1, and (ii) a causal requirement for NR in the CA1 goal-state signal, but do not establish that the relevant signal is transmitted specifically along mPFC → NR → CA1. We have revised the claims throughout the manuscript to reflect this distinction, replacing assertions of directional transmission with statements about correlated activity and the causal necessity of NR. The key changes are below.

Abstract. "This goal-state signal is supplied by a prefrontal–thalamic pathway, in which population activity in medial prefrontal cortex and nucleus reuniens forms persistent representations" has been revised to:

"This goal-state signal is also represented in the medial prefrontal cortex (mPFC) and nucleus reuniens (NR), where population activity forms persistent representations across locomotion and immobility and is reliably reinstated when previously experienced goal configurations recur."

Introduction. "…we examine how goal-related information is causally transmitted to the hippocampus" has been revised to:

"…we examined how goal-related information is incorporated into the population coding structure of each region and how it shapes goal-state-specific hippocampal maps."

Discussion. "In this study, we show that prefrontal top-down input routed through the mPFC–NR–CA1 pathway enables the hippocampus to distinguish distinct navigation episodes…" has been revised to:

"In this study, we show that population activity in mPFC, NR, and CA1 carries goal-state representations that distinguish navigation episodes within an identical spatial context, and that inactivation of NR significantly reduces the hippocampal goal-state signal."

We have also reviewed the manuscript for similar phrasing elsewhere and applied analogous corrections.

13. The authors should report first-order effects of NR silencing on CA1 activity, including mean/median firing rates, firing-rate distributions across neurons, fraction of active cells, and burst statistics if available. This is important for distinguishing a selective disruption of goal-state coding from a more general change in CA1 excitability, gain, or network state. The rate-remapping analyses appear to quantify unsigned changes in firing rate. It is therefore unclear whether goal-related rate remapping reflects a balanced redistribution of activity across CA1 cells, a global increase/decrease in CA1 firing in block 2, or selective modulation of positively versus negatively tuned neurons. The authors should report signed firing-rate changes across blocks, including the

fractions of cells increasing versus decreasing from block 1 to block 2, and test whether NR silencing preferentially affects either sign of modulation.

We have performed these analyses to characterize first-order effects of NR silencing on CA1 activity.

First, we examined mean firing rates of CA1 neurons (restricted to place cells) and found no significant difference between laser-on and laser-off conditions ($p = 0.883$ in the Kolmogorov–Smirnov test). Burst rate showed a non-significant trend in the same direction ($p = 0.060$).

Second, to address signed rate changes across blocks, we asked for each rate-remapping neuron whether its firing rate was higher in blocks 1 and 3 or in block 2. Approximately 60% of rate-remapping cells showed higher rates in blocks 1 and 3, indicating a modest baseline asymmetry in the population. Critically, this proportion was not significantly altered by laser application ($p = 0.739$ in the Wilcoxon rank-sum test), indicating that NR silencing does not preferentially affect positively versus negatively tuned cells.

Together, these analyses reveal no significant first-order effect of NR silencing on CA1 excitability or on the sign of rate modulation, supporting our interpretation that the observed deficit reflects a selective disruption of goal-state coding rather than a global change in CA1 firing. We note one caveat: laser-on and laser-off sessions were performed on different days, so the same units could not be tracked across conditions. This precludes within-cell comparisons and may reduce sensitivity to subtle first-order effects.

14. The NR inhibition effects on goal occupancy in the pre-running sequences are presented as a probability ratio (Figure 5j). The interpretation would be strengthened by presenting these ratios alongside raw occupancy curves with confidence intervals, rather than the ratios alone. It would also be helpful to see animal-level statistics, a mixed-effects model with session nested within animal, and permutation/bootstrap controls.

We thank the reviewer for this helpful suggestion, which addresses several complementary aspects of the analysis.

Raw occupancy curves. We now present the underlying occupancy curves for each animal (new Extended Data Fig. 10g), allowing the effect of NR inhibition to be assessed directly from the underlying distributions rather than from the normalized ratio alone.

Mixed-effects analysis. We fit a linear mixed-effects model treating each session as an observation, with animal identity as a random intercept to account for the repeated-sessions-per-animal structure of the data. The effect of NR inhibition on the goal-occupancy ratio remained significant after accounting for between-animal variability ($F(1,22) = 4.48$, $p = 0.046$).

Non-parametric sensitivity check. As a distribution-free control, we additionally performed a within-animal permutation test (10,000 iterations) on the same linear mixed-effects model. On each iteration, condition labels were shuffled across trials within each animal, and the model was refit to generate a null distribution for the fixed-effect coefficient. The two-tailed permutation p-value was $p = 0.044$, closely matching the parametric result and supporting the robustness of the effect to distributional assumptions.

Minor issues:

1. The manuscript relies heavily on findings presented in Extended Data figures, but references to these figures leave out the figure panel, citing only the figure number. This makes it difficult to discern what evidence is being used to support what claim. References to extended data figures should cite which panel is being used to substantiate each specific claim.

We have included them in the revised manuscript.

2. Typos: Extended data figure 3g: "Population-vector correstions"

Thank you for indicating this point. We fixed it.

3. Some of the figure panels are out of order – e.g. 5e and 5f

We corrected it.

We sincerely thank the reviewers for their further constructive comments and suggestions. We revised the manuscript to use more precise terminology, expand the relevant discussion, and strengthen the histological validation of Neuropixels probe and optic fiber placements. We believe these revisions have substantially improved the manuscript. Our point-by-point responses follow.

Reviewer #1 (Remarks to the Author):

While the authors have made a number of changes to the manuscript, three of the major points I raised in round 1 have not been addressed. I therefore have outstanding comments which I list below.

1. Regarding my first point in round 1: "Is the term "episode-specific" appropriate given the task? Etc…"
The authors have not addressed my concern that the term "episode-specific" in the title is misleading (point 1 of my previous review). So called "episodes" are actually multiple trials in a particular context, while use the term "episode-specific" implies that the task and analyses make claims about episodic memory, which is not the case.

We thank the reviewer for reiterating this concern. We have revised the title by removing the term "episode-specific," thereby avoiding the implication that the task directly examines episodic memory.

2. Regarding my second point in round 1: "Are the hippocampal cells that discriminate between blocks 1/3 vs 2 akin to a splitter cell? If so, how are the reported findings novel? If not, what is different about this neural code and a splitter cell code?"
The authors additional reference to splitter cells in the revised Discussion section doesn't account for theories that would encompass the data presented in the paper. In particular, splitter cells may reflect the underlying latent state or temporal context (https://elifesciences.org/articles/82357). While the authors suggest their data are novel because the rate remapping is not driven by externally perceived sensory information, the data seem entirely consistent with the idea that rate remapping (and splitter cells) represent different latent states. Surely this theory (which is supported by existing data) should be referenced earlier in the manuscript, i.e. in the Results section, to situate the findings within the context of the wider literature?

We thank the reviewer for this important clarification and for drawing our attention to the broader theoretical framework developed by Duvelle et al. (2023), in which splitter-cell activity is interpreted in terms of temporal context and latent-state inference. We now recognize that this framework may encompass not only classical trajectory-dependent splitter cells but also related forms of context-dependent activity, including the lap-specific representations described by Sun et al. (2020).

We have therefore added a brief discussion of these ideas to the Results section (lines 153–158), so that our findings are situated within this broader literature at an earlier stage of the manuscript. We have also substantially revised the Discussion (lines 474–500) to explain how our findings relate to, and are consistent with, temporal-context and latent-state accounts, while clarifying the additional information provided by our population-level and circuit analyses.

3. Regarding my third point in round 1: "Can the authors make clearer how this paper builds on previous studies that have applied optogenetic manipulations to NR to examine the effect of NR on hippocampus?..etc." The authors provide a detailed and clear response in the rebuttal but have only added citations into the manuscript. I recommend adding into the Discussion the section (or a version of it) from the rebuttal that starts "What our study adds..". This is to ensure that the novelty of the current results remain clear.

We apologize for not incorporating this explanation more fully into the previous version of the manuscript. We have now added sentences that explicitly explain how the present findings extend previous studies of NR function and clarify the specific contribution of our work (lines 537–546).

The fourth, fifth, sixth, seventh, eight and ninth point, together with minor comments from round 1 have been addressed. I have no additional comments for the authors.

Reviewer #2 (Remarks to the Author):

The authors have made substantial and constructive revisions, and many of my original concerns have been addressed. I have a few remaining minor points that should be clarified before publication.
Major issue: Histology and placement. I appreciate the authors' including animal-wise histological images for illustrating probe and fiber placements in the NR, as well as channel-wise cell/spike counts based on those estimated probe channel locations, but I am still not able to see convincing evidence that the fibers and probes in question are indeed terminating in the NR. Rat 521 shows displacement of Ca1 and the dentate overlying the targeted NR, which helps in reconstructing the probe trajectory. But where the probe terminates is unclear, and in other animals, I am not able to see any disrupted tissue whatsoever. This is a crucial piece of validation for this study, for reasons explained in the first review.
The following would help with this validation:
1. Side-by-side images with and without the drawn-on putative probe location, along with detailed annotations/descriptions of which specific tissue features the authors believe indicate probe/fiber-related tissue disruption.
2. Darkfield/autofluorescence images from these slides, potentially showing tissue damage.
3. A standalone experiment using, e.g., thermolytic lesions, dye-coated fibers, etc, to demonstrate consistency in targeting the NR in the authors' hands
4. If unmounted tissue from the animals in this study is still available, IHC data showing other markers of scarring around the putative probe/fiber sites (e.g. Iba1, GFAP, NeuN, Dapi, or other markers of tissue damage)

We thank the reviewer for raising this important concern and apologize that the histological evidence presented in the previous version was not sufficiently convincing.

As suggested by the reviewer, we acquired autofluorescence images of the relevant sections. However, these images did not reveal track-specific signals that could provide additional information about the probe locations (Reviewer Fig. 1). Unfortunately, no unmounted sections from the animals used in the present study remain available, and we are therefore unable to perform additional histological or immunohistochemical analyses of these specimens.

We have nevertheless carefully re-examined the complete set of histological sections. In the revised manuscript, we provide raw and annotated representative sections, high-contrast insets, and detailed descriptions of the tissue features used to reconstruct the probe and fiber trajectories.

Although one might expect the entire track to be visible continuously from dorsal to ventral brain regions within a single section, this is not always possible. Because the angle of sectioning may differ from the implantation angle, different portions of the

same track can appear in successive sections at different dorsoventral levels. To demonstrate this, we now provide serial sections showing how the trajectories of the Neuropixels probes and optic fibers can be followed across adjacent sections (Reviewer Fig. 2).

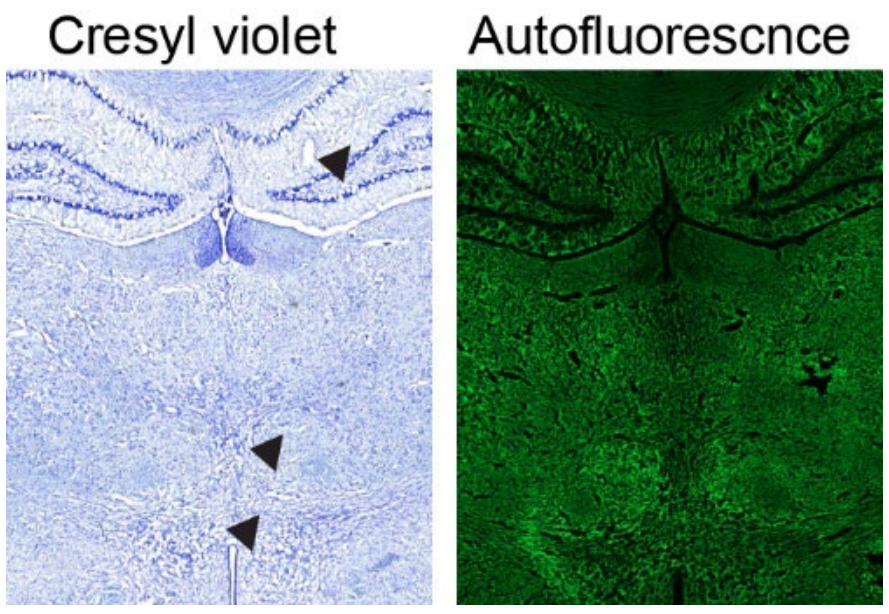

Reviewer Fig. 1. An example of an autofluorescence image at the probe track

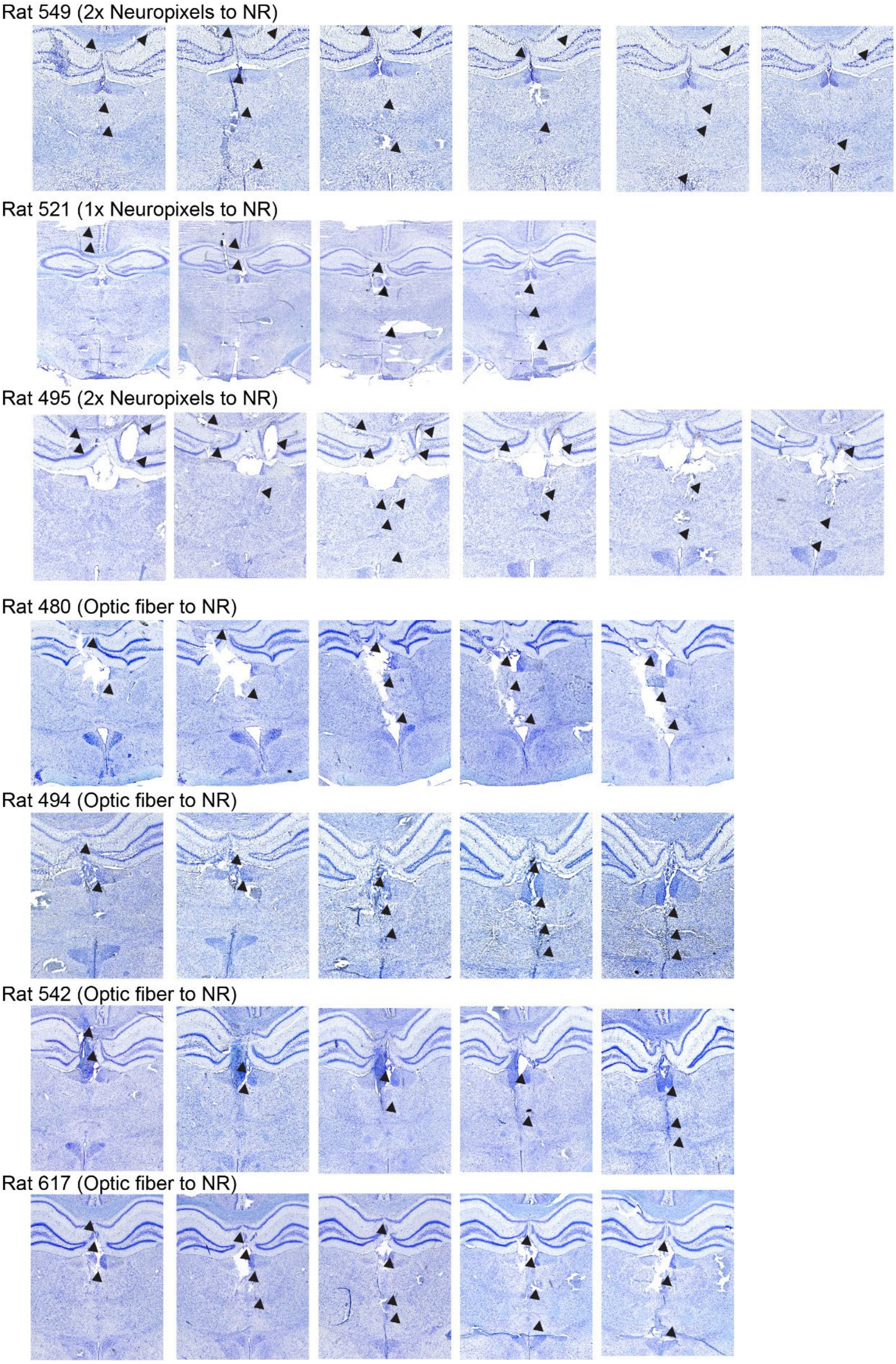

Reviewer Fig. 2. Serial sections showing the track of Neuropixels probes and optic fibers

To identify the tracks, we used histological features commonly employed in previous electrophysiological studies, including our own work using tetrodes and silicon

probes (e.g., Ito et al., *Nature*, 2015; Ito et al., *Neuron*, 2018; van Wijngaarden et al., *eLife*, 2020; Basu et al., *Nature*, 2021). These features include mechanical tissue disruption, narrow tissue separation along the implantation path, and local alignment or distortion of cells along it. Because of differences between the implantation and sectioning angles, the full trajectory is not necessarily apparent in any single section. However, individual portions of the track can be identified at higher magnification and followed across serial sections.

In addition, as suggested by the reviewer, we performed independent targeting validation in separate animals using Neuropixels probes and an optic fiber implanted at the same stereotaxic coordinates used to target NR (Reviewer Fig. 3). In these animals, the terminal portions of the tracks are clearly visible without the use of electrolytic lesions or exogenous histological markers. We hope that these additional examples demonstrate the reproducibility of our targeting of NR with both Neuropixels probes and optic fibers.

Rat 741 (Neuropixels to NR)

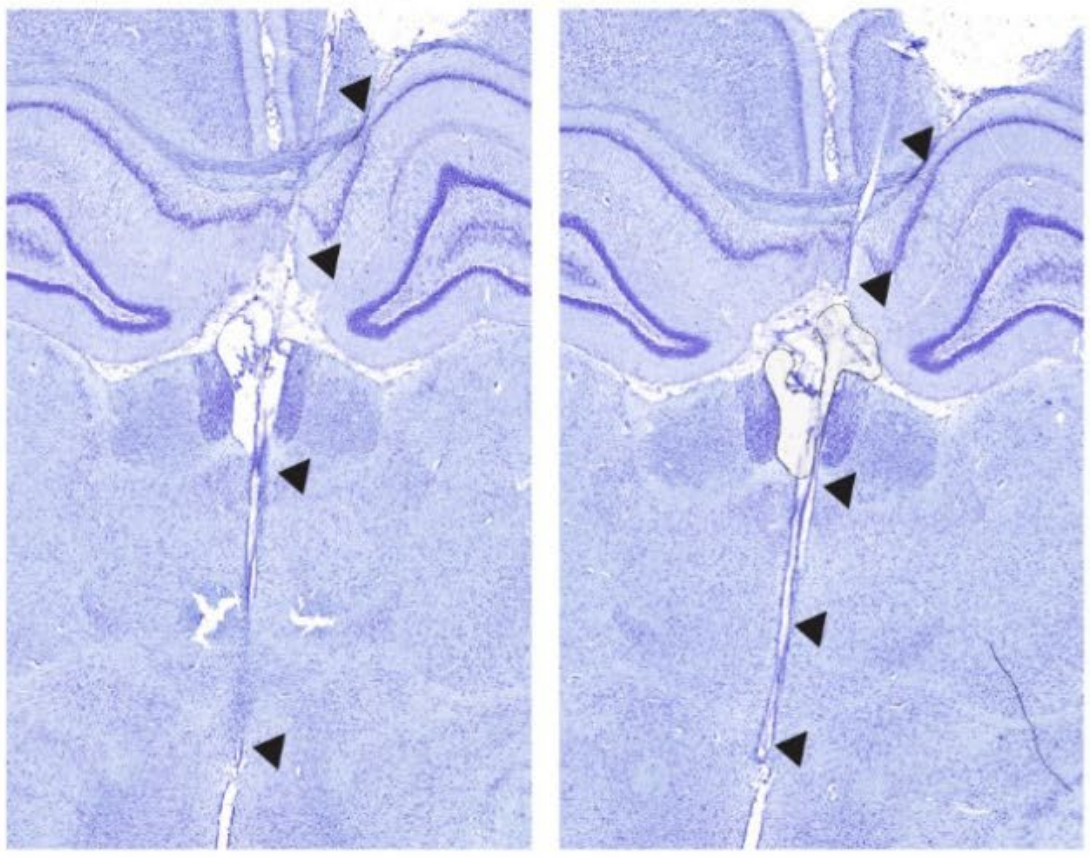

Rat 377 (Neuropixels to NR)

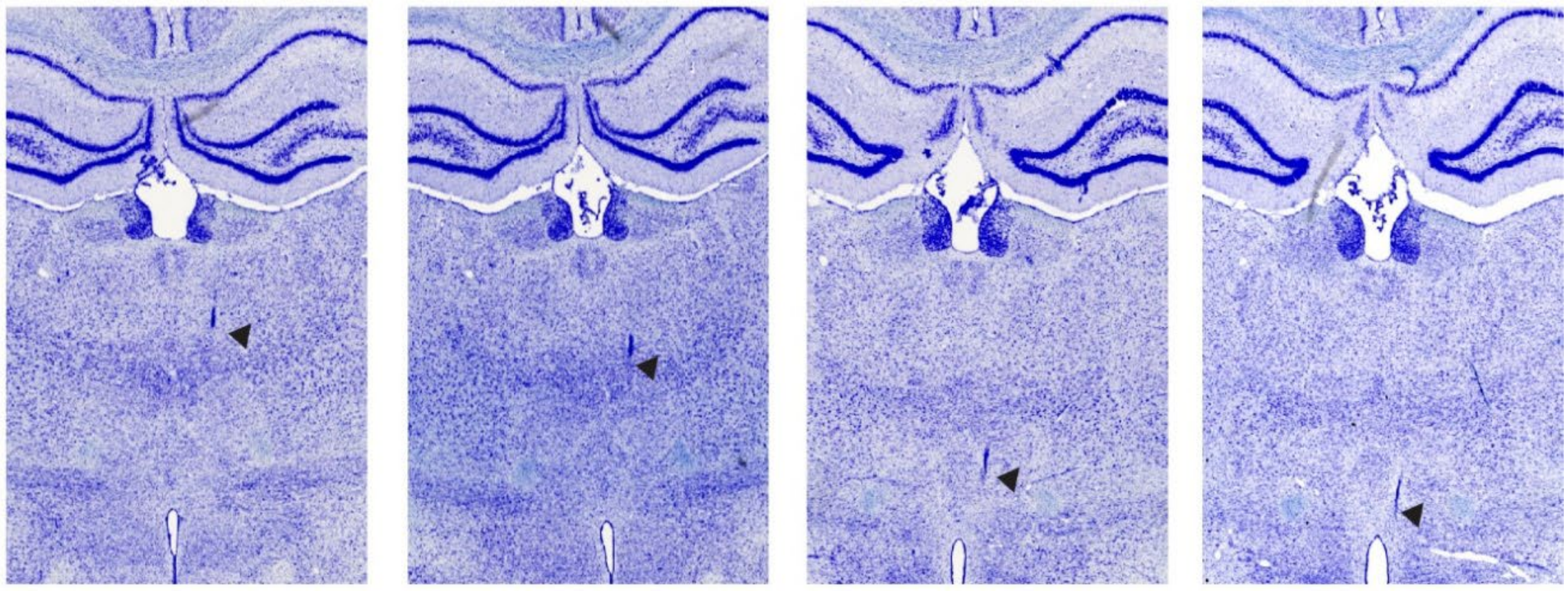

Rat 642 (Optic fiber to NR)

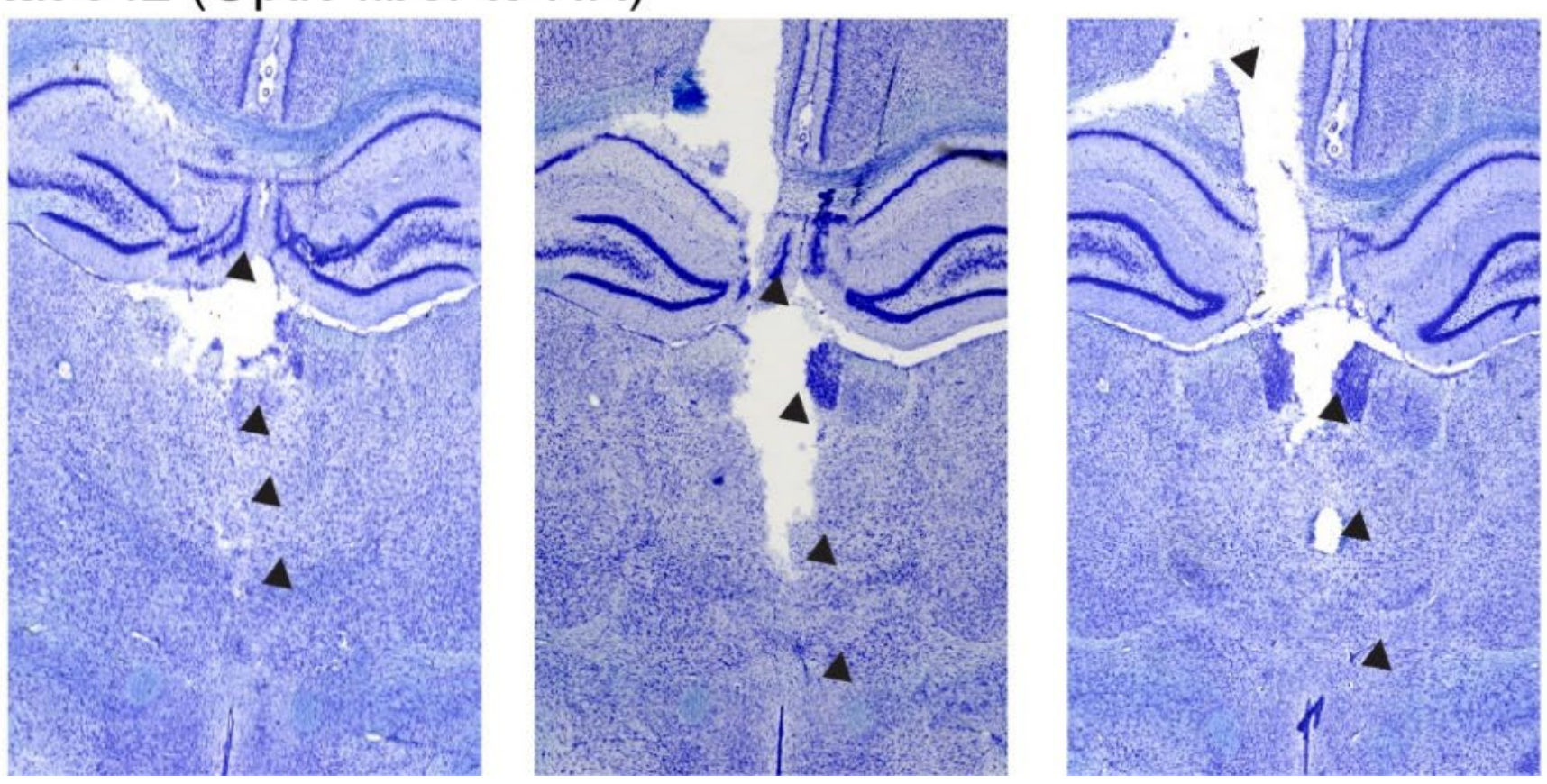

Reviewer Fig. 3. Serial sections showing the tracks of the Neuropixels probes and the optic fiber for animals not used in the present study

Minor issues

1. Language regarding sequence occupancy effects. The revised analyses of NR silencing effects on pre-navigation sequence occupancy are improved, particularly with the addition of raw occupancy curves and animal-level/mixed-effects statistics. However, the effects remain relatively subtle, and some of the statistical support appears close to threshold. I would therefore suggest softening language such as "abolished" or equivalent phrasing, where present, to "reduced," "attenuated," or "significantly diminished." This would better match the magnitude and robustness of the reported effects.

We agree and have revised the relevant wording throughout the manuscript to use more appropriately calibrated terms, such as "reduced," "attenuated," and "significantly diminished."

2. Clarification of spatial ROI/bin definitions in sequence analyses. The revised manuscript now provides a clearer description of the spatial normalization procedure used for pre-navigation sequence occupancy. However, I remain somewhat uncertain about whether the regions summarized in Fig. 2g and Fig. 5i/k were defined independently of the observed effects. In particular, if regions of interest were selected based on bins showing significant block or laser effects, then subsequent summary statistics over those same bins should be described as descriptive summaries rather than independent confirmatory tests. The authors should clarify whether these spatial windows were defined a priori, based on the task geometry, or based on the observed bin-wise effects. If the latter, the text and figure legends should make this explicit.

We thank the reviewer for this important clarification. The ROI used in Fig. 2g was not defined independently or a priori. Rather, it was defined from the contiguous region near the corresponding goal that showed a significant block-wise difference in sequence occupancy in Fig. 2f, as described in the Methods. We have therefore clarified that Fig. 2g provides a trial-resolved descriptive characterization of the spatial effect identified in Fig. 2f, rather than an independent confirmatory test. Specifically, whereas Fig. 2f identifies the spatial localization of the block-dependent effect using block-averaged occupancy, Fig. 2g illustrates how occupancy within this identified region evolves across individual trials around the block transitions.

The same ROI identified in Fig. 2f-g was applied unchanged to the NR-silencing data in Fig. 5k, so no bins were selected on the basis of the laser-session results. By contrast, the summary metric in Fig. 5i was motivated by the spatial pattern observed in Fig. 2e. Its two reference regions—the middle-goal location and the preceding journey midpoint—were defined from task geometry, and the identical occupancy-ratio calculation was applied to laser and no-laser sessions. Thus, although the choice of summary metric was motivated by Fig. 2e, no bins were selected on the

basis of the Fig. 5i laser effect. We have clarified these distinctions in the Methods and figure legends.

3. Robustness of sequence occupancy results to spatial window choice. Relatedly, because the pre-navigation sequence occupancy effects are spatially local and modest in magnitude, the authors should clarify whether the main conclusions are robust to reasonable changes in the goal-centered window definition. The added analyses improve interpretability, but a brief statement indicating that the effects are not dependent on one particular bin grouping or ROI definition would strengthen confidence in the result.

The spatial localization of the effect is established directly by the bin-wise analysis in Fig. 2f and therefore does not depend on the ROI subsequently used for the trial-wise summary in Fig. 2g. As shown in Reviewer Fig. 3 of our previous response letter, the occupancy asymmetry in Fig. 2e was preserved when the journey-midpoint reference positions were shifted toward the middle goal. This clarification has been added to the relevant figure legends.

4. Pathway-level causal language. The authors have substantially improved the language regarding the mPFC->NR->CA1 pathway, particularly in the Abstract and Results. However, a few statements in the Discussion still appear to imply a more specific pathway-level causal mechanism than is directly demonstrated. The data show that mPFC and NR both represent goal-state information, and that NR activity is required for robust CA1 goal-state coding. They do not directly establish that mPFC input to NR, or serial mPFC->NR->CA1 transmission, is the source of the CA1 signal. I would suggest one additional pass through the Discussion to replace phrases such as "this prefrontal-thalamic signal" with more precise wording, for example "goal-state representations in mPFC and NR," "NR-dependent goal-state activity," or "prefrontal-thalamic activity involving NR." This would preserve the authors' model while keeping the causal claim aligned with the experiments performed.

We agree and have revised the corresponding words throughout the manuscript.

5. Figure and legend clarity. The revised manuscript is clearer than the original, but some figure legends remain dense and difficult to parse. For panels involving shuffled controls, pseudo-block controls, or normalized spatial occupancy, the legends should explicitly state what was shuffled or normalized, what was held constant, and what statistical unit was used. This is particularly important for the new control analyses, which are valuable but will be difficult for readers to evaluate unless the logic is spelled out in the figure legends as well as in the Methods.

We have carefully revised the figure legend, clarifying normalization and shuffling procedures.

Overall, I view these as minor issues of clarification and calibration rather than major concerns. The revised manuscript is substantially improved, and the additional analyses address many of the central concerns raised in review.
