# Supplementary material for: Prefrontal–thalamic goal states organize spatially aligned hippocampal maps
Source: Nat Commun. 2026 Aug 29;17:9233. doi: 10.1038/s41467-026-77240-6 (PMC13526029; doi:10.1038/s41467-026-77240-6)
Supplement: Supplementary file 4 [file 41467_2026_77240_MOESM5_ESM.pdf]

Panel	Description	p-value	Test
Fig 1g		0.6766	Wilcoxon signed-rank test
Fig. 1j top	B1-B2 vs B1-B3	1.40E-04	Wilcoxon signed-rank test
	B1-B3 vs within	0.1451	Wilcoxon signed-rank test
Fig. 1j bottom	B1-B2 vs B1-B3	0.0078	Wilcoxon signed-rank test
	B1-B3 vs within	0.4609	Wilcoxon signed-rank test
Fig. 1k left	Pos vs. Block	1.23E-05	Wilcoxon signed-rank test
	Block vs. Shuffle	1.23E-05	Wilcoxon signed-rank test
Fig. 1k right	Pos vs. Block	1.23E-05	Wilcoxon signed-rank test
	Block vs. Shuffle	0.7775	Wilcoxon signed-rank test
Fig. 2c left	Frequency	0.6297	Kruskal-Wallis test
Fig. 2c right	Duration	0.3677	Kruskal-Wallis test
Fig. 2e left	Middle-goal	4.93E-04	Wilcoxon signed-rank test
Fig. 2e right	End-goal	0.0066	Wilcoxon signed-rank test
Fig. 2f left	Proximal first	0.0043	Wilcoxon signed-rank test
Fig. 2f right	Distal first	0.0147	Wilcoxon signed-rank test
Fig. 3d top left	mPFC	2.44E-04	Wilcoxon signed-rank test
Fig. 3d goal mPFC	Pos vs. Block	2.44E-04	Wilcoxon signed-rank test
	Block vs. shuffle	2.44E-04	Wilcoxon signed-rank test
Fig. 3d pos mPFC	Pos vs. Block	4.88E-04	Wilcoxon signed-rank test
	Block vs. shuffle	0.8394	Wilcoxon signed-rank test
Fig. 3d top right	NR	4.88E-04	Wilcoxon signed-rank test
Fig. 3d goal NR	Pos vs. Block	4.88E-04	Wilcoxon signed-rank test
	Block vs. shuffle	4.88E-04	Wilcoxon signed-rank test
Fig. 3d pos NR	Pos vs. Block	4.88E-04	Wilcoxon signed-rank test
	Block vs. shuffle	0.8501	Wilcoxon signed-rank test
Fig. 3e top left	CA1 vs. mPFC	1.25E-05	Wilcoxon rank-sum test
	mPFC vs. NR	0.4628	Wilcoxon rank-sum test
Fig. 3e top right	CA1 vs. mPFC	0.7818	Wilcoxon rank-sum test
	mPFC vs. NR	0.221	Wilcoxon rank-sum test
Fig. 3e bottom left	CA1 vs. mPFC	5.27E-06	Wilcoxon rank-sum test
	mPFC vs. NR	0.0535	Wilcoxon rank-sum test
Fig. 3e bottom right	CA1 vs. mPFC	1	Wilcoxon rank-sum test
	mPFC vs. NR	0.0114	Wilcoxon rank-sum test
Fig. 4c	mPFC	2.44E-04	Wilcoxon signed-rank test
	NR	4.88E-04	Wilcoxon signed-rank test
	CA1	1.23E-05	Wilcoxon signed-rank test
Fig. 4g	All sessions	1.02E-04	Wilcoxon signed-rank test
Fig. 5c	No laser vs. Laser	0.1688	Wilcoxon rank-sum test
Fig. 5e top left	B1-B2 vs. B1-B3	1.22E-04	Wilcoxon signed-rank test
	B1-B3 vs. Within	0.4631	Wilcoxon signed-rank test
Fig. 5e top right	B1-B2 vs. B1-B3	0.0645	Wilcoxon signed-rank test
	B1-B3 vs. Within	0.5566	Wilcoxon signed-rank test
Fig. 5e bottom left	Difference	0.0128	Wilcoxon rank-sum test
		0.037584	repeated-measures ANOVA (laser condition x block-pair interaction)
Fig. 5f	Position decoding	0.6605	Wilcoxon rank-sum test
Fig. 5h	No laser vs. Laser	0.0151	Wilcoxon rank-sum test
		0.013	linear mixed-effects model
Fig. 5i	Ratio	0.0376	Wilcoxon rank-sum test
		0.0485	linear mixed-effects model (permutation test)
Fig. 5j	Ratio	0.5387	Wilcoxon rank-sum test
Fig. 5k	Block 1 vs. Block 2	0.4316	Wilcoxon signed-rank test

Fig. 5l	No laser	3.66E-04	Wilcoxon signed-rank test
	Laser	0.1934	Wilcoxon signed-rank test
ExtDataFig. 3a top left	B1 vs. B2	1.62E-04	Wilcoxon signed-rank test
	B2 vs. B3	0.0574	Wilcoxon signed-rank test
ExtDataFig. 3a bottom left	B1 vs. B2	7.22E-05	Wilcoxon signed-rank test
	B2 vs. B3	0.011	Wilcoxon signed-rank test
ExtDataFig. 3c	Blockwise	0.765	Kruskal-Wallis test
	General	0.2822	Kruskal-Wallis test
ExtDataFig. 3d	R2y	0.0054	Wilcoxon signed-rank test
	R2x	1.13E-04	Wilcoxon signed-rank test
ExtDataFig. 3e	R2	0.0961	Kruskal-Wallis test
ExtDataFig. 3f	R2	1.96E-04	Wilcoxon signed-rank test
	Decoding error	1.96E-04	Wilcoxon signed-rank test
ExtDataFig. 3h	Spatial	3.61E-05	Friedman test
	Nonspatial	3.01E-04	Friedman test
ExtDataFig. 3i	B1B2 vs. B1B3	1.40E-04	Wilcoxon signed-rank test
	B1B3 vs. B1B2 shuffle	0.0049	Wilcoxon signed-rank test
	B1B2 shuffle vs. B1B3 shuffle	0.8191	Wilcoxon signed-rank test
ExtDataFig. 3j left	Full vs. Pos	0.3395	Wilcoxon signed-rank test
	Pos vs. Goal	1.23E-05	Wilcoxon signed-rank test
ExtDataFig. 3j right	Full vs. Pos	1.23E-05	Wilcoxon signed-rank test
	Pos vs. Goal	1.23E-05	Wilcoxon signed-rank test
ExtDataFig. 3k	Prior to middle-goal	0.0078	Wilcoxon signed-rank test
	Prior to trackend-goal	0.1641	Wilcoxon signed-rank test
ExtDataFig. 3k proximal first	B1 vs. B2	0.0078	Wilcoxon signed-rank test
	B2 vs. B3	0.0195	Wilcoxon signed-rank test
ExtDataFig. 3k distal first	B1 vs. B2	0.0977	Wilcoxon signed-rank test
	B2 vs. B3	0.0391	Wilcoxon signed-rank test
ExtDataFig. 6c mPFC top left	Full vs. Pos	2.44E-04	Wilcoxon signed-rank test
	Pos vs. Goal	2.44E-04	Wilcoxon signed-rank test
ExtDataFig. 6c mPFC top right	Pos vs. Block	2.44E-04	Wilcoxon signed-rank test
	Block vs. Shuffle	2.44E-04	Wilcoxon signed-rank test
ExtDataFig. 6c mPFC bottom left	Full vs. Pos	2.44E-04	Wilcoxon signed-rank test
	Pos vs. Goal	2.44E-04	Wilcoxon signed-rank test
ExtDataFig. 6c mPFC bottom right	Pos vs. Block	2.44E-04	Wilcoxon signed-rank test
	Block vs. Shuffle	2.44E-04	Wilcoxon signed-rank test
ExtDataFig. 6c NR top left	Full vs. Pos	4.88E-04	Wilcoxon signed-rank test
	Pos vs. Goal	4.88E-04	Wilcoxon signed-rank test
ExtDataFig. 6c NR top right	Pos vs. Block	4.88E-04	Wilcoxon signed-rank test
	Block vs. Shuffle	0.3804	Wilcoxon signed-rank test
ExtDataFig. 6c NR bottom left	Full vs. Pos	4.88E-04	Wilcoxon signed-rank test
	Pos vs. Goal	9.77E-04	Wilcoxon signed-rank test
ExtDataFig. 6c NR bottom right	Pos vs. Block	4.88E-04	Wilcoxon signed-rank test
	Block vs. Shuffle	4.88E-04	Wilcoxon signed-rank test
ExtDataFig. 6c CA1 top left	Full vs. Pos	2.66E-04	Wilcoxon signed-rank test
	Pos vs. Goal	1.23E-05	Wilcoxon signed-rank test
ExtDataFig. 6c CA1 top right	Pos vs. Block	1.23E-05	Wilcoxon signed-rank test
	Block vs. Shuffle	0.1353	Wilcoxon signed-rank test
ExtDataFig. 6c CA1 bottom left	Full vs. Pos	1.23E-05	Wilcoxon signed-rank test
	Pos vs. Goal	1.23E-05	Wilcoxon signed-rank test
ExtDataFig. 6c CA1 bottom right	Pos vs. Block	1.23E-05	Wilcoxon signed-rank test
	Block vs. Shuffle	1.23E-05	Wilcoxon signed-rank test

ExtDataFig. 6d	ACC vs. PL	0.0061	Wilcoxon signed-rank test
	PL vs. IL	0.0105	Wilcoxon signed-rank test
ExtDataFig. 7a	Block axis separation	0.0117	Wilcoxon signed-rank test
ExtDataFig. 7e	mPFC running	4.88E-04	Wilcoxon signed-rank test
	mPFC stationary	0.2163	Wilcoxon signed-rank test
	NR running	0.0068	Wilcoxon signed-rank test
	NR stationary	0.1099	Wilcoxon signed-rank test
	CA1 running	2.96E-04	Wilcoxon signed-rank test
	CA1 stationary	1.13E-04	Wilcoxon signed-rank test
	diff running mPFC-CA1	0.4418	Wilcoxon rank-sum test
	diff running NR-CA1	0.9354	Wilcoxon rank-sum test
	diff stationary mPFC-CA1	0.0035	Wilcoxon rank-sum test
	diff stationary NR-CA1	0.0497	Wilcoxon rank-sum test
ExtDataFig. 7f	Stationary Spatial	1.77E-05	Wilcoxon signed-rank test
	Stationary Nonspatial	2.54E-05	Wilcoxon signed-rank test
	Running Spatial	0.0173	Wilcoxon signed-rank test
	Running Nonspatial	8.09E-05	Wilcoxon signed-rank test
	Stationary	0.1036	Wilcoxon signed-rank test
	Running	0.326	Wilcoxon signed-rank test
ExtDataFig. 7g	Stationary High rate	1.77E-05	Wilcoxon signed-rank test
	Stationary Low rate	2.86E-05	Wilcoxon signed-rank test
	Running High rate	2.96E-04	Wilcoxon signed-rank test
	Running Low rate	0.0018	Wilcoxon signed-rank test
	Stationary	0.0179	Wilcoxon signed-rank test
	Running	0.0186	Wilcoxon signed-rank test
ExtDataFig. 8a top right	Block 1 No laser vs. Laser	0.17	Wilcoxon rank-sum test
	Block 2 No laser vs. Laser	0.8374	Wilcoxon rank-sum test
	Block 3 No laser vs. Laser	0.0649	Wilcoxon rank-sum test
ExtDataFig. 8a	Running speed	0.4292	Wilcoxon rank-sum test
	Task performance	0.5193	Wilcoxon rank-sum test
ExtDataFig. 8b	Decoding error - No laser	0.9442	Kruskal-Wallis test
	Decoding error - Laser	0.802	Kruskal-Wallis test
	R2 position	0.334	Wilcoxon rank-sum test
	R2 direction	0.93	Wilcoxon rank-sum test
ExtDataFig. 8c	Frequency	0.578	Wilcoxon rank-sum test
	Duration	0.5387	Wilcoxon rank-sum test
ExtDataFig. 9b	Rate remapping Spatial	0.0201	Friedman test
	Rate remapping Nonspatial	0.0201	Friedman test
	Global remapping	0.4386	Friedman test
ExtDataFig. 9c	Rate remapping Spatial	0.5064	Wilcoxon rank-sum test
	Rate remapping Nonspatial	0.19107	Wilcoxon rank-sum test
ExtDataFig. 9f	Proportion	0.43858	Friedman test
ExtDataFig. 9g	No laser Spatial	0.006706	Friedman test
	No laser Nonspatial	0.001946	Friedman test
	Laser Spatial	0.052808	Friedman test
	Laser Nonspatial	1	Friedman test
ExtDataFig. 10d	Full dimensions position	0.095159	Wilcoxon rank-sum test
	Full dimensions goal	0.028108	Wilcoxon rank-sum test
	LDA position	0.33397	Wilcoxon rank-sum test

	LDA goal	0.020729	Wilcoxon rank-sum test
ExtDataFig. 10e	Chance level	0.53868	Wilcoxon rank-sum test
ExtDataFig. 10f	Decoding	0.043372	Wilcoxon rank-sum test
ExtDataFig. 10j	CA1 (9 animals)	1.23E-05	Wilcoxon signed-rank test
	CA1 No laser	0.000122	Wilcoxon signed-rank test
	CA1 Laser	0.001953	Wilcoxon signed-rank test
	Decoding	0.12074	Wilcoxon rank-sum test
ExtDataFig. 10k Left	Goal vs. Sequence	1.23E-05	Wilcoxon signed-rank test
	Sequence vs. Shuffle	0.002699	Wilcoxon signed-rank test
ExtDataFig. 10k Right	Goal vs. Sequence	0.000383	Wilcoxon signed-rank test
	Sequence vs. Shuffle	1.23E-05	Wilcoxon signed-rank test
